# Oxidative stress induced by DMSA-IONPs impairs breast cancer cell migration and paracrine cell communication

**DOI:** 10.1186/s12951-026-04412-3

**Published:** 2026-04-09

**Authors:** Neus Daviu, Carla Graciano-Casero, Domingo F. Barber

**Affiliations:** 1 Department of Immunology and Oncology, and Nanobiomedicine Initiative, Centro Nacional de Biotecnología (CNB-CSIC), Darwin 3, Madrid, 28049 Spain; 2 Faculty of Experimental Sciences, Francisco de Vitoria University (UFV), Ctra. Pozuelo-Majadahonda Km 1, 800, Pozuelo de Alarcón, 28223 Spain

**Keywords:** Cancer, ROS, Actin oxidation, Tumor cell migration and invasion, Unconventional pathways for protein secretion, Paracrine communication

## Abstract

**Background:**

The migration of tumor cells requires a complex interplay of these cells with their microenvironment. Significantly, these migrating (metastatic) cells undergo metabolic and oxidative changes that render them more sensitive to shifts in their redox homeostasis. Nanoparticle (NP)-based pro-oxidant therapies are strategies that can potentially influence cell viability and migration. Among these, iron oxide NPs (IONPs) stand out due to their biocompatibility and the fact they induce ROS production through the Fenton Reaction. Here we investigate the possibility of using dimercaptosuccinic acid (DMSA)-coated IONPs as therapeutic tools to combat breast cancer (BC) cell migration and metastasis, influencing the paracrine communication between tumor and endothelial cells.

**Results:**

DMSA-IONPs affected the migration and invasion of a BC cell line by modulating the actin cytoskeleton. ROS production induced by DMSA-IONPs provoked actin carbonylation, which drives actin cytoskeletal rearrangements that reduces the cell area and the number of invadosomes per cell. Moreover, the presence of DMSA-IONPs in endo-lysosomal compartments affected unconventional pathways for protein secretion (UCPS), stimulating the release of late endosomes or multivesicular bodies, and blocking the release of lysosomes and autophagosomes. These effects alter secretory autophagy and lysosomal secretory pathways, which together influence the migration and chemotaxis of endothelial cells, modulating paracrine communication.

**Conclusions:**

DMSA-IONPs can potentially inhibit the metastasis of a model BC cell line by directly affecting BC cell migration and indirectly altering the migration of endothelial cells.

**Graphical abstract:**

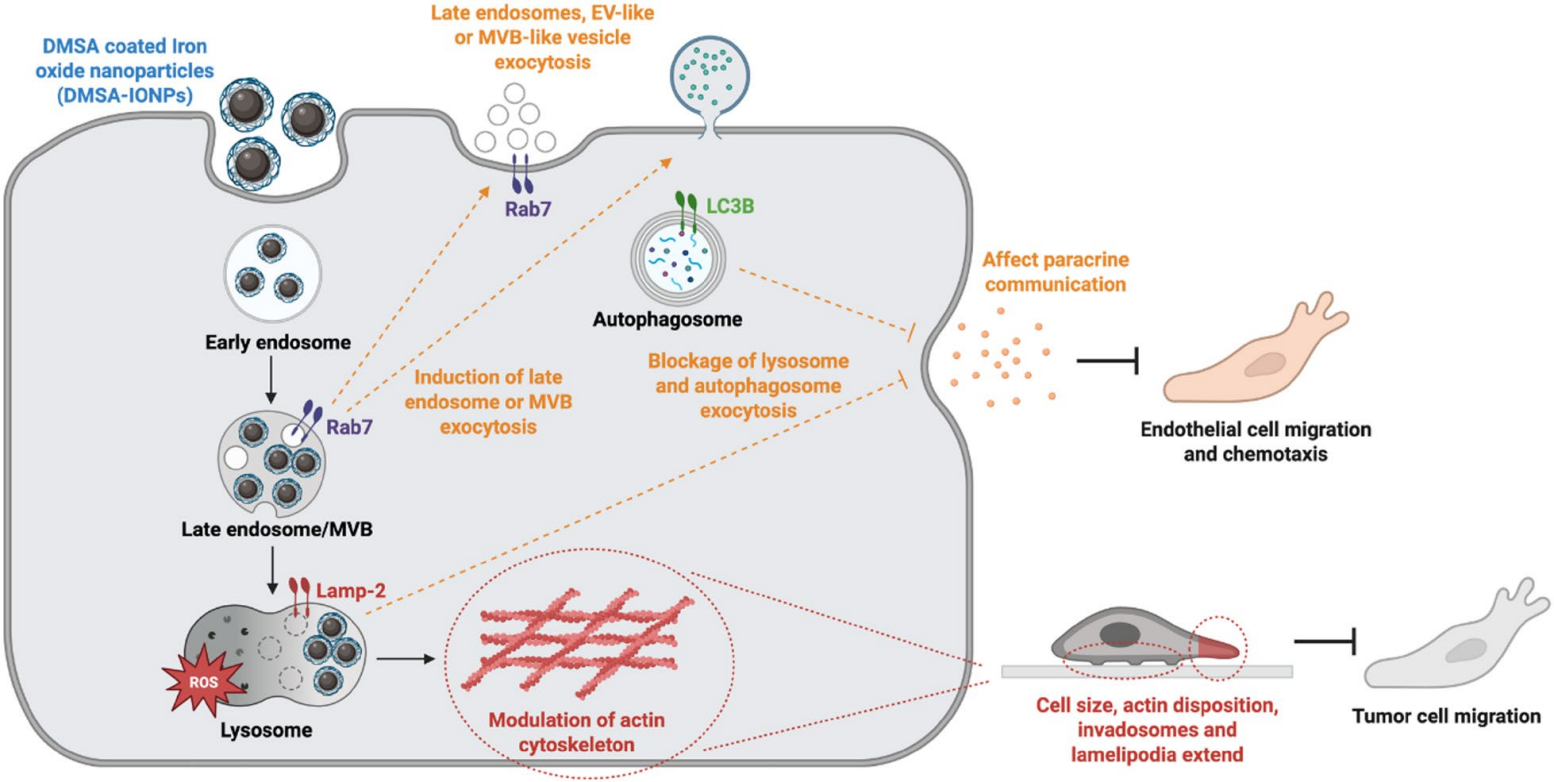

**Supplementary Information:**

The online version contains supplementary material available at 10.1186/s12951-026-04412-3.

## Background

Breast cancer (BC) is the second most common cancer worldwide, and it is the number one cancer and one of the leading causes of death in women [1]. The poor prognosis of BC is a consequence of the migration and spreading of cancer cells from the breast lobules into surrounding tissues (e.g. the lymph nodes) or distant organs, events that underlie metastasis [2]. For these cells to migrate, their actin cytoskeleton must undergo dynamic rearrangements that polarize the cell and that control cell adhesion, cell contractions and the retraction of the cell from the front to the rear [3]. The appearance of protrusive cell fronts or lamellipodia is another key step in this directed migration and invasion that is regulated by a reorganization of the actin cytoskeleton [4]. Lamellipodia are thin, sheet-like membrane protrusions at the leading edge of the cell, the formation of which is driven by the activity of actin binding proteins (ABPs) and that requires abundant adenosine triphosphate (ATP) [5]. Consequently, mitochondria are commonly transported to the leading-edge to ensure a good supply of ATP, calcium and other key metabolites, where they also release reactive oxygen species (ROS). Thus, the redistribution and structural reorganization of mitochondria plays a crucial role in cancer cell metastasis and migration [6]. Mitochondrial structure and shape is tightly controlled through fission and fusion events that dictate their disposition as small punctuate spheres or as a more reticular network [7]. Mitochondrial fragmentation has at times been linked to enhanced transport to the leading-edge, thereby facilitating cancer cell migration and metastasis [8, 9]. However, it has also been proposed that mitochondrial fission inhibits cancer cell proliferation and migration [10].

Exogenous ROS, and that produced intracellularly, can influence cell migration and adhesion. Small quantities of ROS can induce reversible cysteine oxidation of proteins, affecting their activity, stability, localization or interaction with other proteins [11, 12]. When ROS levels exceed given thresholds, the redox balance is disturbed and significant oxidation may occur, not only of proteins but also of DNA and lipids, ultimately provoking cell death [13]. Since mitochondria are intrinsic sources of ROS, mitochondrial dynamics are tightly regulated by a number of signaling pathways that can in turn be regulated by redox signals. Thus, depending on the levels of ROS inside the cell, redox sensors might act and induce mitochondrial fusion or fission [14]. Moreover, oxidative stress can also regulate actin cytoskeleton rearrangements by altering the oxidation of actin itself, or that of signaling molecules or ABPs like myosin II, cofilin-1, gelsolin and L-plastin (LPL) [15]. For instance, actin, LPL and cofilin oxidation dampens cell migration and invasion [16–18], whereas integrin α7β1 oxidation enhances its binding to laminin and cell migration [19]. Thus, ROS may play different roles in cell migration depending on their location, amounts and nature.

The hostile environment cells face when they detach from primary tumors and enter the circulating blood, where the levels of oxidants are higher, makes migration and metastasis inefficient [20]. Cancer cells must regulate their metabolic and redox status during metastasis, as redox stress threatens their survival [21] and this imbalance renders them more sensitive to changes to their redox homeostasis. When mice that serve as cancer models are treated with antioxidants they have more circulating cancer cells [22–24], whereas inducing oxidative stress appears to compromise the survival of circulating cancer cells and diminish cell metastasis [25]. As oxidative stress limits cancer cell survival during metastasis, pro-oxidant therapies that exacerbate oxidative stress may potentially combat metastatic cancer. Hence, the use of iron oxide nanoparticles (IONPs) to induce oxidative stress is gaining interest, as their constituent iron ions (Fe^2+^ and Fe^3+^) exacerbate ROS production by catalyzing the conversion of cellular H_2_O_2_ into hydroxyl radicals (OH) through Fenton and Haber-Weiss reactions [26, 27]. The safety and biocompatibility of IONPs means they can be used in biomedical applications [28]. Moreover, as different drugs can be attached to their surface they can be used in processes like targeted drug delivery [29]. Cancer cells can take up IONPs or other types of NPs through phagocytosis or pinocytosis, entering the endo-lysosomal system, and passing from early endosomes towards late endosomes and lysosomes [30]. The presence of IONPs inside acidic organelles like lysosomes promotes their degradation and the release of iron ions, which can then accumulate in the cytosol. Increasing the labile iron pool in the cytosol triggers susceptibility to the Fenton reaction as these ions can oxidize H_2_O_2_ to ·OH^−^ [31]. Thus, the presence of IONPs inside cells has been linked to ROS generation, specifically in lysosomes [32].

During tumor progression and cancer metastasis, new blood vessels are formed that play a crucial role in tumor growth, invasion and metastasis. To form a mature vascular network endothelial cells are activated, proliferate and migrate [33]. As such, a complex and dynamic interplay is established between the tumor and stromal cells in the tumor microenvironment, characterized by the secretion of multiple factors, such as cytokines and chemokines that influence tumor progression [34]. The conventional secretory pathway (CSP) consists of co-translation in the endoplasmic reticulum (ER), transport to the Golgi apparatus and constitutive or regulated secretion out of the cell [35]. By contrast, unconventional pathways (UCPS) drive protein secretion without implicating ER-Golgi transit. One UCPS is secretory autophagy, which like degradative autophagy relies on autophagosome formation, yet instead of fusing with a lysosome to form an autolysosome these structures can fuse with the plasma membrane (PM) for extracellular secretion. These autophagosomes can also fuse with late endosomes or multivesicular bodies (MVBs), which in turn form amphisomes that can release their cargoes extracellularly [36]. Thus, the autophagic and endo-lysosomal systems play critical roles in the extracellular release of proteins.

Some proteins can also be released in extracellular vesicles (EVs), structures derived from endosomal trafficking where early endosomes form late endosomes and MVBs, and these are subsequently mobilized to the PM and released through exocytosis [37]. Although EVs were initially believed to handle cell waste, they are now known to be critical mediators of intercellular communication that exchange proteins, RNAs, DNAs, lipids and metabolites [38]. Secreted EVs, chemokines and cytokines are recognized mediators between cancer cells and stromal cells. Lysosomes can also participate in secretory pathways, secreting their content upon fusion with the PM. Lysosome exocytosis plays a crucial role in PM repair [39] and in specific membrane remodeling, such as the formation of phagocytic structures that surround and engulf large cellular particles in macrophages [40]. This remodeling is important for the neurite outgrowth and elongation associated with developing neuronal processes [41], and also in the formation of large protrusions that promote tissue invasion [42].

We previously showed how dimercaptosuccinic (DMSA)-coated IONPs (DMSA-IONPs) prompt ROS production in different cancer cells, influencing mitochondrial shape and metabolism in the MDA-MB-231 BC cell line [43]. DMSA-IONPs have been considered promising tools due to their limited toxicity and long-term bioavailability [44], suggesting that they may be suitable for anti-cancer therapies in vivo. As oxidative stress and mitochondria are directly linked to cell migration, here we evaluated the potential of DMSA-IONPs to affect BC cell migration. Moreover, as IONPs are thought to be handled by the endo-lysosomal system upon entry into cells [45], we also evaluated changes to UCPS and to paracrine communication with cells in the tumor microenvironment. Hence, we studied the use of DMSA-IONPs to induce oxidative stress in the metastatic MDA-MB-231 BC cell line, modulating the migration of these cancer cells and their communication with surrounding cells. Oxidative stress induced by DMSA-IONPs affected BC migration and invasion, modulating the actin cytoskeleton. Moreover, the DMSA-IONPs used affected UCPS, modifying lysosome and autophagosome exocytosis, and thereby modulating secretory autophagy and the extracellular release of certain factors. This modification directly affected paracrine communication between MDA-MB-231 BC cells and SVEC4-10 endothelial cells, and as seen previously these DMSA-IONPs dampened the migration and chemotaxis of this endothelial cell line. In conclusion, DMSA-IONPs stand out as agents that can potentially be used in pro-oxidant therapies against metastatic cancers, not only modulating cell migration but also, modifying the communication between cancer cells and their microenvironment due to their effects on the endo-lysosomal system.

## Materials and methods

### Synthesis and characterization of DMSA-IONPs

IONPs were synthesized as described previously, according to Massart’s co-precipitation protocol [46], and the iron cores obtained were then coated with DMSA as described elsewhere [47]. Following coating procedure, the DMSA-IONP suspension was dialyzed with a 14 kDa dialysis membrane for 2 days to remove unbound DMSA. At the end of the dialysis, the pH was adjusted to 7 and DMSA-IONPs were filtered through a 0.22 μm filter for sterility. To assess the shape and distribution of the DMSA-IONPs, they were analyzed by transmission electron microscopy (TEM) on a JEOL JEM 1011 transmission electron microscope connected to a Gatan ES1000Ww camera. The size of the DMSA-IONPs in suspension was assessed by Dynamic Light Scattering (DLS) using a Zetasizer Nano ZS (Malvern) apparatus.

### Cell cultures

The human MDA-MB-231 BC cell line (ATCC_HTB-26) was purchased from the ATCC and cultured in DMEM (Dulbecco’s modified Eagle’s medium) supplemented with 10% FBS (Fetal Bovine Serum), 1% non-essential amino acids, 2 mM L-Glutamine, 1 mM Sodium Pyruvate, 100 IU/ml Penicillin and 100 mg/ml streptomycin. Cell cultures were maintained at 37 °C in an atmosphere of 5% CO_2_ and at 90% relative humidity. All cell lines have been used at the passage number range of 4–20 for cell culture experiments.

### Intracellular location of DMSA-IONPs in MDA-MB-231 cells assessed by TEM

MDA-MB-231 cell lines were seeded in petri dishes for 24 h, after which DMSA-IONPs (125 µgFe/ml) were added to the cells for 3–24 h. After treatment, the cells were washed three times with phosphate buffer saline (PBS) to remove the non-internalized DMSA-IONPs and fixed for 2 h at room temperature (RT) in 2% glutaraldehyde and 1% tannic acid diluted in 0.4 M HEPES (pH 7.2). The cells were recovered by gentle scraping and resuspended in HEPES buffer, post-fixed at 4 °C with 1% osmium tetroxide for 1 h and 2% uranyl acetate for 30 min, dehydrated in a series of acetone solutions and gradually infiltrated with Epon resin. The resin was allowed to polymerize (60 °C, 48 h) and ultrathin Sects. (60–70 nm) were obtained with a diamond knife mounted on a Leica EM UC6 ultramicrotome. The sections were attached to a formvar/carbon-coated gold grid and visualized on a JEOL-1400 flash 100 kV transmission electron microscope, acquiring images at different magnifications with a Gatan ONE VIEW camera.

To study DMSA-IONPs endocytosis, the presence of clathrin- and caveolin-like structures was visualized after a 3 h treatment. Likewise, to assess DMSA-IONPs localization in early endosomes, the 3 h treatment was also used. Conversely, to visualize their intracellular accumulation in late endosomes and autolysosomes, and to study the exocytosis of EV- and MVB-like structures, a 24 h treatment was used.

### Quantification of intracellular and extracellular iron after IONP treatment

To quantify DMSA-IONP internalization, the intracellular iron content of the cells was quantified by ICP-OES (inductively coupled plasma – optical emission spectrometry). To assess if N-acetyl-cysteine (NAC) affects DMSA-IONP internalization, MDA-MB-231 cells were seeded in 12-well plates, cultured for 24 h, and then treated with DMSA-IONPs (125 µg/ml) for 24 h in the presence or absence of NAC (5 mM) added 2 h prior to the DMSA-IONPs and present in culture for the 24 h. The cells were then rinsed three times with PBS to remove non-internalized IONPs, collected with trypsin-EDTA and counted in a Neubauer chamber. The cell suspension was centrifuged at 1200 rpm for 5 min, and digested for 1 h at 90 °C and overnight (ON) at RT with 1 ml of HNO_3_ (63%). After digestion, the volume was brought up to 10 ml with distilled water and the iron content per cell was measured by ICP-OES (Perkin Elmer-2400). Likewise, to study the internalization of DMSA-IONPs by SVEC4-10 cells, these cells were seeded in 12-well plates, cultured for 24 h and the cells were then treated for 24 h with DMSA-IONPs (60–125 µg/ml) for 24 h. The cells were then recovered and their iron content quantified by ICP-OES as described above.

To assess the mechanism of DMSA-IONP internalization by MDA-MB-231 cells, the cells were treated with DMSA-IONPs (125 µg/ml) for 24 h or treated with chlorpromazine (CPZ, 5 µg/ml), Amiloride (A, 1.6 µg/ml) or methyl-β-cyclodextrin (MβCD or M, 125 µg/ml) for 1 h prior to adding the DMSA-IONPs. After a 24 h incubation in the presence of the DMSA-IONPs and the inhibitors, the cells were recovered, and their iron content quantified by ICP-OES as described above.

To study iron content of the extracellular medium of untreated or DMSA-IONP-treated cells, medium was collected after treatment and analyzed for ICP-OES. MDA-MB-231 cells were left untreated or treated for 24 h with DMSA-IONPs. After treatment, extracellular medium was collected, centrifuged at 13,000 rpm (equal to 15,800 g) for 15 min, RT. After centrifugation, 1 ml of medium was digested with 1 ml of HCl 37% ON and diluted up to 10 ml with deionized water. Samples were quantified for iron by ICP-OES.

### Analysis of oxidative stress following DMSA-IONP treatment

The Dihydrorhodamine 23 (DHR) probe (Invitrogen D23806) was used to determine if DMSA-IONPs induce ROS production, analysing its fluorescence by flow cytometry. MDA-MB-231 cells were seeded in 12-well plates and after treatment with DMSA-IONPs (125 µg/ml) for 24 h, they were washed with PBS and incubated with DHR (1:20,000 in medium) for 30 min under cell culture conditions. The cells were then recovered by trypsinization, centrifuged and resuspended in PBS for flow cytometry analysis. Propidium iodide (PI) was added to each sample and the DHR fluorescence was analysed on a Cytomics FC500 cytometer using FL1 (ex/em = 488/525 nm) while PI was assessed using FL3 (ex/em = 488/620). For each sample, 50,000 events were acquired and the DHR fluorescence/ROS production by live cells was quantified with the FlowJo Software. In order to corroborate that NAC reduces ROS production, MDA-MB-231 cells were treated with NAC (5 mM) for 2 h prior to adding DMSA-IONPs for 24 h in the presence of NAC. After treatment, staining and recovery of cells as indicated above, DHR fluorescence was analysed by flow cytometry. Likewise, ROS production was assessed in SVEC4-10 cells seeded in 12-well plates after treatment with DMSA-IONPs (60–125 µg/ml) for 24 h. After treatment, the cells were stained and recovered as indicated above and DHR fluorescence was analysed by flow cytometry.

### MDA-MB-231 conditioned medium (CM)

Culture medium was recovered from MDA-MB-231 cells left untreated or treated with DMSA-IONPs for 24 h, by centrifugation at 13,000 rpm (equal to 15,800 g) for 15 min at RT and collecting the supernatant. This medium was mixed 1:1 with fresh medium (DMEM +10% FBS +1% non-essential amino acids, 2 mM L-Glutamine, 1 mM Sodium Pyruvate, 100 IU/ml Penicillin and 100 mg/ml streptomycin) in order to obtain MDA-MB-231 cell conditioned medium (CM) to treat SVEC4-10 cells. The CM obtained from untreated MDA-MB-231 cells was used as a Control (CM-Control).

To analyze the size distribution of particles, EV-like or MVB-like structures in the extracellular medium of untreated and DMSA-IONP-treated cells, either normal medium, CM-Control and CM-DMSA-IONPs was collected, centrifuged as stated above and analyzed by Dynamic Light Scattering (DLS) using a Zetasizer Nano ZS (Malvern) apparatus. The size of particles, EV-like structure and MVB-like structures was expressed as the average size of the particle size distribution histogram, whereas the quantity of particles, EV-like structures and MVB-like structures was expressed as the area under the curve (AUC) analyzed by GraphPad Prism version 10.1.1.

### Tumor cell migration, chemotaxis and invasion

MDA-MB-231 tumor cell migration was studied in wound closure assays and in transwell migration assays. Likewise, SVEC4-10 cell migration was also studied by wound closure assay, while chemotaxis towards MDA-MB-231 CM was assessed by the transwell migration assays. Besides, MDA-MB-231 cell invasion was assessed in transwell assays in the presence of extracellular matrix (ECM) Geltrex^™^ (A14132-02: Gibco).

#### Wound closure assay

MDA-MB-231 or SVEC4-10 cells were plated at high confluence in 6- or 12-well plates and the following day, the MDA-MB-231 cells were treated with DMSA-IONPs alone or after adding NAC (5 mM) for 2 h and maintaining the DMSA-IONPs in the presence of NAC. Likewise, SVEC4-10 cells were treated with DMSA-IONPs or with CM-Control or the CM of MDA-MB-231 cells treated with DMSA-IONPs for 24 h (CM-DMSA). In both cases, untreated cells were used as controls. After treatment, the wells were washed, a wound was made in the well using a fine tip, the well was again washed and fresh culture medium was added. The cells were visualized under a Leica DMI6000B inverted microscope and monitored every 30 min, taking pictures of at least 3 positions per well over the whole period. The relative cell invasion, the proportion of the wound that was closed and the cell front velocity (CFV) was calculated from the images taken over 24 h. The relative cell invasion or area covered during migration was calculated by evaluating the area of the wound each hour using Image J software and the proportion invaded was calculated according to the formula: Wound closure (%) = $\:\frac{{A}_{i}-{A}_{f}}{{A}_{i}}\:x\:100$, where A_i_ corresponds to initial area and A_f_ corresponds to area at each time point. By measuring the slope of the linear curve of wound closure (wound closure vs. time) and the distance or width of the wound at the initial time, we can calculate the CFV = $\:slope\:x\frac{Area\:of\:wound\:at\:t=0h}{width\:of\:wound\:at\:t=0h}$.

#### Transwell migration assays

For transwell migration and chemotaxis assays, MDA-MB-231 or SVEC4-10 cells were left untreated or treated for 24 h with DMSA-IONPs and then recovered by trypsinization. The cells (50,000) were seeded onto the membrane of the transwell insert (Corning, 8 μm) in 100 µl of low FBS culture medium (0.2% FBS/DMEM) and allowed to attach for 10 min at 37 °C and in 5% CO_2_. Subsequently, 600 µl of migration medium containing the chemoattractant was added to the bottom of the transwell insert, while 0.2% FBS/DMEM alone was used as a negative control and migration medium (10% FBS/DMEM) as a positive control. To study SVEC4-10 chemoattraction, CM-Control and CM-DMSA were used as the migration medium. Cells were left to migrate for 10 h and then those cells that had not migrated were carefully removed from the apical side of the transwell insert with a cotton-tipped applicator. The migrated cells on the basal side of the transwell insert membrane were fixed with 4% paraformaldehyde (PFA) for 15 min at RT and after fixation, the well was left to dry and the cells were stained with DAPI (1:200 in PBS) for 10 min at RT. The inserts were washed with PBS and observed under an inverted Leica DMI6000B microscope with a 20X objective to ensure a representative field of view, counting and analyzing the migrated cells with Image J software.

For transwell invasion assays, MDA-MB-231 cells were left untreated or treated for 24 h with DMSA-IONPs. The day after treatment, Geltrex (A14132-02: Gibco) was prepared in deionized water (2 mg/ml), and 60–100 µl of the diluted Geltrex was placed on the top of the transwell insert membrane (Corning, 8 μm) and allowed to solidify for 1 h at 37 °C. MDA-MB-231 cells (50,000 cells) recovered by trypsinization were seeded onto the membrane in 100 µl low FBS culture medium (0.2% FBS/DMEM) and allowed to attach for 10 min at 37 °C in 5% CO_2_. Subsequently, 600 µl of migration medium containing chemoattractant was added to the bottom of the transwell insert and the cells were left to migrate for 24 h. As a negative control 0.2% FBS/DMEM alone was used and 10% FBS/DMEM medium was used as a positive migration control. The cells that did not migrate were carefully removed from the apical side of the transwell membrane with a cotton-tipped applicator and the migrated cells on the basal side of the transwell membrane were fixed for 15 min at RT with 70% ethanol. After fixation, the well was dried and the cells stained for 10 min with DAPI (1:200 in PBS) at RT. The inserts were then washed with PBS and observed under an inverted Leica DMI6000B microscope with a 20X objective to ensure a representative field of view, counting and analyzing the migrated cells with Image J software.

### Spreading and attachment of cell spheroids exposed to DMSA-IONPs

The hanging drop method was used to generate MDA-MB-231 spheroids [48], depositing 20 µl droplets of culture medium containing 1,000 cells/droplet on the lid of a petri dish. The lid was inverted and placed on the top of the petri dish filled with PBS to obtain a humid atmosphere. Spheroids were obtained after a 96 h incubation under cell culture conditions. These cell aggregates or spheroids attach and spread by forming a cell monolayer that expands around the spheroid. To determine if DMSA-IONPs interfere with cell attachment, untreated MDA-MB-231 spheroids were plated in 48-well plates containing culture medium (control) or culture medium containing DMSA-IONPs (125 µg/ml, one spheroid per well). Spheroid attachment was observed ON under a Leica DMI6000B epifluorescence microscope equipped with a 5X objective and +1.6 lens or a 10X objective and an environment control system (37 °C and CO_2_). Pictures were taken every 30 min with a CCD camera and analyzed with Image J software. The contour of tumor-spheroids was analyzed at each time point and the spreading area was calculated to determine the capacity of the tumor spheroid to attach and spread in different media, and in the presence or absence of DMSA-IONPs. To determine if a 24 h exposure to DMSA-IONPs modulated the capacity of MDA-MB-231 spheroids to attach and spread, MDA-MB-231 were treated with DMSA-IONPs (125 µg/ml) for 24 h in a low-attachment plate, after which spheroids were recovered and placed ON in 48-well plates containing culture medium without DMSA-IONPs. Spheroid attachment and cell spreading was analyzed with Image J software. Experiments were performed twice and 10 spheroids per condition were evaluated each time.

### Immunofluorescence studies of the actin cytoskeleton, and of TOM20, Rab7, Lamp-2 and LC3B expression

MDA-MB-231 cells were seeded on coverslips in a 24-well plate and treated with DMSA-IONPs for 24 h. After treatment, the coverslips were washed, placed in a humid chamber and fixed with 4% PFA for 15 min. After fixation, the coverslips were washed with PBS, permeabilized with 0.1% Triton X-100 at RT for 10 min prior to blocking for 1 h with the DAKO Antibody Diluent with Background Reducing Components (S3022: Agilent Technologies). After blocking, the cells were probed ON at 4 °C in a humidified chamber with the primary antibodies diluted in DAKO Antibody Diluent. The following day the coverslips were washed and incubated for 1 h at RT with the corresponding secondary antibody also diluted in DAKO Antibody Diluent. After staining, the coverslips were washed with PBS, counterstained for 10 min at RT with DAPI diluted 1:500 in PBS and mounted with Fluoromount-G (0100-01: Southern Biotech).

To study exocytosis of late endosomes, lysosomes and autophagosomes, PM-bound Rab7, Lamp-2 and LC3B was assessed by immunofluorescence without permeabilization of the cells. The primary antibody to label mitochondria was the TOM20 antibody (1:500, ab283317: Abcam), the Lamp2 antibody was used to label lysosomes (1:200, sc-18822: Santa Cruz), the Rab7 antibody was used to label late endosomes (1:500, sc-376362: Santa Cruz) and autophagosomes were labeled with the LC3B antibody (1:250, ab192890: Abcam). The actin cytoskeleton was stained with iFluor 647 conjugated Phalloidin (ab176759: Abcam) for 90 min at RT. The cells were visualized on a multispectral confocal Leica Stellaris 5 or Leica TCS SP8 microscope using 63X/1.40 oil objective with a zoom factor of 3X or 4X. Images were analyzed with Image J Software.

Total actin was assessed through the fluorescence intensity phalloidin in at least 200 cells. Intensity heatmaps of the actin distribution were obtained with the EzColocalization plugin [49]. Lamellipodia were identified as a convex stretch of perpendicular actin staining at the peripheral edge of the cell visualized through phalloidin staining. The extension of the lamellipodia was assessed as the perimeter corresponding to lamellipodia normalized to the total perimeter of the cell [8, 50].

Cortical F-actin thickness was quantified from confocal images using line intensity profile analysis. Z-stacks were acquired under identical imaging settings for all conditions, and the equatorial plane of each cell was selected for analysis. Using ImageJ (Fiji), a line was drawn perpendicular to the plasma membrane, spanning from the extracellular space into the cytoplasm. Intensity profiles were obtained using the *Plot Profile* function without prior thresholding to preserve raw fluorescence values. The resulting intensity versus distance data were exported and fitted to a single Gaussian distribution using nonlinear regression in GraphPad Prism. Mean and SD values were extracted from gaussian fitting analysis and cortical thickness was calculated as the full width (raw data prior to gaussian fitting) and as the full width at half maximum (FWHM) of the fitted curve (FWHM = 2.355 × SD). For each cell, multiple lines were analyzed and averaged to obtain a single thickness value, and measurements were performed on at least 20 cells per condition.

The shape of the mitochondria and the proportion of cells containing spherical or elongated mitochondria was assessed in at least 200 cells per condition. To study the disposition of the mitochondria throughout the cell, orthogonal projections of the mitochondrial and actin channels were obtained with ImageJ software. In addition, intensity plots of both channels (green for mitochondria and red for actin) were obtained with ImageJ along a specific line and the intensity peaks were overlapped.

To visualize IONPs by confocal microscopy, the confocal reflectance microscopy (CRM) technique was used, in which the signal is generated by elastically backscattered light from the sample. In this method, the detector is aligned to collect backscattered light at the same wavelength as the incident laser. Furthermore, the detector is specifically configured to capture non-fluorescent, backscattered light, allowing label-free imaging of IONPs within cells.

### Invadosome and PM-bound Rab7, Lamp-2 and LC3B quantification

To count the invadosomes and PM-bound Rab7, Lamp-2 or LC3B, a previously described protocol was used with ImageJ software [51]. Briefly, an invadosome count was obtained from the actin channel and the PM-bound markers were counted in their respective channel. First, the image was converted to 8-bits and then the background signal was reduced by smoothing with the subtract background function (value of Rolling: 6). The contrast was increased with the gamma correction function (value: 0.8) and the convolve function (5 × 5 matrix with values −1 minus the central value of 30), and the image was then converted into a binary image (black and white), segmenting adjacent objects with the watershed function. The objects or elements of interest were counted with the analyze particle function, determining the size and characteristics that must be found. To define the invadosome, a size of 0.05–5.05 µm^2^ and a circularity of 0.8–1.8 was assigned. Likewise, the definition of PM-bound Rab7, Lamp-2 or LC3B, meant the size was changed to 0.05–2.05 µm^2^. The show: mask option was chosen to indicate the particles that have these characteristics, i.e. the invadosomes. All the objects or elements were counted in the region of interest (ROI) created manually for the whole cell area.

### Flow cytometry analysis of total F-actin and membrane-bound Lamp-1, Rab7 and LC3B

The iFluor 647 conjugated Phalloidin (ab176759: Abcam) probe was used to determine total F-actin by flow cytometry. Likewise, Alexa 647-CD107a antibody clone 1D4B (BioLegend, 121610), Rab7 antibody (sc-376362: Santa Cruz) and LC3B (ab192890: Abcam) were used to determine membrane-bound Lamp-1, Rab7 and LC3B, respectively.

MDA-MB-231 cells were seeded in 12-well plates and after treatment with DMSA-IONPs (125 µg/ml) for 24 h, cells were collected. For total F-actin determination, cells were fixed with PFA 2% for 10 min at 4 °C, washed twice with PBS and centrifuged at 1,300 rpm, 5 min, 4 °C. Pellet was resuspended in 0.1% Triton X-100 and permeabilized for 10 min at RT. After permeabilization, cells were stained with 1X iFluor 647 conjugated Phalloidin diluted in PBS for 30 min at RT. Following staining, cells were washed and resuspended in PBS for flow cytometry analysis. iFluor 647 conjugated Phalloidin fluorescence was analysed on a Cytomics Gallios cytometer using FL6 (ex/em = 650/665 nm). For each sample, 50,000 events were acquired, and total F-actin content was quantified with the FlowJo Software. Likewise, for membrane-bound Lamp-1, Rab7 and LC3B determination, cells were incubated without permeabilization or fixation with Alexa 647-CD107a antibody (Lamp-1), Rab7 antibody and LC3B antibody (1:100 in PBS) for 1 h at RT. After staining, for Rab7 determination, cells were incubated with Goat α mouse IgG1-PE (1:100) for 30 min at RT as a secondary antibody. Likewise, for LC3B determination, cells were incubated with Goat α rabbit IgG-Alexa 488 (H + L) (1:500) for 30 min at RT as secondary antibody. Following staining, cells were washed and resuspended in PBS for flow cytometry analysis. Fluorescence of Alexa 647 was analyzed on a Cytomics Gallios cytometer using FL5 (ex/em = 650/665 nm). Conversely, fluorescence of Alexa 488 and PE were analyzed on a Cytomics FC500 cytometer using FL1 (ex/em = 488/525 nm) and FL2 (ex/em = 488/575 nm), respectively. For each sample, 50,000 events were acquired, and membrane-bound Lamp-1 and Rab7 were quantified with the FlowJo Software.

### Protein carbonylation

To assess actin oxidation, the level of protein carbonylation was assessed with the protein carbonyl assay kit (ab178020: Abcam). MDA-MB-231 cells were plated in petri dishes and the day after, the cells were left untreated (negative control), treated with DMSA-IONPs (125 µg/ml) for 24 h or treated with H_2_O_2_ (10 mM) for 15 min (positive control). After treatment, cells were washed twice with PBS, recovered by gentle scraping, and centrifuged at 5,000 g for 5 min at 4 °C. Then, the cells were lysed in 50 µl of 1x Extraction Buffer on ice for 20 min, centrifuged at 18,000 g for 20 min and the supernatant was collected. The proteins were denatured and derivated following the manufacture’s protocol, denaturing 10 µl of protein (40 µg) with 10 µl of 12% SDS and incubating the proteins with 20 µl of 1X DNPH solution for 15 min at RT. After derivation, the solution was neutralized with 20 µl of neutralization solution and a total of 15 µg of derivated proteins was loaded onto a SDS-PAGE gel. Samples cannot be heated prior to loading. The proteins were transferred to 0.2 μm PVDF membranes and blocked for 1 h at RT with 10% milk Tris-buffered saline/1% Tween 20 (TBST). The membranes were probed ON at 4 °C with a primary anti-DNP antibody diluted 1:1000 in TBST/1% non-fat milk, and then with a secondary HRP antibody for 1 h at RT diluted 1:5,000 in TBST/1% non-fat milk. In addition, the membranes were probed for 1 h at RT with a primary antibody against β-actin (A5441: Sigma) diluted 1:1000 in TBST/1% non-fat milk that was detected for 1 h at RT with a secondary anti-mouse-HRP antibody (P0447: Dako) diluted 1:1,000 in TBST/1% non-fat milk. Antibody binding to the proteins was visualized with the SuperSignal^™^ West Femto Maximum Sensitivity Substrate (34094: ThermoScientific) and the protein band intensity was quantified with ImageJ software.

### Statistical analysis

All the data are presented as the mean ± standard deviation (SD) of different biological replicates (*n* = 3). Two-group comparisons were evaluated using an unpaired Student’s t-test, whereas experiments involving more than two groups were analyzed using one-way or two-way analysis of variance (ANOVA). Following ANOVA, Dunnett’s multiple comparisons test was used for comparisons against a control group, Tukey’s post hoc test was applied for all pairwise comparisons, and Sidák’s post hoc test was used to compare different groups in the two-way ANOVA. Assumptions of normality and homogeneity of variance were assessed prior to analysis. The area under the curve (AUC) for size distribution of extracellular medium was also analyzed. Values of *p* < 0.05 were considered statistically significant: *p < 0.05, **p < 0.01, ***p < 0.001, and ****p < 0.0001. GraphPad Prism version 10.1.1 was used for all the statistical analyses.

## Results

### DMSA-IONPs induce oxidative stress in cells of a metastatic breast cancer cell line

**Fig. 1:**
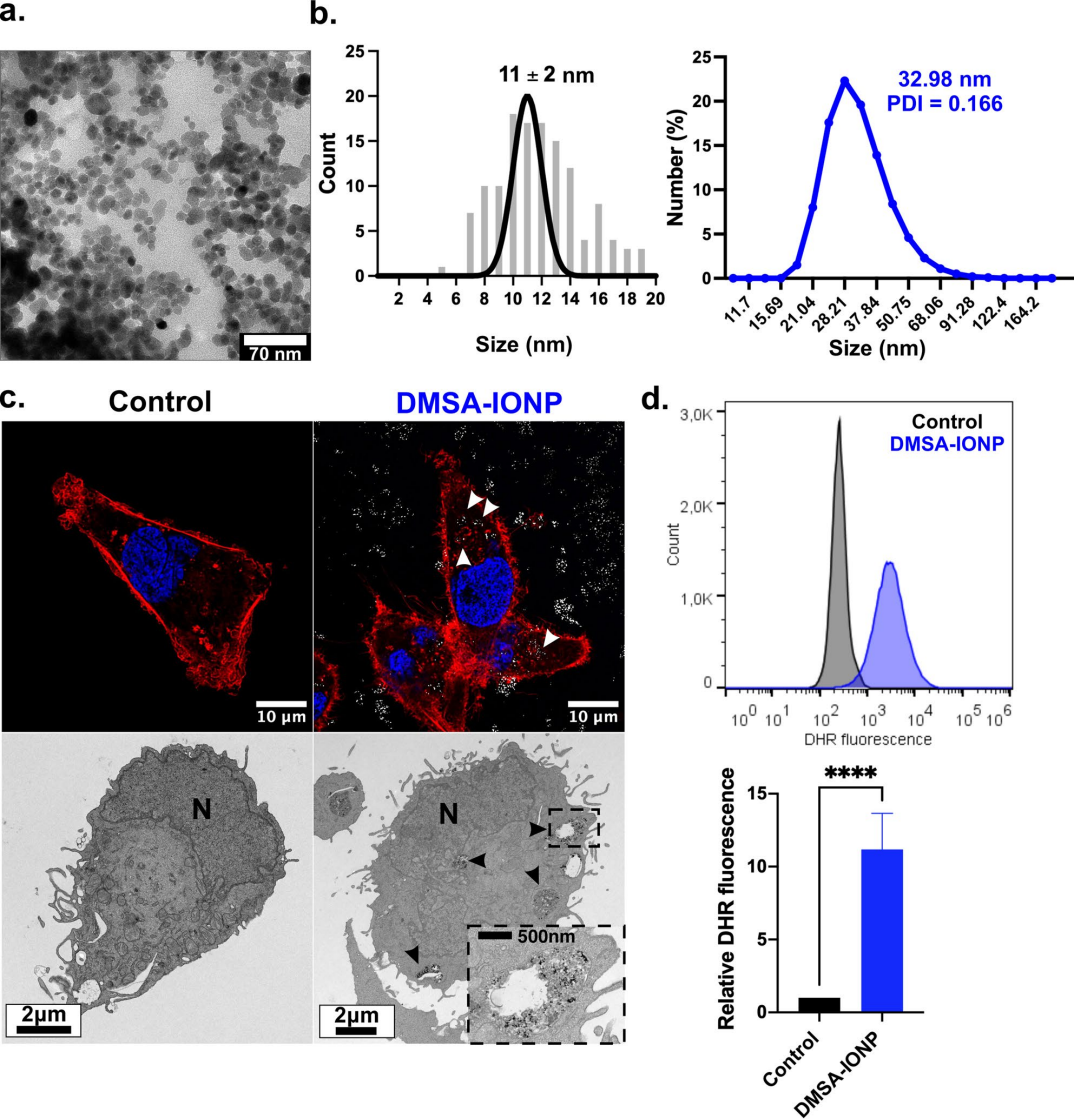
DMSA-IONP internalization and ROS production by MDA-MB-231 cells. **(a)** Transmission electron microscopy (TEM) of DMSA-coated IONPs. Scale bar = 70 nm. **(b)** Size distribution of the iron cores assessed by TEM and the hydrodynamic size of DMSA-IONPs assessed by DLS. **(c)** Intracellular localization of DMSA-IONPs in MDA-MB-231 cells. Upper panel: IONP internalization visualized by confocal microscopy with organelle markers: F-actin labelled with phalloidin, red; DAPI nuclear labelling, blue; IONPs visualized by reflection, white. Scale bar: 10 μm. Lower panel: Subcellular localization of IONPs visualized by TEM. N, Nucleus. Scale bar: 2 μm. The arrowheads indicate the DMSA-IONPs inside endosomal-like compartments and dashed square shows a zoomed-in region showing DMSA-IONPs localization within a membrane-surrounded vesicle (endosomal-like compartment), displayed in the bottom right corner). Scale bar for the zoomed area: 500 nm. **(d)** ROS production in MDA-MB-231 cells treated for 24 h with DMSA-IONPs: upper panel, DHR fluorescence histogram; lower panel quantification of DHR fluorescence. The data is the mean ±SD (*n* = 3) and an unpaired t-test was performed to evaluate the difference in ROS production between untreated and treated cells: *p < 0.05, **p < 0.01, ***p < 0.001, **** *p* < 0.0001

DMSA-IONPs have previously been characterized extensively [43, 52] and they are effective pro-oxidant tools as they induce the production of large amounts of ROS by a multitude of cell types [43]. Thus, we evaluated the potential of these DMSA-IONPs to influence the migration and invasion capacities of MDA-MB-231 BC cells. A new batch of DMSA-IONPs was synthesized here by a co-precipitation method, obtaining spherical iron cores (Fig. 1a) with an average core size of 11 nm (Fig. 1b, left). These iron cores were coated with DMSA, reaching a hydrodynamic size of 32.98 nm with a PDI of 0.166 (Fig. 1b, right). For NP-based pro-oxidant therapy to be effective, IONPs must be internalized and degraded in lysosomes to release iron ions, which can then react with oxidants like H_2_O_2_ to produce ROS. Therefore, the intracellular localization of these DMSA-IONPs and their capacity to produce ROS needs to be verified.

The internalization and localization of these DMSA-IONPs within endosome-like vesicles was assessed by confocal microscopy and TEM. Cells treated with DMSA-IONPs for 24 h were stained with phalloidin in order to visualize membrane actin or processed for TEM to assess their ultrastructure (Fig. 1c). DMSA-IONPs were taken up by MDA-MB-231 cells and they accumulated specifically in endosome-like vesicles resembling lysosomes (Fig. 1c). The ability of DMSA-IONPs to induce ROS production in these cells was assessed by flow cytometry using the DHR probe. DMSA-IONP internalization induced a 10-fold increase in ROS production relative to untreated cells. Thus, these DMSA-IONPs can be used in this cell line to assess their effects on prevent migration as a result of their pro-oxidant capacity.

### DMSA-IONP induced oxidative stress impairs breast cancer cell migration, invasion, spreading and attachment

**Fig. 2:**
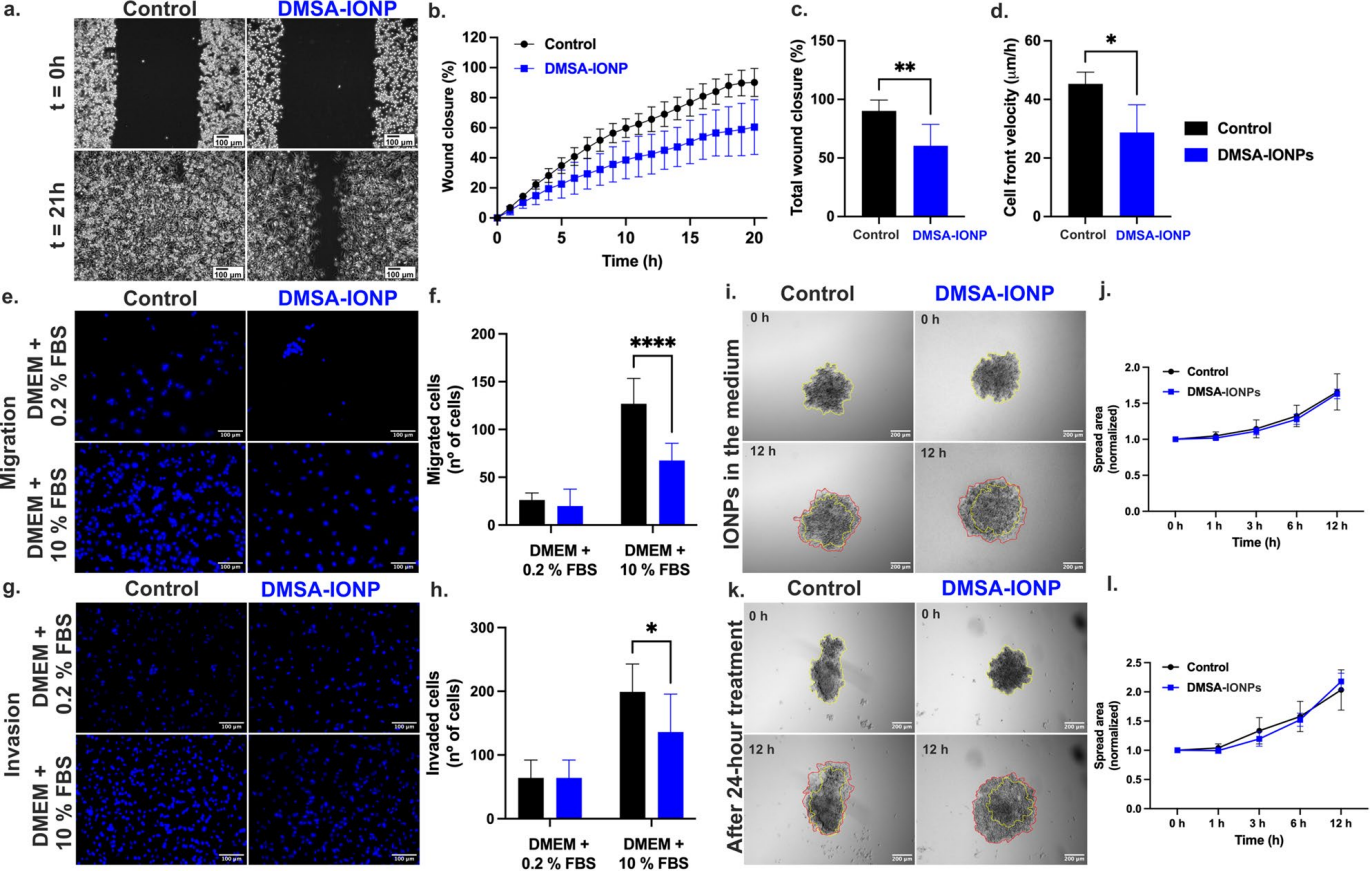
DMSA-IONPs affect MDA-MB-231 cell migration and their invasive capacity. **(a)** MDA-MB-231 cell migration evaluated in wound-healing assays. MDA-MB-231 cells were left untreated (control) or treated with DMSA-IONPs (125 µg/ml) for 24 h. Scale bar: 100 μm. **(b)** Relative wound closure over time. **(c)** Wound closure after 21 h. **(d)** Cell front velocity (CFV) over time. **(e)** Representative images of MDA-MB-231 cells migrated to the lower chamber in a transwell migration assay. **(f)** Number of cells migrated to the lower chamber. **(g)** Representative images of invasive MDA-MB-231 cells in the lower chamber of a transwell assay. **(h)** Number of invasive cells in the lower chamber. **(i)** Representative images of spheroid attachment and spreading in the presence or absence of DMSA-IONPs: spheroid area at initial time point, yellow; spheroid area at final time point, red. **(j)** Spreading of spheroids in the presence or absence of DMSA-IONPs. (**k)** Representative images of spheroid attachment and spreading after 24 h in the presence or absence of DMSA-IONPs: spheroid area at the initial time point, yellow; spheroid area at final time point, red. (**l)** Spreading of spheroids after 24 h in the presence or absence of DMSA-IONPs. The data represents the mean ±SD (*n* = 3 experimental replicates with 3–5 picture frames imaged for each condition). An unpaired t-test was used to assess the differences in wound-healing cell migration assay and two-way ANOVA followed by Sidák’s assay was used in transwell migration and invasion assays between untreated and DMSA-IONP treated cells: *p < 0.05, **p < 0.01, ***p < 0.001, ****p < 0.0001

The production of ROS, particularly hydrogen peroxide, may affect cell migration and adhesion [53]. Since DMSA-IONPs induce a ten-fold increase in ROS production in MDA-MB-231 cells (Fig. 1d), we wondered if this exacerbated production of ROS could somehow influence the redox balance, as well as the migratory, adhesive and invasive capacities of BC cells. MDA-MB-231 cell migration was evaluated in wound healing and transwell migration assays. For wound healing, MDA-MB-231 cells plated at high confluence were left untreated or exposed to DMSA-IONPs for 24 h and then, after making a wound in the cultures, cell migration was evaluated over 21 h (Fig. 2a-d). Treating MDA-MB-231 cells with DMSA-IONPs significantly reduced the rate of their migration (Fig. 2b) since untreated cells had completely closed the wound after 21 h (Fig. 2a and c), whereas those exposed to DMSA-IONPs only achieved 65% closure (Fig. 2c). The slower migration of DMSA-IONP-treated cells was reflected by a reduced CFV (Fig. 2d), as untreated cells migrated at a CFV of 45.3 μm/h whereas that of DMSA-IONP treated cells was 28.7 μm/h.

To elucidate whether the ROS produced somehow affected the migration of these cells, wound healing assays were performed in presence of the antioxidant NAC (Figure S1). The presence of NAC did not affect the internalization of DMSA-IONPs (Figure S1a) but it did reduce the production of ROS 3-fold (Figure S1b), such that the abrogation of ROS was not complete upon exposure to NAC. In the presence of NAC (5 mM), to which the cells were exposed for 2 h prior to treating with DMSA-IONPs and that was maintained during the 24 h treatment, MDA-MB-231 cells partially recovered their capacity to migrate relative to those exposed to DMSA-IONPs alone (Figure S1d). As such, after 21 h the cells exposed to DMSA-IONPs in the presence of NAC achieved 81% wound closure (Figure S1c and S1e). This partial recovery of wound closure coincided with a faster CFV, reaching 41 μm/h rate as opposed to cells treated with DMSA-IONPs alone (30 μm/h, Figure S1f).

The capacity of MDA-MB-231 cells to migrate was also evaluated in transwell migration assays, seeding 50,000 untreated cells or those treated for 24 h with DMSA-IONPs onto the upper transwell membrane. These cells in the top chamber of the transwell insert were left to migrate for 8 h, and then fixed and stained with DAPI to count migrated cells (Fig. 2e and f). DMSA-IONPs reduced the migration capacity of MDA-MB-231 cells through the transwell insert 2-fold relative to the untreated MDA-MB-231 cells (Fig. 2f). As a negative control the medium in the lower compartment was DMEM with 0.2% FBS, whereas culture medium with 10% FBS was used as a positive control.

MDA-MB-231 cell invasion capacity was also evaluated in a transwell invasion assay in which an ECM (Geltrex solution) was solidified on the upper surface of the transwell insert 1 h prior to seeding the MDA-MB-231 cells. After leaving the cells for 24 h to migrate and invade the ECM, the cells were DAPI stained for counting (Fig. 2g and h). DMSA-IONPs reduced the capacity of the MDA-MB-231 cells to invade the ECM by 1.5-fold (Fig. 2h). In addition, to the metastatic and migratory capacity of cells it is important to evaluate their spreading and attachment, which was evaluated with MDA-MB-231 tumor spheroids maintained for 24 h in the presence of DMSA-IONPs (Fig. 2i-l). DMSA-IONPs did not influence spheroid attachment or the spreading capacity of this cells, as there was similar spreading of untreated cells and those exposed to DMSA-IONPs (both in the medium or after incubating spheroids for 24 h with DMSA-IONPs: Fig. 2i-l). These results might be attributed to the irregular morphology and lack of compaction characteristic of these cell-type spheroids.

### DMSA-IONPs alter the actin cytoskeleton and the presence of actin-based structures involved in migration

Actin cytoskeleton polymerization and reorganization is required for cell migration and invasion, and in this sense, post-translational modifications of ABPs are fundamental to produce rapid cell responses. Oxidative stress can mediate the oxidation of sensitive cysteine or methionine residues in actin or adhesion molecules, such that it plays a role in regulating actin cytoskeleton dynamics [54]. As DMSA-IONPs induce ROS production in metastatic MDA-MB-231 BC cells (Fig. 1d) and they affect cell migration in a ROS-dependent manner (Fig. 2 and Figure S1), we evaluated the effects of IONPs on the actin cytoskeleton. We assessed whether DMSA-IONPs altered the abundance of actin structures associated with cell migration, such as lamellipodia, filopodia and invadosomes. Lamellipodia are actin-rich, wave-like PM structures that are usually thin (0.1–0.3 μm) and long (1–5 μm). These structures are protrusions where actin is densely packed in the form of branches and as they help cells attach to the migration matrix, they are located at the advancing front of the cell [55]. Filopodia are long, thin projections that project from the PM, containing linear arrangements of actin. They are usually finger-like in shape with a width range of 100–300 nm and their length can vary depending on the cell type and migratory conditions [56]. Finally, podosomes or invadopodia (invadosomes) are circular structures formed by actin that are important for migration, invasion and remodeling of the ECM as they are sites of matrix-cell contact. Invadosomes are dot-shaped and usually have a diameter of 0.5–1 μm [57].

**Fig. 3:**
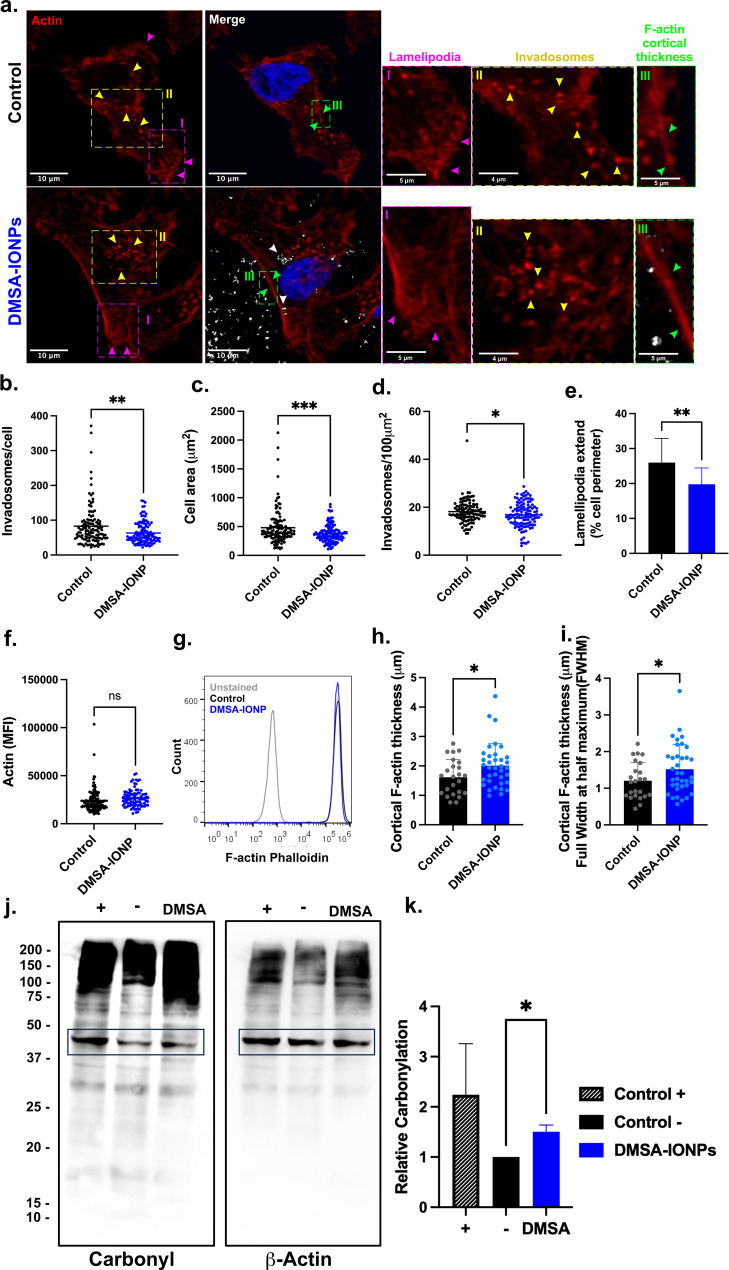
DMSA-IONP treatment affects the actin cytoskeleton in MDA-MB-231 cells.** (a)** Study of the actin cytoskeleton by immunofluorescence labelling with phalloidin in untreated (control) and DMSA-IONP-treated cells: actin stained with phalloidin, red; nuclei stained with DAPI, blue; DMSA-IONPs visualized by reflection, white/gray; invadosomes/podosomes, yellow arrowheads; lamellipodia, magenta arrowheads; cortical F-actin thickness, green arrowheads. The amplified areas correspond to the dashed boxes in yellow, magenta, and green showing invadosomas, lamellipodia and cortical F-actin thickness, repectively. Scale bar: 10 μm. **(b)** Quantification of the number of invadosomes in each cell. **(c)** Quantification of the cell area. **(d)** Quantification of the number of invadosomes normalized to the cell area. **(e)** Quantification of the extent of lamellipodia by evaluating the proportion of the cell perimeter that corresponds to lamellipodia. **(f)** Quantification of the mean fluorescence intensity (MFI) for actin per cell. **(g)** Quantification of total F-actin level per cell by flow cytometry. In grey unstained cells; in black control cells and in blue DMSA-IONP-treated cells. (**h-i)** Cortical F-actin thickness measured as total width (**h**) and full width at half maximum (FWHM) (**i**). (**j)** Determination of protein carbonylation in MDA-MB-231 cell lysates following DMSA-IONP treatment for 24 h or exposure to H_2_O_2_ (10 mM) for 15 min. Samples were derivated, resolved on SDS-PAGE gels and transferred to a PVDF membrane that was probed with a DNP antibody to detect carbonylation (left panel) and an antibody against β-actin (right panel). (**k)** The β-actin carbonyl signal intensity in the left panel was quantified and normalized to the total actin in the right panel by densitometry analysis by Image J from 3 experimental replicates (*n* = 3). The data represents the mean (± SD) of *n* = 200 cells for the invadosomes, cell area and actin MFI, and *n* = 20 cells for lamellipodia and cortical F-actin thickness. An unpaired t-test was used to analyze the differences in the number of invadosomes, cell area, actin MFI, lamellipodia, cortical F-actin thickness and β-actin carbonylation level between untreated and treated cells: *p < 0.05, **p < 0.01, ***p < 0.001, **** *p* < 0.0001

To study lamellipodia and invadosomes, the actin cytoskeleton in untreated MDA-MB-231 cells (control) or those treated with DMSA-IONPs for 24 h was stained with phalloidin. While the structure of DMSA-IONP treated cells appeared to be unaffected (Fig. 3a), these cells were smaller in area than untreated cells (Fig. 3c). There were fewer structures related to cell migration after DMSA-IONP treatment, fewer invadosomes (Fig. 3b and d) and less extensive lamellipodia (Fig. 3e). DMSA-IONPs did not significantly affect the total amount of actin (Fig. 3f and g), although actin was more abundant in regions of the cell membrane resembling lamellipodia in untreated cells and they accumulated in punctuate structures resembling invadosomes (Figure S2a). Moreover, cortical F-actin thickness was assessed in untreated and DMSA-IONP-treated cells by measuring both full width and full width at half medium (FWHM) from F-actin intensity profiles (Fig. 3h-i and Figure S2b). DMSA-IONP-treated cells exhibited a thicker cortical F-actin layer, with a full width of 2 μm compared with 1.6 μm in untreated cells (Fig. 3h) and a FWHM of 1.6 μm compared with 1.2 μm in untreated cells (Fig. 3i).

Oxidative stress can mediate actin or ABP oxidation, causing rearrangements to the actin cytoskeleton [15]. Higher levels of ROS can enhance the oxidation of amino acid side chains in actin, forming carbonyl groups and disrupting the cytoskeleton [58]. Subsequently, protein carbonylation was assessed in Western Blots of untreated and DMSA-IONP treated cells to assess whether this treatment modulated protein oxidation (Fig. 3j). DMSA-IONPs increased the carbonylated proteins detected relative to untreated cells as the intensity of higher molecular weight proteins increased following DMSA-IONP treatment (Fig. 3k). Indeed, a band appeared at a molecular weight resembling that of β-actin, between 50 and 37 kDa that was intensely carbonylated after DMSA-IONP treatment relative to untreated cells (as well as in the positive controls), and this intensely carbonylated band was recognized when the membrane was probed for β-actin (Fig. 3j). When quantified, β-actin carbonylation increased mildly upon DMSA-IONP treatment (Fig. 3k) and thus, DMSA-IONPs did appear to induce the oxidation of β-actin in MDA-MB-231 cells.

Integrins are heterodimeric cell surface receptors that play a pivotal role in cell adhesion and migration. In particular, expression of the α_v_β_3_ integrin is upregulated in endothelial cells and BC cells that undergo angiogenesis and metastasis [59, 60], a protein linked to focal adhesions and cell motility [61]. As DMSA-IONPs affected both cell migration and adhesion, we wondered if integrin expression might also be affected somehow. However, when we evaluated the membrane-bound expression of the α_v_β_3_ integrin no differences were observed between untreated and DMSA-IONP treated cells (Figure S3). Oxidative stress can also induce integrin oxidation and redox modifications of integrin α_v_β_3_ in turn induce protein conformational changes, affecting their function [62]. As the protein carbonylation induced by DMSA-IONPs is increased in high-molecular-weight proteins (Fig. 3j), this may indicate that high molecular weight proteins such as integrin α_v_β_3_ could also be oxidized, as its molecular weight is 140 kDa. Therefore, while DMSA-IONPs did not affect the amount of integrin or actin protein in the cells, the potential oxidation of these proteins and the effects on the actin cytoskeleton are likely to influence the formation of migratory structures like invadosomes, focal adhesions and lamellipodia.

### The spatial distribution of mitochondria is not affected by DMSA-IONPs

**Fig. 4:**
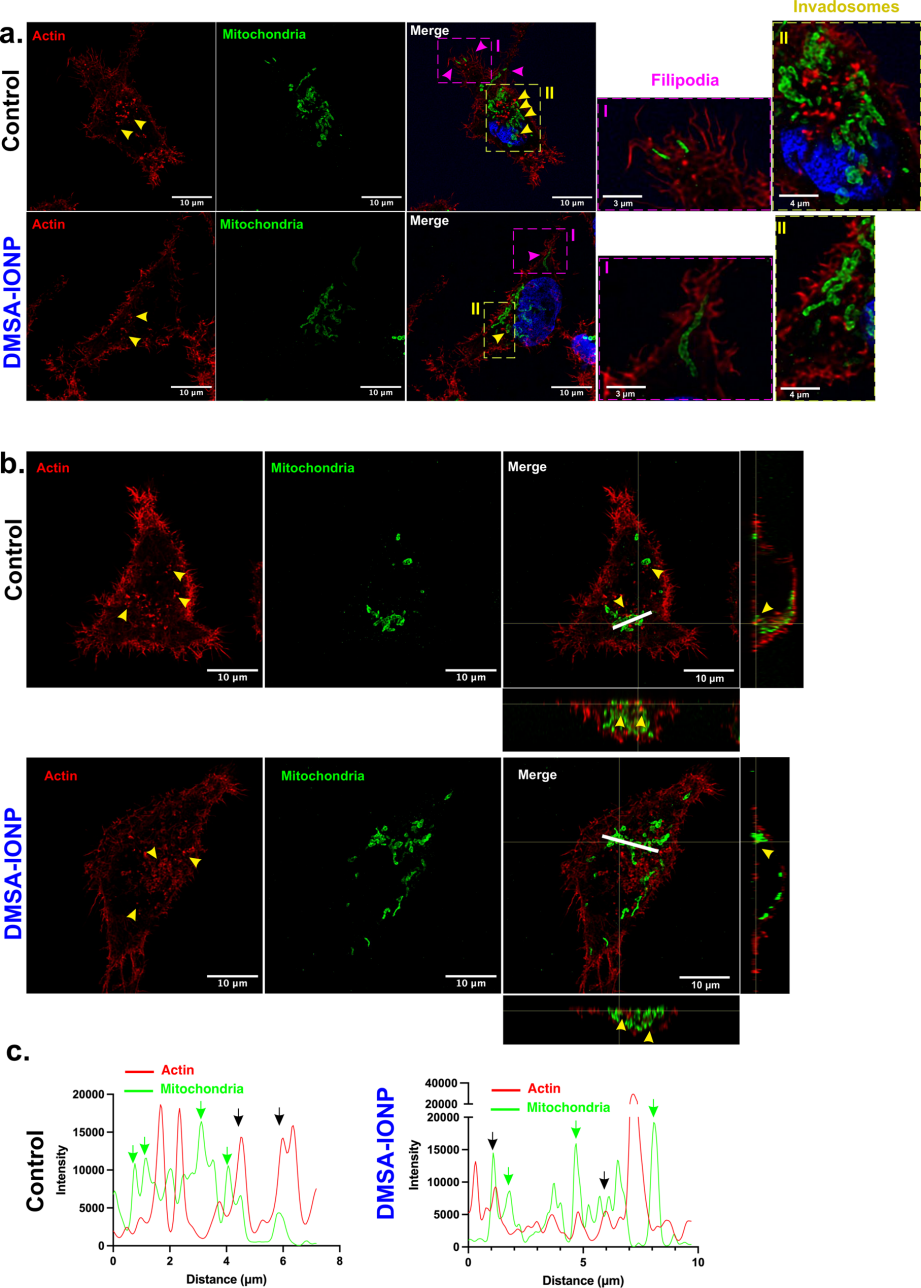
The localization of mitochondria around actin structures related to migration. **(a)** Localization and disposition of mitochondria in the cell visualized by immunofluorescence. Yellow arrowheads indicate invadosomes and the magenta arrowheads indicate mitochondria migrating towards migration structures such as filipodia. The amplified area I, highlighted in magenta, corresponds to the dashed box in magenta exemplifying mitochondria in filopodia (control cells) or migrating towards the edge of cell (DMSA-IONP-treated cells). The amplified area II, highlighted in yellow, shows mitochondria positioned close to or surrounding invadosomes. **(b)** Localization of mitochondria around invadosomes and in the volume of cell: actin labelled with phalloidin, red; mitochondria labelled with the TOM20 antibody, green; nuclei stained with DAPI, blue. Scale bar: 10 μm. **(c)** Study of the red and green pixel intensities along the line drawn in (b): the red line corresponds to the actin intensity and the green line corresponds to that of mitochondria; the black arrows indicate co-localization of invadosomes and mitochondria; the green arrows indicate mitochondria surrounding invadosomes

Alterations to the spatial distribution of mitochondria affects migration and reduces the stability of focal adhesions [9, 63]. We previously showed that DMSA-IONPs alter the disposition of mitochondria in MDA-MB-231 cells, shifting from a spherical towards an elongated shape (Figure S4) [43]. As mitochondria adopt a more elongated phenotype, we hypothesized that mitochondrial transport towards the edge of the cell might be affected and hence, we assessed the effect of DMSA-IONPs on the distribution of mitochondria in MDA-MB-231 cells. Mitochondria adopted a perinuclear position in both treated and untreated MDA-MB-231 cells, close to the nucleus and in some cases, isolated mitochondria could be seen in migration related structures (near lamellipodia or filopodia) or migrating towards them (Fig. 4a). In untreated cells, the mitochondria appear more dispersed that in those exposed to DMSA-IONPs and they appear to localize in filopodia, and shifting towards migration related structures. Likewise, although the mitochondria of DMSA-IONP treated cells appeared to be more elongated, they also appeared to be migrating towards the edge of the cell. Therefore, there is no apparent difference between the distribution of mitochondria in treated and untreated cells.

The localization of mitochondria close to focal adhesions or invadosomes is important to ensure an adequate energy supply to these structures and for actin cytoskeleton rearrangements [64]. Thus, the accumulation of mitochondria in the basal part of the cell is important for them to interact with the actin cytoskeleton. This basal localization of mitochondria was corroborated in orthogonal XZ and YZ planes of untreated and DMSA-IONP treated cells (Fig. 4b). In both conditions mitochondria were located basally and close to invadosome-like structures (punctuate actin structures). Co-localization of mitochondria with invadosomes was studied in intensity graphs of the actin and mitochondrial channels along the white line (Fig. 4b and c). Mitochondria appeared to surround and co-localize with invadosomes (green and black arrows, respectively) in both conditions. Thus, DMSA-IONPs do not appear to affect the distribution of mitochondria at the periphery of the cell, nor around invadosomes.

### DMSA-IONPs localize inside the endo-lysosomal system structures

**Fig. 5:**
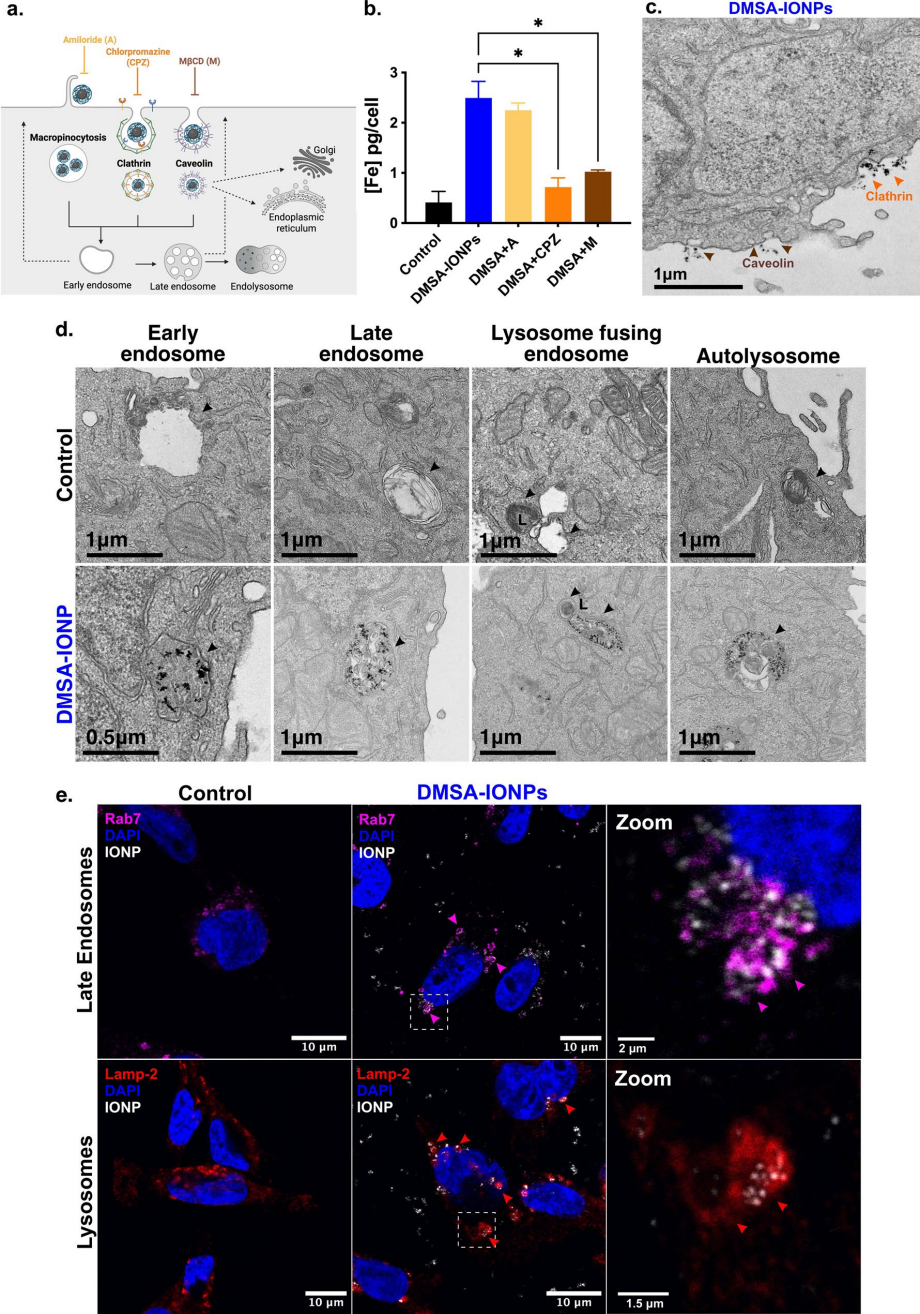
DMSA-IONP endocytosis and intracellular localization in endo-lysosomal structures of MDA-MB-231 cells. **(a)** DMSA-IONPs are internalized by different mechanisms, as assessed using inhibitors for the main endocytotic pathways: amiloride for macropinocytosis, chlorpromazine for clathrin-dependent endocytosis and methyl-β-cyclodextrin for caveolin-dependent endocytosis. **(b)** Measurement of DMSA-IONP internalization by ICP-OES in the presence or absence of inhibitors of endocytosis. **(c)** Representative TEM image of DMSA-IONP endocytosis in clathrin and caveolin-like structures in MDA-MB-231 cells treated for 3 h. **(d)** Representative TEM images of early endosomes, late endosomes, lysosomes fusing with endosomes and autolysosomes in untreated and DMSA-IONP treated cells (3 h and 24 h). **(e)** The immunofluorescent localization of DMSA-IONPs in late endosomes (Rab7^+^ endosomes) and lysosomes (Lamp-2^+^ endosomes): late endosomes labelled with the Rab7 antibody, magenta; lysosomes labelled with the Lamp-2 antibody, red; nuclei stained with DAPI, blue; DMSA-IONPs visualized by reflection, white/grey. The amplified area corresponds to the dashed box in the middle panel showing endosomes and lysosomes containing DMSA-IONPs. Scale bar: 10 μm. The data is the mean ± SD (*n* = 3 experimental replicates). One-way ANOVA with Dunnett’s multiple comparison test was used to analyze the differences between DMSA-IONP-treated cells alone and the treatment of DMSA-IONPs with the different inhibitors: *p < 0.05, **p < 0.01, ***p < 0.001, **** *p* < 0.0001

IONP uptake by cells is driven by different endocytotic mechanisms depending on their physicochemical characteristics. Depending on their means of entry, IONPs may be internalized via membrane invaginations that form endocytotic vesicles directed to early endosomes. From here, the IONPs internalized can be either recycled to the PM or trafficked towards late endosomes, finally fusing with lysosomes for their degradation [65]. The main endocytic pathways are phagocytosis, macropinocytosis, clathrin-dependent endocytosis, caveolin-dependent endocytosis and clathrin- and caveolin-independent endocytosis. To determine how DMSA-IONPs were internalized, their uptake was assessed in the presence of different inhibitors: A, an inhibitor of macropinocytosis; CPZ, an inhibitor of clathrin-dependent endocytosis; and MβCD or M, an inhibitor of caveolin-dependent endocytosis. As such, MDA-MB-231 were left untreated, treated with DMSA-IONPs alone or exposed to the different inhibitors for 1 h, and then treated for 24 h with DMSA-IONPs. After treatment, their iron content was assessed by ICP-OES to determine the extent of DMSA-IONP internalization (Fig. 5b). The intracellular concentration of DMSA-IONPs inside MDA-MB-231 cells was around 3 pgFe/cell, yet the iron concentration decreased 2-fold when cells were exposed to clathrin (CPZ) or caveolin inhibitors (M) indicating that DMSA-IONPs were taken up by clathrin- and caveolin-dependent endocytosis (Fig. 5b). By contrast, the macropinocytosis inhibitor (A) had no effect on the iron content of MDA-MB-231 cells treated with DMSA-IONPs (Fig. 5b). TEM was used to corroborate this mechanism of entry in MDA-MB-231 cells and visualize clathrin- and caveolin-like structures (Fig. 5c). Clathrin-like structures were evident as local membrane invaginations forming small buds [66], larger than caveolin-like structures that have a more bulb or flask shape (Fig. 5c: [67].

The different mechanisms of internalization can dictate whether IONPs accumulate in acidic or non-acidic vesicles, as clathrin-dependent endocytosis generally leads towards endo-lysosomal compartments [68, 69], whereas caveolin-dependent endocytosis can evade autolysosomes and hence, IONP degradation [70]. To determine if DMSA-IONPs enter endo-lysosomal structures in MDA-MB-231 cells, intracellular location was studied by TEM after a 3 and 24 h treatment (Fig. 5d). Early endosomes are round membranous structures with low density staining, whereas late endosomes appear darker and with the presence of intraluminal structures (Fig. 5d left and middle-left panel: [71]. Lysosomes have highly organized inner folds of their membrane, with a darker and more consistent appearance than multilamellar bodies (MLBs, Fig. 5d, middle-right panel showed with L: [72]). Fusion of lysosomes with autophagosomes or endosomes generates autolysosomes (Fig. 5d right panel: [73]). DMSA-IONPs were evident in all types of endo-lysosomal structures in MDA-MB-231 cells, in early endosomes, late endosomes and autolysosomes (Fig. 5d). Precise identification based solely on TEM imaging may be inconsistent and thus, the presence of DMSA-IONPs inside late endosomes and lysosomes was confirmed by confocal microscopy (Fig. 5e) in cells labelled with an antibody against Rab7 to specifically detect the former and an antibody against Lamp-2 to detect the latter. DMSA-IONPs clearly accumulated in late endosomes and lysosomes (Fig. 5e), and their presence in acidified vesicles was further corroborated by lysotracker green staining and flow cytometry (Figure S5). Indeed, the presence of DMSA-IONPs in autolysosomes is consistent with the strong ROS production by this cell line (Fig. 1d).

### DMSA-IONPs can modulate the unconventional pathways for protein secretion by modulating endosome, lysosome and autophagosome exocytosis

**Fig. 6:**
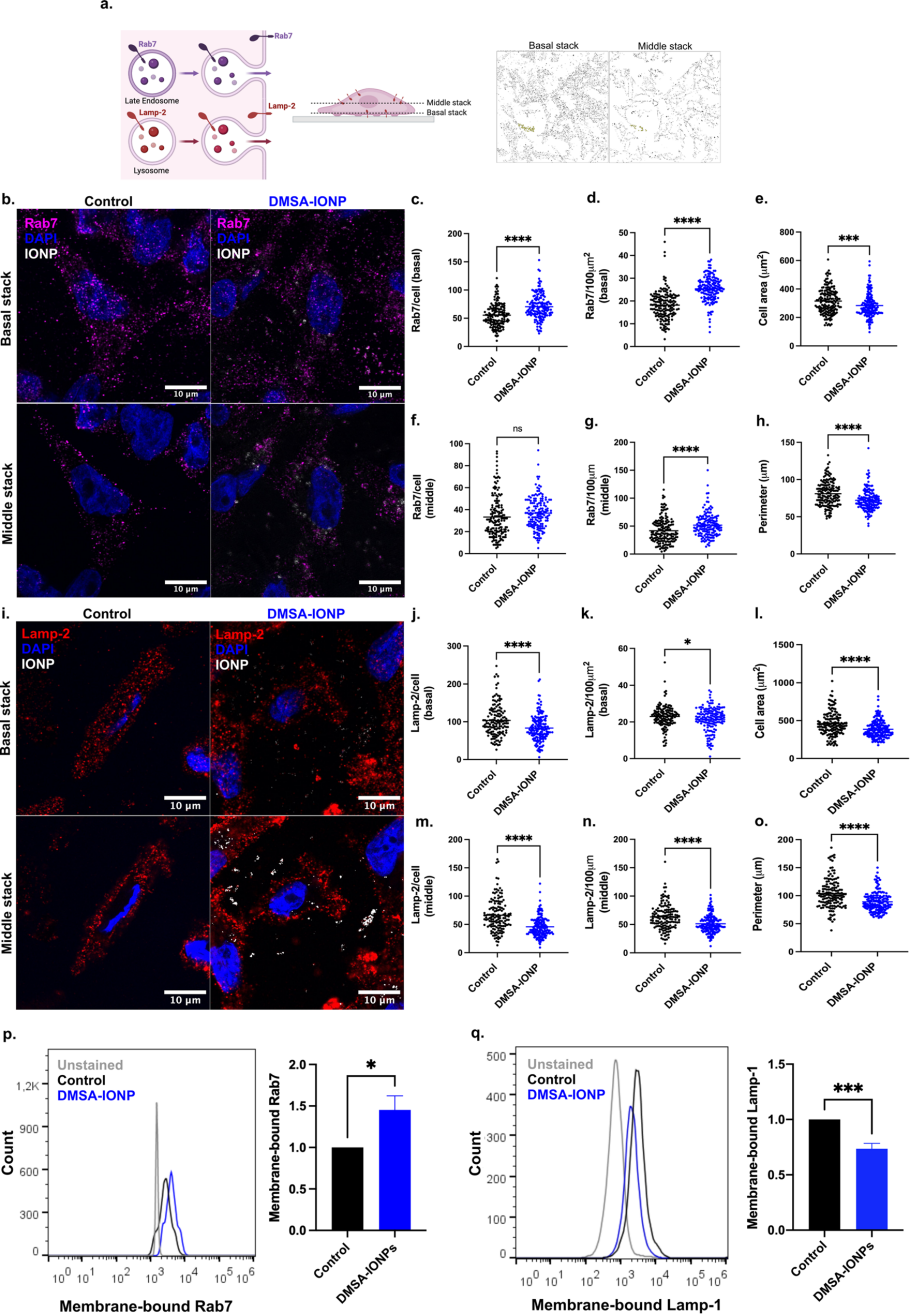
DMSA-IONPs affect the anterograde transport of late endosomes and lysosomes to the cell periphery. **(a)** Analysis of the exocytosis of late endosomes (Rab7^+^) and lysosomes (Lamp-2^+^) by immunofluorescence of non-permeabilized cells, assessing the Rab7 and Lamp-2 at the cell membrane. Analysis of the middle and the basal/ventral stack. **(b)** Images of Rab7 at the cell membrane of untreated (control) and DMSA-IONP treated cells: Rab7, magenta; DAPI stained nuclei, blue; DMSA-IONPs visualized by reflection, white/grey. The upper panel corresponds to the basal stack and the lower panel to the middle stack. Scale bar: 10 μm. **(c)** Analysis of Rab7^+^ puncta in each cell, corresponding to the basal stack **(d)** Analysis of Rab7^+^ puncta in each cell normalized to the cell area in the basal stack **(e)** Cell area **(f)** Analysis of the Rab7^+^ puncta in each cell corresponding to the middle stack **(g)** Analysis of Rab7^+^ puncta in each cell normalized to the cell perimeter in the middle stack **(h)** Cell perimeter **(i)** Images of the Lamp-2^+^ puncta at the cell membrane in untreated (control) and DMSA-IONP treated cells: Lamp-2, red; DAPI stained nuclei, blue; DMSA-IONPs visualized by reflection, white/grey. The upper panel corresponds to the basal stack and the lower panel to the middle stack. Scale bar: 10 μm. **(j)** Analysis of Lamp-2^+^ puncta in each cell corresponding to the basal stack. **(k)** Analysis of Lamp-2^+^ puncta in each cell normalized to the cell area in the basal stack. **(l)** Cell area. **(m)** Analysis of Lamp-2^+^ puncta in each cell corresponding to the middle stack. **(n)** Analysis of Lamp-2^+^ puncta in each cell normalized to the cell perimeter in the middle stack. **(o)** Cell perimeter. **(p)** Membrane-bound Rab7 assessed by flow cytometry. Left panel: Rab7 fluorescence histogram; Right panel: Quantification of Rab7 fluorescence. **(q)** Membrane-bound Lamp-1 assessed by flow cytometry. Left panel: Lamp-1 fluorescence histogram; Right panel: Quantification of Lamp-1 fluorescence The data is the mean (± SD) of *n* = 200 cells of 3 experimental replicates. An unpaired t-test was used to analyze the differences between untreated and treated cells: *p < 0.05, **p < 0.01, ***p < 0.001, **** *p* < 0.0001

The CPS involves direct transport of a translated protein from the ER towards the Golgi apparatus, followed by extracellular secretion [35]. By contrast, the UCPS bypasses the ER-Golgi network by secreting proteins through late endosomes/MVBs, lysosomes and autophagosomes [74]. Late endosomes or MVBs contain multiple intraluminal vesicles and they can fuse directly with lysosomes to form autolysosomes, structures that promote the degradation of their intraluminal content or direct it to the PM to be secreted [37]. This MVB secretion has been associated with the generation of EVs and their exocytosis [37, 74]. In addition, lysosomes can also fuse with the PM to participate in the lysosomal secretory pathway [75]. Considering that DMSA-IONPs accumulate inside endo-lysosomal structures, early and late endosomes and autolysosomes (Fig. 5d and e), we evaluated if this intracellular localization somehow affected the exocytosis of these vesicles. When MVBs and lysosomes fuse with the PM, markers like Rab7 (late endosome) and Lamp-2 (lysosome) become exposed, such that the detection of these markers in non-permeabilized cells is a good readout of endosome exocytosis [76]. We studied the distribution of these markers at the PM of untreated and DMSA-IONP treated cells by immunofluorescence without permeabilization, analyzing the basal stack where cells are attached to the coverslip, and the middle stack. The number of Rab7- and Lamp-2 puncta was quantified and normalized to the cell area or cell perimeter (Fig. 6). DMSA-IONPs increased the absolute number of Rab7 puncta in the basal domain (Fig. 6c), whereas no significant changes were observed in the absolute number of Rab7 puncta in the middle region (Fig. 6f). As DMSA-IONPs affect the actin cytoskeleton (Fig. 3), the cell area and perimeter were reduced by this treatment (Figs. 3c and 6e and h), such that the number of Rab7 puncta at the PM was normalized to the cell area and perimeter, in both cases being reduced (Fig. 6d and g). When the presence and number of Lamp-2 puncta at the PM was studied (Fig. 6i), both the absolute and the normalized values of Lamp-2 puncta in the basal and middle regions were reduced by DMSA-IONPs (Fig. 6j and o).

Furthermore, membrane-bound markers of late endosomes and lysosomes were also quantified by flow cytometry in non-permeabilized cells (Fig. 6p-q). Membrane-bound Rab7, a marker of late endosomes or MVBs, was approximately 40% more abundant in DMSA-IONP-treated cells compared to untreated control cells (Fig. 6p). Conversely, membrane-bound Lamp-1, a lysosomal marker, was reduced by up to 30% in DMSA-IONP-treated cells compared to control cells (Fig. 6q).

Considering that the fusion of late endosomes or MVBs with the PM can induce the exocytosis of EVs or exosomes [77], we evaluated the presence of MVB-like or EV-like structures in untreated and DMSA-IONP treated cells by TEM (Figure S6a). In both conditions extracellular MVB-like or EV-like structures were evident but their content, or the type of structure released, was not characterized further. In addition, the size distribution of MVB-like or EV-like structures in the extracellular medium of untreated and DMSA-IONP-treated MDA-MB-231 cells was measured (Figure S6b). The extracellular medium of DMSA-IONP-treated MDA-MB-231 cells contained a greater number of EV-like structures, as reflected by a broader size-distribution histogram and a two-fold increase in the area under the curve (AUC) compared to normal medium or extracellular medium from untreated MDA-MB-231 cells (Figure S6b). The sizes of EV-like structures also differed among conditions: normal medium and CM from untreated MDA-MB-231 cells (CM-Control) contained EV-like structures of approximately 30 nm for normal medium and of approximately 37 nm for CM-Control, whereas structures in the extracellular medium from DMSA-IONP-treated cells (CM-DMSA-IONPs) displayed larger sizes, averaging around 83 nm (Figure S6b). However, DMSA-IONPs decrease the Lamp-2 marker at the PM, indicating that they diminish the extracellular release of lysosomal content. As lysosomes have an acidic pH and produce ROS, we assessed the changes in pH or ROS production in the medium of untreated or DMSA-IONP treated cells (Figure S7), showing that DMSA-IONPs reduced the amount of extracellular ROS (Figure S7a) with no change in pH (Figure S7b and S7c).

**Fig. 7:**
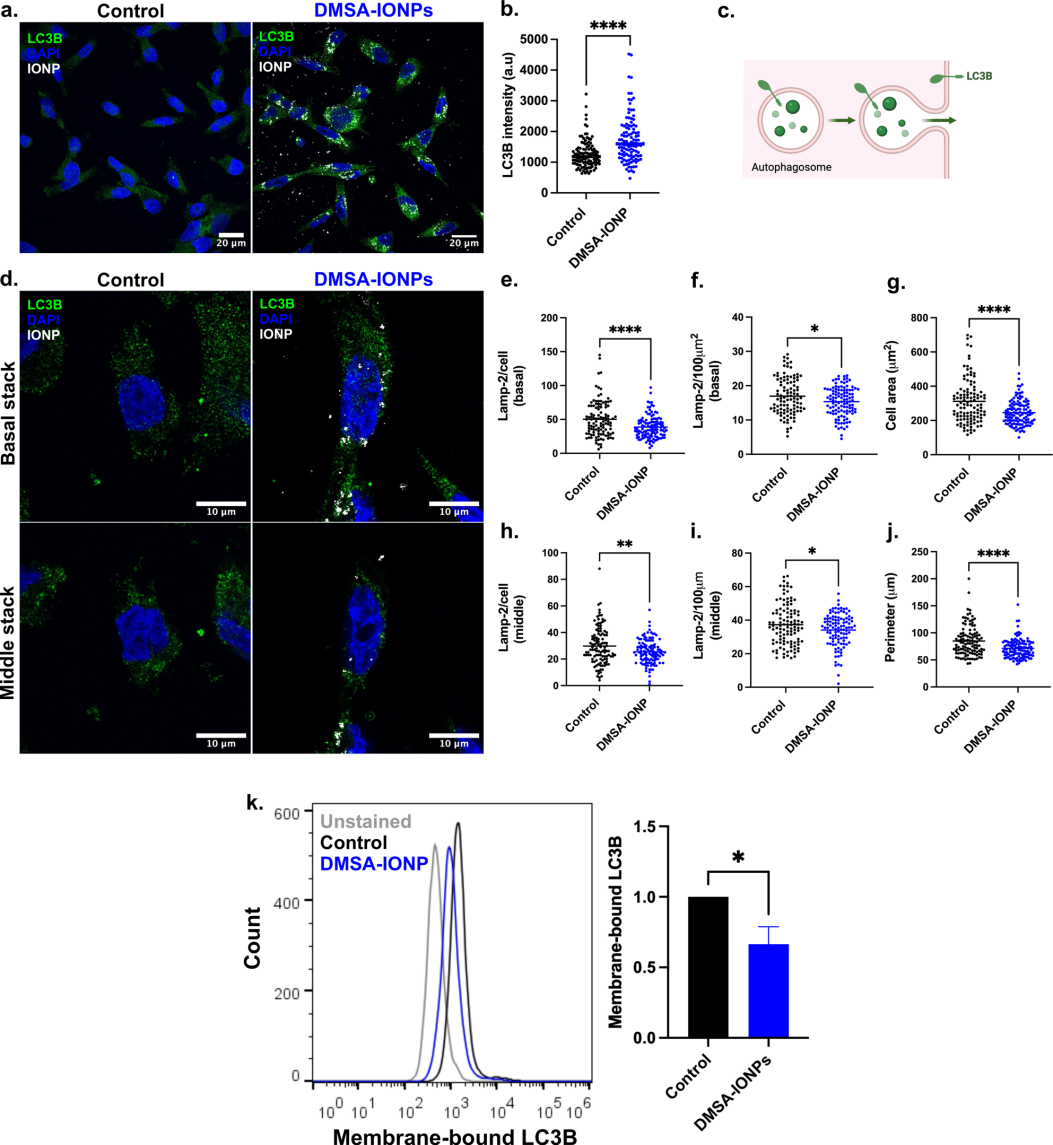
DMSA-IONP affects autophagy and secretory autophagy in MDA-MB-231 cells.** (a)** The induction of autophagy in untreated and DMSA-IONP treated cells as witnessed through LC3B immunofluorescence: LC3B staining, green; DAPI nuclear staining, blue; DMSA-IONPs visualized by reflection, white/grey. Scale bar: 20 μm. **(b)** Quantification of LC3B intensity as a read-out of autophagy. **(c)** Secretory autophagy assessed through the LC3B immunofluorescence at the membrane of non-permeabilized cells. **(d)** Images of LC3B staining at the cell membrane of untreated (control) and DMSA-IONP treated cells: LC3B, green; DAPI nuclear staining, blue; DMSA-IONPs visualized by reflection, white/grey. The upper panel corresponds to the basal stack and the lower panel to the middle stack. Scale bar: 10 μm. **(e)** Analysis of LC3B^+^ puncta in each cell corresponding to the basal stack **(f)** LC3B^+^ puncta in each cell normalized to the cell area in the basal stack. **(g)** Cell area. **(h)** LC3B^+^ puncta in each cell of the middle stack. **(i)** LC3B^+^ puncta in each cell normalized to the cell perimeter in the middle stack. **(j)** Cell perimeter. **(k)** Membrane-bound LC3B assessed by flow cytometry. Left panel: LC3B fluorescence histogram; Right panel: Quantification of LC3B fluorescence. The data is the mean (± SD) of *n* = 200 cells of 3 experimental replicates. An unpaired t-test was used to assess the differences in LC3B labelling, cell area and cell perimeter: *p < 0.05, **p < 0.01, ***p < 0.001, **** *p* < 0.0001

Autophagy is the process by which intracellular elements that may be damaged and harmful are recycled or degraded [78]. This process involves the formation of an autophagosome that engulfs the cytoplasmic material in a double membrane structure, which subsequently fuses with a lysosome to form an autolysosome that degrades its components [73]. If autophagosomes do not fuse with lysosomes they can fuse with the PM, thereby releasing their material extracellularly in a process known as secretory autophagy [36]. Secretory autophagy releases several mediators into the tumor microenvironment, participating in the intercellular communication between cancer and stroma cells [79]. In the light of this role in intercellular communication, we assessed if DMSA-IONPs modified degradative or secretory autophagy in MDA-MB-231 BC cells. To evaluate degradative autophagy, permeabilized untreated and DMSA-IONP treated MDA-MB-231 cells were stained by immunofluorescence with the autophagosome marker LC3B (Fig. 7a and b), and DMSA-IONPs were seen to induce an increase in LC3B that reflects enhanced degradative autophagy (Fig. 7a and b). To study secretory autophagy, LC3B was evaluated in these cells without permeabilization, assessing the presence of LC3B at the PM and normalizing this to the cell area and perimeter (Fig. 7c and d). In both the basal stack where cells attach to the coverslip, and in the middle stack, the absolute and the normalized values for LC3B staining were reduced by DMSA-IONPs (Fig. 7d, e, f, h and i). Concomitant with these effects, DMSA-IONP treatment reduced the area and perimeter of the cells (Fig. 7g and j). Furthermore, membrane-bound LC3B was also analyzed by flow cytometry (Fig. 7k), showing a 35% reduction in DMSA-IONP-treated cells compared to untreated control cells (Fig. 7k). As less Lamp-2 and LC3B were evident at the PM of cells exposed to DMSA-IONPs (Figs. 6 and 7), these NPs affect both the lysosomal secretory pathway and secretory autophagy in these MDA-MB-231 BC cells. Given the possible role of secretory autophagy in intercellular communication, this effect of the DMSA-IONPs could influence paracrine communication.

### DMSA-IONPs affect endothelial cell migration and cell proliferation

Secretory autophagy and lysosome secretion may be involved in the communication between cancer cells and their microenvironment [80, 81], such that modulating these processes might represent a novel therapeutic strategy to inhibit cancer cell progression [82, 83]. DMSA-IONPs affect the distribution of lysosome and autophagosome markers at the PM, provoking a strong reduction in lysosome and autophagosome exocytosis that could alter communication through this channel. As DMSA-IONPs produce certain anti-metastatic effects, limiting MDA-MB-231 cell migration, spreading and invasion, we evaluated if they somehow modulate the migration capacity and chemoattraction of endothelial cells. Hence, we studied the effects of medium conditioned (CM) by untreated or DMSA-IONP treated MDA-MB-231 cells on endothelial SVEC4-10 cells. The supernatant of MDA-MB-231 cells cultured in the presence or absence of DMSA-IONPs (125 µg/ml, 24 h) was recovered, centrifuged to remove cellular debris and DMSA-IONPs, and mixed 1:1 with fresh culture medium to obtain CM-Control and CM-DMSA-IONPs, respectively. As exocytosis of MVB- or EV-like structures is enhanced by DMSA-IONPs (Fig. 6b-h and Figure S6), these cells may liberate DMSA-IONPs that do not reach the lysosomes for degradation into their extracellular milieu. To determine whether conditioned medium (CM) from MDA-MB-231 cells contained DMSA-IONPs after centrifugation and prior to endothelial cells treatment, the iron content of non-centrifuged and centrifuged CM-Control and CM-DMSA-IONP samples was measured by ICP-OES. The extracellular medium from untreated MDA-MB-231 cells (Control medium) contained low levels of iron, whereas extracellular medium from DMSA-IONP-treated cells before centrifugation showed a high iron content (100 µg iron/mL). After centrifugation, the iron content in the extracellular medium from DMSA-IONP-treated cells was significantly reduced, reaching negligible levels (9 µg iron/mL, Figure S8a). To assess if these DMSA-IONPs might affect local cell viability, SVEC4-10 cells were first treated with DMSA-IONPs for 24 h (60 and 125 µg/ml) and their viability was assessed by Annexin-PI staining and flow cytometry (Figure S8b). DMSA-IONP internalization was studied by TEM in this endothelial cell line (Figure S8c) and their iron content was evaluated by ICP-OES (Figure S8d). DMSA-IONP internalized by SVEC4-10 cells accumulated in endosomal-like compartments, however after a 24 h exposure the DMSA-IONPs internalized by these cells did not produce ROS as evaluated by flow cytometry to assess DHR staining (Figure S8e), unlike the BC cells (Fig. 1d).

Since DMSA-IONPs do not influence SVEC4-10 cell viability or their ROS production, we evaluated if CM from DMSA-IONP treated MDA-MB-231 cells affects SVEC4-10 cell viability using the metabolic PrestoBlue assay (Figure S9a), while also performing a Crystal Violet cell confluence assay (Figure S9b) and assessing Annexin-PI staining by flow cytometry (Figure S9c). Treating SVEC4-10 cells with MDA-MB-231 cell CM-Control or CM-DMSA-IONP, reduced their metabolic rate (Figure S9a) without significantly affecting cell viability, witnessed by the lack of a significant effect on cell confluency (Figure S9b) or cell death (Figure S9c).

**Fig. 8:**
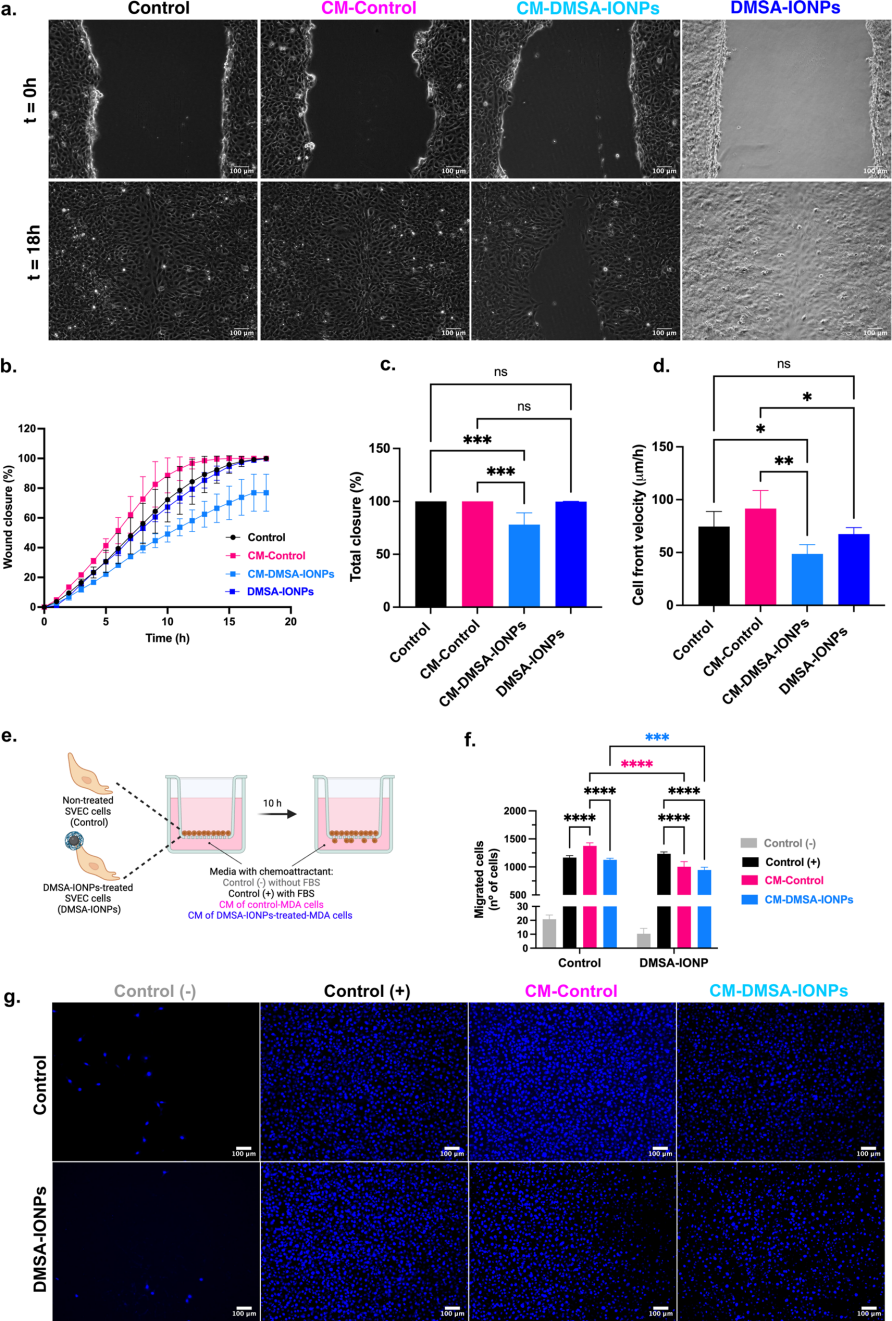
Indirect effects of CM from untreated MDA-MB-231 cells and those treated with DMSA-IONPs on SVEC4-10 cell migration.** (a)** Collective SVEC4-10 cell migration in wound-healing assays. SVEC4-10 cells were left untreated (control), treated with CM of untreated MDA-MB-231 cells (CM-Control), with CM of DMSA-IONP treated MDA-MB-231 cells (CM-DMSA-IONPs) or treated with DMSA-IONPs (60 µg/ml) for 24 h. Scale bar: 100 μm. **(b)** Relative wound closure over time. **(c)** Relative wound closure at the final time point of 18 h. **(d)** Cell front velocity over time. **(e)** Individual SVEC4-10 cell migration in transwell assays. The upper chamber cells were left untreated or treated with DMSA-IONPs (60 µg/ml), while the medium in the lower chamber was: medium with no FBS (- control), medium with 10% FBS (+ control), CM-Control, or CM-DMSA-IONPs. **(f)** The number of cells that migrated to the lower chamber. **(g)** Images of cells migrated to the lower chamber at the final time point of 10 h: the upper panel corresponds to untreated (Control) cells migrating to the lower chamber; the lower panel corresponds to DMSA-IONP treated cells migrating to the lower chamber. Scale bar: 100 μm. The data is the mean ± SD (*n* = 3 experimental replicates with 3–5 picture frames imaged for each condition). One-way ANOVA with Tukey’s multiple comparison test was used in wound-healing experiment, whereas two-way ANOVA with Sidák’s multiple comparison test was used in transwell experiments to analyze the differences between untreated SVEC4-10 cells and those in contact with CM-Control, CM-DMSA-IONP or DMSA-IONPs: *p < 0.05, **p < 0.01, ***p < 0.001, **** *p* < 0.0001

Subsequently, the effect of the MDA-MB-231 CM on SVEC4-10 cell migration and chemoattraction was evaluated, initially performing a wound healing assay to monitor cell migration over 18 h (Fig. 8a-d). Exposure to CM-Control significantly increased the migration rate of SVEC4-10 cells (Fig. 8b) with the wound completely closed after 18 h (Fig. 8c), and adopting a higher CFV than untreated cells (Fig. 8d). By contrast, treatment of SVEC4-10 cells with CM-DMSA-IONPs significantly reduced their migration rate (Fig. 8b), only achieving 75% wound closure after 18 h (Fig. 8c), and with a significantly slower CFV than untreated and CM-Control treated cells (Fig. 8d). Interestingly, treatment of SVEC4-10 cells with DMSA-IONPs did not affect their migration (Fig. 8a-d).

As the CM-Control and CM-DMSA-IONP significantly altered the capacity of SVEC4-10 cells to migrate, although in opposite ways, we evaluated the potential of these CMs to attract endothelial SVEC4-10 cells in a transwell migration assay (Fig. 8e). SVEC4-10 cells were left untreated or treated with DMSA-IONPs for 24 h and then plated on the top of a transwell insert, while different media were added to the bottom compartment (Fig. 8e) and the cells were left to migrate for 10 h: culture medium without FBS (- Control), culture medium with 10% FBS (+ Control), CM-Control and CM-DMSA-IONPs. When the cells were subsequently fixed and stained with DAPI for counting (Fig. 8f and g), untreated SVEC4-10 cells displayed greater chemotaxis towards CM-Control than towards CM-DMSA-IONP. By contrast, treating SVEC4-10 cells with DMSA-IONPs also affected their chemotaxis towards both CM-Control and CM-DMSA-IONP (Fig. 8f and g). Hence, treating MDA-MB-231 BC cells with DMSA-IONPs could interfere with the production/secretion of chemoattractants (Fig. 8f and g). To assess this, the extracellular content of VEGF and MMP2 were determined by ELISA (Figure S10). No change on VEGF extracellular content was observed, whereas MMP2 content was reduced by 2-fold in extracellular medium of DMSA-IONP-treated cells compared to untreated cells (Figure S10b). Therefore, treating of BC cells with DMSA-IONPs not only affected BC cell migration but also, it indirectly affects endothelial cell proliferation, migration and chemotaxis towards BC cells.

## Discussion

Tumor cell spreading and invasion is a hallmark of cancer, and it involves a complex interplay between cancer cells and their microenvironment [84]. Migrating cells undergo metabolic and oxidative rearrangements that render them more sensitive to changes in their redox homeostasis as they metastasize. Pro-oxidant therapies are potentially interesting strategies as they may compromise the viability and migratory capacity of cancer cells during metastasis, pushing oxidative stress beyond viable thresholds. This study set out to investigate how oxidative stress induced by DMSA-IONPs could hamper BC cell migration and its value as a strategy to combat metastasis. In addition, we also assessed how the passage of DMSA-IONPs through endo-lysosomal system affects paracrine communication between cancer cells and the tumor microenvironment, specifically their interaction with endothelial cells.

DMSA-IONPs are candidates for pro-oxidant therapy since they induce ROS production by MDA-MB-231 BC cells. The chemical nature of the DMSA coating, establishing carboxyl groups (-COOH) at the IONP surface, influences their redox potential [43]. Relative to hydroxyl (-OH) or amine (-NH_2_) groups, the importance of these carboxyl group at the surface for the redox potential of different organic molecules has been highlighted previously [85]. Indeed, the pro-oxidant capacity of DMSA-IONPs inside cells is likely to derive from their intracellular localization in autolysosomes with an acidic pH, which facilitates IONP degradation and the liberation of iron ions that can generate ROS inside cells through the Fenton Reaction [86]. The presence of NPs inside lysosomes concomitant with a higher cytotoxic effect related to ROS production has also been described elsewhere. The presence of cerium oxide NPs inside lysosomes induced cytotoxicity in lung carcinoma cell lines, whereas no cytotoxicity of cardiac myocytes or kidney cells were evident when these NPs accumulated in the cytoplasm [87]. In a study of commercial Resovist^®^ IONPs, their accumulation inside lysosomes of macrophages [88] and microglia cells [89] affected lysosomal function and ROS production. Therefore, inside autolysosomes DMSA-IONPs are directly responsible for ROS production.

DMSA-IONPs affect cell migration and invasion by inducing actin cytoskeleton rearrangements. DMSA-IONPs affect the cell size and actin distribution, with treated cells producing fewer invadosomes and with lamellipodia that occupy less of the membrane, altering the intensity of actin aggregation in distinct cell structures and affecting cortical F-actin thickness. By contrast, DMSA-IONPs had no effect at all on MDA-MB-231 spheroid cell spreading, either when the spheroids were exposed to the IONPs or if they were present in the medium. Polymeric NPs limited the collective migration of CT26 tumor spheroids by enhancing cell-cell interactions due to their presence in the membrane [90]. Previous studies determined the beneficial effect of PEI-coated IONPs (PEI-IONPs) on the migration and invasion of pancreatic tumor cells, dampening their migration in wound closure assays and decreasing the number of invadopodia in these cells [91]. These PEI-IONPs also had beneficial effects on macrophages, as they activated a more M1 profile, in addition to positively modulating podosome formation and affecting their migration [92]. PEI-IONPs also affect endothelial cell migration and their ability to form endothelial tubes, demonstrating anti-metastatic activity [93]. Finally, parallel studies determined the role of aminopropyl-silane-coated IONPs (APS-IONPs), DMSA-IONPs and aminodextran-coated IONPs (AD-NPs) in the migration and invasion capacity of macrophages, indicating that although they do not have a significant effect on migration, they can enhance the invasion capacity of macrophage cell lines [94]. Other studies demonstrated the involvement of various types of NPs in cell migration. For example, studies with polydopamine-coated IONPs showed increased proliferation, migration and secretion of the vascular endothelial growth factor (VEGF) by mesenchymal stem cells, producing a beneficial effect in repairing lesions [95]. A study with polymeric NPs demonstrated beneficial effects in reducing the migration and collective diffusion of CT26 carcinoma cell tumor aggregates [90]. Similarly, gold NPs were shown to reduce the migration and invasion capacity of ovarian cancer cells [96] and silica NPs were seen to inhibit the migration of glioblastoma tumor cells [97]. Thus, there is considerable evidence that NPs affect cell migration in different ways and that they can produce anti-metastatic effects by inhibiting tumor cell migration.

The potential of DMSA-IONPs to alter cell migration and the actin cytoskeleton might be due to their pro-oxidant effects, particularly as DMSA-IONPs increase oxidation as assessed by protein carbonylation. Oxidative stress can induce the oxidation of cysteine or methionine residues in actin, affecting its polymerization. Oxidation of G-actin by H_2_O_2_ decreases actin polymerization [98], while polymerization of oxidized G-actin creates more fragmented and fragile actin filaments [99]. Moreover, the oxidation of actin through the action of the redox enzyme MICAL1 is responsible for oxidation-mediated depolymerization of actin during cell division [100]. Oxidative stress can induce cytoskeleton remodeling without necessary triggering actin degradation, leading to cellular compaction and thus reduction of cell size [58, 99]. Consequently, G-actin/F-actin ratios might change while total actin levels remain constant. Besides, the oxidation of actin-binding proteins (ABPs) can impair actin treadmilling, reducing lamellipodia and stress fiber maintenance, leading to decreased apparent cell size [17, 18]. Furthermore, ROS can enhance myosin II activity increasing cortical tension and thereby reorganizing the spatial organization of actin cytoskeleton while actin content is preserved leading to retracted cell edges and increased cell rounding [101, 102]. These observations, together with our results suggest that oxidation of actin or ABPs by DMSA-IONPs might trigger actin depolymerization or cytoskeletal reorganization rather than a reduction in actin abundance, which would be responsible for the smaller cell size and their lower number of invadosomas affecting the cell’s capacity to migrate. Besides, thicker extension of cortical F-actin was observed upon DMSA-IONP treatment which may also influence cell shape and migration capacity. Cortical tension can be modulated by F-actin reorganization and is affected by cortex thickness [103]. Several studies have highlighted the importance of cortex thickness in regulating cell migration. For example, increased membrane-cortex adhesion and higher F-actin density have been associated with a more differentiated, non-migratory phenotype compared with stem cells [104]. Other studies have shown that cortex thickness differs markedly between elongated and compacted cells, with compacted cells almost doubling their cortex thickness [105]. Therefore, cortical F-actin organization can influence on cell shape, affecting both cell size and migratory behavior. Although N-acetylcysteine (NAC) significantly attenuates oxidative stress, its antioxidant effect is not complete (Figure S1b), which may explain why cell migration is not fully restored (Figure S1 c-f). NAC primarily acts as a precursor of glutathione and a general ROS scavenger, but it does not fully re-establish the localized redox signaling required for proper migratory responses. In addition, oxidative modifications of proteins, particularly irreversible cysteine oxidations or carbonylation events, may partially persist despite NAC treatment [106], continuing to impair cytoskeletal dynamics and adhesion turnover. The partially recover of cell migration therefore supports the notion that DMSA-IONPs impair cell migration through ROS-dependent mechanisms. Moreover, DMSA-IONPs may also disrupt cell migration in a ROS-independent way by physically interfering with actin or microtubule filaments, altering membrane integrity, cytoskeletal organization, or cell stiffness [107]. Thus, the incomplete restoration of migration in the presence of NAC suggests that both ROS-dependent and ROS-independent mechanisms contribute to the impaired migratory phenotype.

DMSA-IONPs are known to affect the disposition of mitochondria in cells [43] and although they did not affect the localization of mitochondria in migrating structures here, DMSA-IONPs were previously seen to affect OXPHOS functionality in MDA-MB-231 cells, thereby hampering ATP production by mitochondria [43]. Blocking OXPHOS has been proposed to impair mitochondrial trafficking and suppress tumor cell invasion by affecting interactions with focal adhesions [64]. Thus, the mitochondria near to invadosomes of DMSA-IONP treated cells may provide less ATP than those of untreated cells. This reduced energy supply, together with fewer invadosomes and the largely oxidized actin in DMSA-IONP treated cells may impede the formation of migratory structures and affect the capacity of cells to migrate.

We also gleaned more information here on the mechanisms of DMSA-IONP internalization and their intracellular distribution, indicating how this might affect UCPS. The type of endocytosis determines the rate of IONP internalization, with macropinocytosis characterized by internalizing large quantities of material as the elongations of the membrane that form encapsulate liquid material together with IONPs. By contrast, clathrin- and caveolin-dependent endocytosis are characterized by more slowly endocytosing less material [30, 65]. Therefore, the low concentration of intracellular iron (4 pgFe/cell) after DMSA-IONP treatment is more consistent with clathrin- and caveolin-dependent internalization. Previous studies indicated that DMSA-IONPs were internalized by Raw264.7 macrophages and the Pan02 pancreatic cancer cell line through macropinocytosis, clathrin- and caveolin-dependent endocytosis [45]. This reflected their cell-dependent intracellular fate and trafficking, as autolysosomes in macrophages have relatively more lytic enzymes and an acidic nature relative to the pancreatic cell line [45].

The presence of DMSA-IONPs inside autolysosomes could be related to the UCPS as it might interfere with the exocytosis of lysosomes, autophagosomes or MVBs. The presence of Resovist^®^ inside lysosomes of microglial cells was associated with dampened interleukin 1β (IL-1β) production via the secretory lysosomal pathway [89]. EV-like structures can be released spontaneously in response to biological or chemical triggers and as IONPs interfere with the endo-lysosomal system, they can influence the generation and release of EV-like structures from cells [108].

Oxidative stress has been shown to modulate endosomal trafficking and maturation, thereby affecting unconventional protein secretion pathways (UCPS) and influencing EV secretion. ROS generation can modify proteins involved in signal pathways that regulate both the amount and the molecular cargo of secreted EVs [109]. Redox signaling can also regulate intracellular trafficking through oxidative modifications of vesicle-transport machinery. For example, ROS can alter the activity or localization of Rab GTPases and SNARE proteins via thiol oxidation, thereby modifying endosomal trafficking and vesicle fusion [110]. Unconventional secretory pathways (UCPS) rely on vesicle trafficking machinery, including Rab GTPases and SNARE proteins, which mediate SNARE-dependent intracellular membrane fusion [111]. SNAREs are key fusion components, with v-SNARE located on vesicles and t-SNAREs on target membranes [112], whereas Rab GTPases mediate the formation, transport and fusion of intercellular vesicles controlling the recognition and tethering to the target membrane [113]. UCPS has been shown to depend on multiple v- and t-SNAREs such as members from the vesicle-associated membrane protein (VAMP) family VAMP3, VAMP7, VAMP8 and SEC22B, among other SNARE proteins and Rab GTPases [111]. VAMP7 and VAMP8 are enriched in recycling and late endosomes/MVBs; VAMP7 is also present in lysosomes and other compartments [111]. These VAMPs interact with plasma-membrane t-SNAREs to mediate secretion. Notably, VAMP7/Syntaxin 1/SNAP25 and VAMP7/Syntaxin 3/SNAP-23 mediate the fusion of secretory late endosomes with the plasma membrane [114], whereas VAMP7 along with t-SNARES Syntaxin4 and SNAP23 mediates lysosomal secretion in fibroblasts [115] and lysosomal secretion of ATP in astrocytes [116]. Moreover, Rab 11, Rab 3a and Sect. 15 are required for lysosome exocytosis [117]. In addition, SEC22B interacts with tripartite motif-containing 16 (TRIM16) to facilitate cargo release via secretory autophagy [118]. In our previous work [45], proteomic analysis of endosomes from DMSA-IONP-treated cells revealed cell-type-specific in Rab and SNARE composition, consistent with active fusion and cargo-sorting processes. Endosomes containing DMSA-IONPs were enriched in syntaxin 4, syntaxin 5, syntaxin 7 and syntaxin 12, in VAMP7 and VAMP8. Because oxidative stress can modify the trafficking proteins present in different vesicles, DMSA-IONP-induced ROS could contribute to UCPS alterations by affecting Rab/SNARE-dependent trafficking.

The link between ROS and EV release is further supported by data showing that the co-incubation with antioxidants which reduced the exocytosis of EVs mediated by polycyclic aromatic hydrocarbons (PAHs) [119]. ROS can also enhance the number of MVBs by limiting their degradation in lysosomes and promoting exocytosis [120]. Moreover, oxidative stress impairs autophagosome-lysosome fusion by limiting lysosomal localization of VAMP8, a SNARE protein [121], and the inhibition the endolysosome fusion increases exosome secretion [122]. Together these findings support that oxidative stress induced by DMSA-IONPs could impair late endosome-lysosome fusion, thereby stimulating MVB exocytosis. Some studies have linked treating cells with IONPs with enhanced EV biogenesis, such as by human induced pluripotent iPSK3 stem cells [123], whereas no such affects were seen in astrocytes [124]. Here, DMSA-IONPs increase the accumulation of Rab7 at the PM, which could reflect altered exocytosis of EV-like structures by MDA-MB-231 cells. Lysosomes play a role in regulating EV biogenesis and release [74], as altering lysosomal function through a shift in their pH [125, 126] or overloading them with damaged molecules [127, 128] can increase EV release. Thus, overloading lysosomes with DMSA-IONPs could drive MVB or late endosome release into the extracellular space to maintain cell homeostasis.

Moreover, the cortical actin network regulates vesicle transport toward the plasma membrane, vesicle docking, and membrane fusion. At resting conditions, cortical actin can act as a physical barrier that entraps and limits the access of secretory vesicles to the plasma membrane. Upon cellular stimulation, actin undergoes dynamic remodeling that facilitates vesicle trafficking and fusion. In addition, several studies have shown that following exocytosis, actin can assemble around fused vesicles forming a coat which contributes to the efficient vesicle content release via actomyosin contractibility [129]. Myosin V motor activity further regulates vesicle transport [130]. In this context, changes in the subcellular localization of actin or in its polymerization state could alter the organization of the cortical actin network and thereby affect different steps of the secretory process. Therefore, the oxidation of actin cytoskeleton triggered by DMSA-IONP treatment alters actin cytoskeleton reorganization, affecting lamellipodia, invadosomes and F-actin cortical thickness, which could contribute to explaining potential changes in vesicular secretion efficiency.

It was also evident here how the presence of DMSA-IONPs inside lysosomes limits lysosome exocytosis, while stimulating the exocytosis of late endosomes or MVBs. Peripheral lysosomes that are more likely to undergo exocytosis are characterized by having a less acidic pH [76], which could explain why changes in lysosomal exocytosis does not affect extracellular pH as the lysosome released by untreated cells might not be acidic. It is known that oxidative stress mediated by IONPs is related to acidification in lysosomes, as catalytic activity of IONPs strongly depends on pH. IONPs exhibit a peroxidase catalytic activity in acidic environments mediating Fenton reaction and the generation of ·OH from H_2_O_2_ [131], whereas in a less acidic or neutral pH IONPs shift their peroxidase activity towards a catalase activity where H_2_O_2_ is neutralized and no ROS is formed [132]. Therefore, acidic pH correlates with a degradative activity of lysosomes together with the generation of ROS. Acidic lysosomes are typically perinuclear, whereas secretory lysosomes are less acidic and more peripheral [76]. As shown in Fig. 5, lysosomes in DMSA-IONP-treated cells remain perinuclear and acidic (Figure S5), supporting the idea that they are degradative rather than secretory, which is consistent with altered exocytosis. Lysosomal exocytosis represents a source of local membrane that could serve to build the large protrusions that promote tissue invasion [42] and the release of hydrolytic enzymes that promotes ECM degradation [82]. Thus lysosome exocytosis might facilitate cancer cell migration and invasion during metastasis. Here, DMSA-IONPs reduced the cell structures associated with migration and lysosomal exocytosis, affecting the migratory and invasive capacity of MDA-MB-231 BC cells. Therefore, DMSA-IONPs appear to produce anti-metastatic effects and could represent therapeutic agents to combat cancer cell invasiveness.

Lysosome release has also been linked to chemoresistance, as the accumulation of anticancer drugs in lysosomes triggers their exocytosis [133]. As such, DMSA-IONPs could impede the acquisition of chemoresistance. Moreover, DMSA-IONPs trigger autophagy and alter secretory autophagy, dampening the accumulation of LC3B at the PM. Treating cells with IONPs was previously seen to modulate the induction of autophagy [134–137] and in some cases, the induction of autophagy was associated with enhanced cancer cell death [136, 137]. One of the earliest examples of extracellular protein released by secretory autophagy has been the proinflammatory IL1β cytokine [138], although recently other cargoes secreted by secretory autophagy have been defined like MMP2, MMP9, IL-6, IL-8, type I collagen and fibronectin [37]. Some recent therapeutic approaches focus on secretory autophagy [83], targeting classic autophagy signaling, including PI3KC3/VPS34 [139] or ATG4, 5 and 7 [140–142], which can also block the fusion of autophagosomes with lysosomes. In addition, the classic autophagy inhibitor chloroquine (CQ) is being studied as a potential anti-cancer therapy [143]. Therefore, although DMSA-IONPs increase the intracellular autophagy in general, they also decrease the LC3B present at the PM, which indicates a blockage of secretory autophagy. This is also concomitant with the accumulation of less Lamp-2 at the PM. When the extracellular content of VEGF and MMP2 was studied, no change on VEGF extracellular content was observe, whereas MMP2 extracellular content was reduced by 2-fold. Although the extracellular content of VEGF was not significantly altered, our data suggest that unconventional secretory pathways (UCPS) may nonetheless be affected, as Lamp-2 and LC3B markers on the plasma membrane were reduced whereas Rab7 increased. This apparent discrepancy could be explained by alternative trafficking routes for VEGF, as it has been reported to localize within EVs from breast cancer tumor cells [144]. In this context, it is possible that VEGF secretion is maintained through EV production via MVB exocytosis. Supporting this hypothesis, as DMSA-IONP treatment was shown to increase EV secretion, this enhanced vesicle release may preserve extracellular VEGF levels despite the observed change in membrane-bound UCPS markers. On the other hand, MMP2 extracellular content was shown to be reduced upon DMSA-IONP-treatment, reinforcing the hypothesis of affected UCPS trafficking, as MMP2 has been shown to be secreted via secretory lysosomal and autophagy pathways [145].

Finally, we studied how affecting the UCPS might indirectly affect endothelial cell migration and chemotaxis. Although no direct effects on cell migration and proliferation were found when endothelial cells were treated with DMSA-IONPs, treating BC cells with DMSA-IONPs indirectly affected endothelial cell proliferation and migration. IONPs may directly affect the angiogenic capacity of endothelial cells and PEI-IONPs generate a pro-inflammatory profile in endothelial cells, inhibiting migration and vessel formation [93]. Polyglucose sorbitol carboxymethylether (PSC)-coated IONPs also provoke morphological changes in endothelial cells that are dependent on ROS production [146]. Here, DMSA-IONPs do not directly affect the migration capacity of SVEC4-10 cells evident in wound healing assays but they do interfere with their chemotaxis. Moreover, CM-DMSA-IONP directly interferes with SVEC4-10 migration and chemotaxis. Clearly, treatment of MDA-MB-231 cells with DMSA-IONPs affects their secretion of pro-inflammatory or pro-angiogenic cytokines, probably through the effects on the UCPS that alter lysosome and autophagosome secretion. The CM of tumor cell lines like MDA-MB-231 and MCF-7 cells induces the secretion of pro-angiogenic cytokines and enhances HUVEC endothelial cell angiogenesis, although treatment of these cells with nanorods affects endothelial tube formation [147]. Elsewhere, gold-NPs disrupt the communication between tumor and endothelial cells, as the CM of tumor cells induced migration and endothelial tube formation, whereas prior treatment of the endothelial cells with gold-NPs dampened these effects on migration and tube formation [148]. Moreover, enhanced ROS production was proposed as a primary chemoattractant [149] and thus, reducing extracellular ROS through DMSA-IONP treatment could also contribute to the weakened SVEC4-10 cell chemoattraction. In addition to affecting endothelial cell migration and chemotaxis, treating BC cells with DMSA-IONPs also reduced the metabolic activity of endothelial cells (Figure S9a). Increasing evidence indicates that EVs play a significant role in modulating stromal cell metabolism. Through their cargo, tumor-derived EVs promote glycolytic reprogramming [150], mediated the transfer of miRNAs and lncRNAs [151], as well as glycolytic enzymes such as pyruvate kinase M2 (PKM2) [152]. DMSA-IONPs treatment of MDA-MB-231 was previously shown to modulate cancer cell metabolism by reducing mitochondrial OXPHOS without affecting glycolysis [43]. This alteration in OXPHOS may influence the metabolic composition of BC-derived EVs, thereby contributing to the reduction in endothelial cell metabolic activity observed in endothelial cells (Figure S9a). Thus, DMSA-IONP-treatment of BC cells appears to modulate both the number and size of EV-like structure (Figure S6b), as well as their cargo, ultimately impacting neighboring microenvironment cells. Additionally, extracellular ROS levels were altered following DMSA-IONP treatment, as CM-Control medium exhibited higher ROS levels than CM-DMSA-IONP medium (Figure S7a). This decrease in extracellular ROS levels is consistent with reduced lysosomal exocytosis (Fig. 6) and may also contribute to the lower metabolic rate observed in endothelial cells. Endothelial cell proliferation is highly sensitive to redox balance, with reduced ROS favoring a quiescent, non-proliferative phenotype, while elevated ROS levels promoted inflammatory and proliferative responses [153]. Therefore, DMSA-IONP treatment modifies endolysosomal trafficking, reducing lysosomal exocytosis while enhancing exocytosis of MVB-like or EV-like vesicles. These alterations in UCPS activity modify the extracellular medium, increasing EV-like or MVB-like vesicles and reducing ROS, thereby affecting endothelial cell migration and metabolic activity. These studies confirm that DMSA-IONPs can potentially affect endothelial cell migration, albeit indirectly by modulating the production and secretion of the pro-angiogenic or pro-inflammatory cytokines that directly affect the UCPS. Therefore, DMSA-IONPs could represent inhibitors of metastasis in a BC model, as they may directly affect BC cell migration and indirectly affect the migration of endothelial cells.

## Conclusions

NP-based pro-oxidant anticancer therapies have been established as promising alternatives to conventional cancer treatments. During cancer cell migration, cells undergo oxidative rearrangements that render migrating cells more sensitive to external changes in their redox homeostasis. Here, we demonstrated that DMSA-IONPs induce ROS production in the metastatic MDA-MB-231 BC cell line, producing the reorganization of actin, and affecting cell size, the actin distribution in the cell, the number of invadosomes per cell, as well as the extension of lamellipodia. Thereafter, cell migration and invasion were hampered. Moreover, the presence of DMSA-IONPs in the endo-lysosomal compartments affected the UCPS, inducing stronger exocytosis of late endosomes or MVBs while blocking the exocytosis of lysosomes or autophagosomes. Together these changes affect endothelial cell migration and chemotaxis, and hence paracrine communication. Novel cancer therapies are exploring secretory autophagy and lysosome secretory pathways as new targets to not only affect cancer cells directly but also, their microenvironment. Overall, as DMSA-IONPs have been considered promising tools in vivo due to their limited toxicity and long-term bioavailability, we propose that DMSA-IONPs might also be used to alter the redox balance of highly metastatic cells, not only affecting their migration but also, somehow limiting their paracrine communication with the cells in the tumor microenvironment.

## Supplementary Information

Supplementary Material 1

## Data Availability

All data generated or analyzed during this study are available upon reasonable request.

## References

[1] Bray F, Laversanne M, Sung H, Ferlay J, Siegel RL, Soerjomataram I, Jemal A. Global cancer statistics 2022: GLOBOCAN estimates of incidence and mortality worldwide for 36 cancers in 185 countries. CA Cancer J Clin. 2024;74(3):229–63. 38572751 10.3322/caac.21834

[2] Park M, Kim D, Ko S, Kim A, Mo K, Yoon H. Breast cancer metastasis: mechanisms and therapeutic implications. Int J Mol Sci. 2022;23(12):6806. 35743249 10.3390/ijms23126806 PMC9224686

[3] Tang DD, Gerlach BD. The roles and regulation of the actin cytoskeleton, intermediate filaments and microtubules in smooth muscle cell migration. Respir Res. 2017;18(1):54. 28390425 10.1186/s12931-017-0544-7 PMC5385055

[4] Yamaguchi H, Condeelis J. Regulation of the actin cytoskeleton in cancer cell migration and invasion. Biochim Biophys Acta. 2007;1773(5):642–52. 16926057 10.1016/j.bbamcr.2006.07.001 PMC4266238

[5] Pollard TD, Cooper J. Actin, a central player in cell shape and movement. Science. 2009;326(5957):1208–12. 19965462 10.1126/science.1175862 PMC3677050

[6] Madan S, Uttekar B, Chowdhary S, Rikhy R. Mitochondria lead the way: mitochondrial dynamics and function in cellular movements in development and disease. Front Cell Dev Biol. 2022;9:781933. 35186947 10.3389/fcell.2021.781933 PMC8848284

[7] Chen W, Zhao H, Li Y. Mitochondrial dynamics in health and disease: mechanisms and potential targets. Signal Transduct Target Ther. 2023;8(1):333. 37669960 10.1038/s41392-023-01547-9 PMC10480456

[8] Zhao J, Zhang J, Yu M, Xie Y, Huang Y, Wolff DW, Abel PW, Tu Y. Mitochondrial dynamics regulates migration and invasion of breast cancer cells. Oncogene. 2013;32(40):4814–24. 23128392 10.1038/onc.2012.494 PMC3911914

[9] Desai SP, Bhatia S, Toner M, Irimia D. Mitochondrial localization and the persistent migration of epithelial cancer cells. Biophys J. 2013;104(9):2077–88. 23663851 10.1016/j.bpj.2013.03.025 PMC3647149

[10] Humphries BA, Zhang A, Buschhaus JM, Bevoor A, Farfel A, Rajendran S, et al. Enhanced mitochondrial fission inhibits triple-negative breast cancer cell migration through an ROS-dependent mechanism. iScience. 2023;26(6):106788. 37235049 10.1016/j.isci.2023.106788 PMC10206500

[11] Go YM, Jones DP. The redox proteome. J Biol Chem. 2013;288(37):26512–20. 23861437 10.1074/jbc.R113.464131 PMC3772199

[12] Jones DP. Redox sensing: orthogonal control in cell cycle and apoptosis signalling. J Intern Med. 2010;268:432–48. 20964735 10.1111/j.1365-2796.2010.02268.x PMC2963474

[13] Trachootham D, Lu W, Ogasawara MA, Nilsa RD, Huang P. Redox regulation of cell survival. Antioxid Redox Signal. 2008;10(8):1343–74. 18522489 10.1089/ars.2007.1957 PMC2932530

[14] Ježek JCK, Strich R. Reactive Oxygen Species and Mitochondrial Dynamics: The Yin and Yang of Mitochondrial Dysfunction and Cancer Progression. Antioxid (Basel). 2018;7(1):13. 10.3390/antiox7010013 PMC5789323 29337889

[15] Balta E, Kramer J, Samstag Y. Redox regulation of the actin cytoskeleton in cell migration and adhesion: on the way to a spatiotemporal view. Front Cell Dev Biol. 2021;8:618261. 33585453 10.3389/fcell.2020.618261 PMC7875868

[16] Dalle-Donne I, Milzani A, Colombo R. The tert-butyl hydroperoxide-induced oxidation of actin Cys-374 is coupled with structural changes in distant regions of the protein. Biochemistry. 1999;38(38):12471–80. 10493817 10.1021/bi990367k

[17] Balta E, Hardt R, Liang J, Kirchgessner H, Orlik C, Jahraus B, et al. Spatial oxidation of L-plastin downmodulates actin-based functions of tumor cells. Nat Commun. 2019;10(1):4073. 31501427 10.1038/s41467-019-11909-z PMC6733871

[18] Samstag Y, John I, Wabnitz GH. Cofilin: a redox sensitive mediator of actin dynamics during T-cell activation and migration. Immunol Rev. 2013;256(1):30–47. 24117811 10.1111/imr.12115 PMC3884758

[19] Bergerhausen L, Grosche J, Meißner J, Hecker C, Caliandro MF, Westerhausen C, Kamenac A, Rezaei M, Mörgelin M, Poschmann G, Vestweber D, Hanschmann EM, Eble JA. Extracellular Redox Regulation of α7β Integrin-Mediated Cell Migration Is Signaled via a Dominant Thiol-Switch. Antioxid (Basel). 2020;9(3):227. 10.3390/antiox9030227 PMC7139957 32164274

[20] Möller MN, Orrico F, Villar SF, López AC, Silva N, Donzé M, Thomson L, Denicola A. Oxidants and Antioxidants in the Redox Biochemistry of Human Red Blood Cells. ACS Omega. 2022;8(1):147–68. 36643550 10.1021/acsomega.2c06768 PMC9835686

[21] Tasdogan A, Ubellacker J, Morrison SJ. Redox Regulation in Cancer Cells during Metastasis. Cancer Discov. 2021;11(11):2682–92. 34649956 10.1158/2159-8290.CD-21-0558 PMC8563381

[22] Wiel C, Le Gal K, Ibrahim MX, Jahangir CA, Kashif M, Yao H, et al. BACH1 Stabilization by Antioxidants Stimulates Lung Cancer Metastasis. Cell. 2019;178(2):330–45. 31257027 10.1016/j.cell.2019.06.005

[23] Sayin VI, Ibrahim MX, Larsson E, Nilsson JA, Lindahl P, Bergo MO. Antioxidants accelerate lung cancer progression in mice. Sci Transl Med. 2014;6(221):221ra15. 24477002 10.1126/scitranslmed.3007653

[24] Wang H, Liu X, Long M, Huang Y, Zhang L, Zhang R, et al. NRF2 activation by antioxidant antidiabetic agents accelerates tumor metastasis. Sci Transl Med. 2016;8(334):334ra5. 10.1126/scitranslmed.aad6095 27075625

[25] Piskounova E, Agathocleous M, Murphy MM, Hu Z, Huddlestun SE, Zhao Z, Leitch AM, Johnson TM, DeBerardinis RJ, Morrison SJ. Oxidative stress inhibits distant metastasis by human melanoma cells. Nature. 2015;527(7577):186–91. 26466563 10.1038/nature15726 PMC4644103

[26] Meyerstein D. Re-examining fenton and fenton-like reactions. Nat Rev Chem. 2021;5(9):595–7. 37118415 10.1038/s41570-021-00310-4

[27] Ranji-Burachaloo H, Gurr PA, Dunstan DE, Qiao GG. Cancer treatment through nanoparticle-facilitated fenton reaction. ACS Nano. 2018;12(12):11819–37. 30457834 10.1021/acsnano.8b07635

[28] Malhotra N, Lee JS, Liman RAD, Ruallo JMS, Villaflores OB, Ger TR, et al. Potential toxicity of iron oxide magnetic nanoparticles: a review. Molecules. 2020;25(14):3159. 32664325 10.3390/molecules25143159 PMC7397295

[29] Attia NF, Eman M, Abd E-M, El-Aqapa HG, Elashery SEA, Eltaweil AS, El Kady M, Khalifa SAM, Hawash HB, El-Seedi HR. Iron oxide nanoparticles and their pharmaceutical applications. Appl Surf Sci Adv. 2022;11:100284.

[30] Sousa de Almeida M, Susnik E, Drasler B, Taladriz-Blanco P, Petri-Fink A, Rothen-Rutishauser B. Understanding nanoparticle endocytosis to improve targeting strategies in nanomedicine. Chem Soc Rev. 2021;50(9):5397–434. 33666625 10.1039/d0cs01127d PMC8111542

[31] Wu H, Yin JJ, Wamer WG, Zeng M, Lo YM. Reactive oxygen species-related activities of nano-iron metal and nano-iron oxides. J Food Drug Anal. 2014;22:1. 24673906 10.1016/j.jfda.2014.01.007 PMC9359154

[32] Petters C, Thiel K, Dringen R. Lysosomal iron liberation is responsible for the vulnerability of brain microglial cells to iron oxide nanoparticles: comparison with neurons and astrocytes. Nanotoxicology. 2016;10(3):332–42. 26287375 10.3109/17435390.2015.1071445

[33] Liu ZL, Chen HH, Zheng LL, Sun LP, Shi L. Angiogenic signaling pathways and anti-angiogenic therapy for cancer. Signal Transduct Target Ther. 2023;8(1):198. 37169756 10.1038/s41392-023-01460-1 PMC10175505

[34] Mao X, Xu J, Wang W, Liang C, Hua J, Liu J, et al. Crosstalk between cancer-associated fibroblasts and immune cells in the tumor microenvironment: new findings and future perspectives. Mol Cancer. 2021;20(1):131. 34635121 10.1186/s12943-021-01428-1 PMC8504100

[35] Zhang J, Zhang X, Liu G, Chang D, Liang X, Zhu X, et al. Intracellular trafficking network of protein nanocapsules: endocytosis, exocytosis and autophagy. Theranostics. 2016;6(12):2099–113. 27698943 10.7150/thno.16587 PMC5039683

[36] Padmanabhan S, Manjithaya R. Facets of autophagy based unconventional protein secretion-the road less traveled. Front Mol Biosci. 2020;7:586483. 33363205 10.3389/fmolb.2020.586483 PMC7755989

[37] Kuo IY, Hsieh C, Kuo WT, Chang CP, Wang YC. Recent advances in conventional and unconventional vesicular secretion pathways in the tumor microenvironment. J Biomed Sci. 2022;29(1):56. 35927755 10.1186/s12929-022-00837-8 PMC9354273

[38] van Niel G, D’Angelo G, Raposo G. Shedding light on the cell biology of extracellular vesicles. Nat Rev Mol Cell Biol. 2018;19(4):213–28. 29339798 10.1038/nrm.2017.125

[39] Reddy A, Caler EV, Andrews NW. Plasma membrane repair is mediated by Ca(2+)-regulated exocytosis of lysosomes. Cell. 2001;106(2):157–69. 11511344 10.1016/s0092-8674(01)00421-4

[40] Czibener C, Sherer N, Becker SM, Pypaert M, Hui E, Chapman ER, Mothes W, Andrews NW. Ca2 + and synaptotagmin VII-dependent delivery of lysosomal membrane to nascent phagosomes. J Cell Biol. 2006;174(7):997–1007. 16982801 10.1083/jcb.200605004 PMC2064391

[41] Arantes RM, Andrews NW. A role for synaptotagmin VII-regulated exocytosis of lysosomes in neurite outgrowth from primary sympathetic neurons. J Neurosci. 2006;26(17):4630–7. 16641243 10.1523/JNEUROSCI.0009-06.2006 PMC6674075

[42] Naegeli KM, Hastie E, Garde A, Wang Z, Keeley DP, Gordon KL, et al. Cell invasion in vivo via rapid exocytosis of a transient lysosome-derived membrane domain. Dev Cell. 2017;43(4):403-17.e10. 29161591 10.1016/j.devcel.2017.10.024 PMC5726793

[43] Daviu N, Portilla Y, Gómez de Cedrón M, Ramírez de Molina A, Barber DF. DMSA-coated IONPs trigger oxidative stress, mitochondrial metabolic reprograming and changes in mitochondrial disposition, hindering cell cycle progression of cancer cells. Biomaterials. 2024;304:122409. 38052135 10.1016/j.biomaterials.2023.122409

[44] Portilla Y, Fernández-Afonso Y, Pérez-Yagüe S, Mulens-Arias V, Morales MP, Gutiérrez L, et al. Different coatings on magnetic nanoparticles dictate their degradation kinetics in vivo for 15 months after intravenous administration in mice. J Nanobiotechnology. 2022;20(1):54. 36578018 10.1186/s12951-022-01747-5 PMC9795732

[45] Portilla Y, Mulens-Arias V, Paradela A, Ramos-Fernández A, Pérez-Yagüe S, Morales MP, et al. The surface coating of iron oxide nanoparticles drives their intracellular trafficking and degradation in endolysosomes differently depending on the cell type. Biomaterials. 2022;281:121365. 35038611 10.1016/j.biomaterials.2022.121365

[46] Massart R. Preparation of aqueous magnetic liquids in alkaline and acidic media. IEEE Trans Magn. 1981;17(2):1247–8.

[47] de la Presa P, Luengo Y, Multigner M, Costo R, Morales MP, Rivero G, et al. Study of heating efficiency as a function of concentration, size, and applied field in γ-Fe2O3 nanoparticles. J Phys Chem C. 2012;116(48):25602–10.

[48] Foty R. A simple hanging drop cell culture protocol for generation of 3d spheroids. J Vis Exp. 2011;51:e2720. 10.3791/2720 PMC3197119 21587162

[49] Stauffer W, Sheng H, Lim HN. Ezcolocalization: an ImageJ plugin for visualizing and measuring colocalization in cells and organisms. Sci Rep. 2018;8(1):15764. 30361629 10.1038/s41598-018-33592-8 PMC6202351

[50] Xie Y, Wolff DW, Wei T, Wang B, Deng C, Kirui JK, Jiang H, Qin J, Abel PW, Tu Y. Breast cancer migration and invasion depend on proteasome degradation of regulator of G-protein signaling 4. Cancer Res. 2009;69(14):5743–51. 19549919 10.1158/0008-5472.CAN-08-3564 PMC2741027

[51] Cervero P, Panzer L, Linder S. Podosome reformation in macrophages: assays and analysis. Methods Mol Biol. 2013;1046:97–121. 23868584 10.1007/978-1-62703-538-5_6

[52] Portilla Y, Mellid S, Paradela A, Ramos-Fernández A, Daviu N, Sanz-Ortega L, et al. Iron oxide nanoparticle coatings dictate cell outcomes despite the influence of protein coronas. ACS Appl Mater Interfaces. 2021;13(7):7924–44. 33587585 10.1021/acsami.0c20066

[53] Hurd TR, DeGennaro M, Lehmann R. Redox regulation of cell migration and adhesion. Trends Cell Biol. 2012;22(2):107–15. 22209517 10.1016/j.tcb.2011.11.002 PMC4515034

[54] Rouyère C, Serrano T, Frémont S, Echard A. Oxidation and reduction of actin: origin, impact in vitro and functional consequences in vivo. Eur J Cell Biol. 2022;101(3):151249. 35716426 10.1016/j.ejcb.2022.151249

[55] Innocenti M. New insights into the formation and the function of lamellipodia and ruffles in mesenchymal cell migration. Cell Adh Migr. 2018;12(5):401–16. 29513145 10.1080/19336918.2018.1448352 PMC6363039

[56] Mattila PK, Lappalainen P. Filopodia: molecular architecture and cellular functions. Nat Rev Mol Cell Biol. 2008;9(6):446–54. 18464790 10.1038/nrm2406

[57] Linder S, Cervero P, Eddy R, Condeelis J. Mechanisms and roles of podosomes and invadopodia. Nat Rev Mol Cell Biol. 2023;24(2):86–106. 36104625 10.1038/s41580-022-00530-6

[58] Dalle-Donne I, Rossi R, Giustarini D, Gagliano N, Lusini L, Milzani A, et al. Actin carbonylation: from a simple marker of protein oxidation to relevant signs of severe functional impairment. Free Radic Biol Med. 2001;31(9):1075–83. 11677040 10.1016/s0891-5849(01)00690-6

[59] Felding-Habermann B, O’Toole TE, Smith JW, Fransvea E, Ruggeri ZM, Ginsberg MH, Hughes PE, Pampori N, Shattil SJ, Saven A, Mueller BM. Integrin activation controls metastasis in human breast cancer. Proc Natl Acad Sci U S A. 2001;98(4):1853–8. 11172040 10.1073/pnas.98.4.1853 PMC29346

[60] Brooks PC, Clark RA, Cheresh DA. Requirement of vascular integrin alpha v beta 3 for angiogenesis. Science. 1994;264(5158):569–711. 7512751 10.1126/science.7512751

[61] Voura EB, Ramjeesingh RA, Montgomery AM, Siu CH. Involvement of integrin alpha(v)beta(3) and cell adhesion molecule L1 in transendothelial migration of melanoma cells. Mol Biol Cell. 2001;12(9):2699–710. 11553709 10.1091/mbc.12.9.2699 PMC59705

[62] Swiatkowska M, Szymański J, Padula G, Cierniewski CS. Interaction and functional association of protein disulfide isomerase with alphaVbeta3 integrin on endothelial cells. FEBS J. 2008;275(8):1813–23. 18331351 10.1111/j.1742-4658.2008.06339.x

[63] Schuler MH, Lewandowska A, Caprio GD, Skillern W, Upadhyayula S, Kirchhausen T, Shaw JM, Cunniff B. Miro1-mediated mitochondrial positioning shapes intracellular energy gradients required for cell migration. Mol Biol Cell. 2017;28(16):2159–69. 28615318 10.1091/mbc.E16-10-0741 PMC5531732

[64] Caino MC, Ghosh JC, Chae YC, Vaira V, Rivadeneira DB, Faversani A, Rampini R, Kossenkov AV, Aird KM, Zhang R, Webster MR, Weeraratna AT, Bosari S, Languino LR, Altieri DC. PI3K therapy reprograms mitochondrial trafficking to fuel tumor cell invasion. Proc Natl Acad Sci U S A. 2015;112(28):8638–43. 26124089 10.1073/pnas.1500722112 PMC4507184

[65] Rennick JJ, Johnston APR, Parton RG. Key principles and methods for studying the endocytosis of biological and nanoparticle therapeutics. Nat Nanotechnol. 2021;16(3):266–76. 33712737 10.1038/s41565-021-00858-8

[66] Yang C, Colosi P, Hugelier S, Zabezhinsky D, Lakadamyali M, Svitkina T. Actin polymerization promotes invagination of flat clathrin-coated lattices in mammalian cells by pushing at lattice edges. Nat Commun. 2022;13(1):6127. 36253374 10.1038/s41467-022-33852-2 PMC9576739

[67] Matthaeus C, Taraska JW. Energy and dynamics of Caveolae trafficking. Front Cell Dev Biol. 2020;8:614472. 33692993 10.3389/fcell.2020.614472 PMC7939723

[68] Doherty GJ, McMahon HT. Mechanisms of endocytosis. Annu Rev Biochem. 2009;78:857–902. 19317650 10.1146/annurev.biochem.78.081307.110540

[69] Ehrlich M, Boll W, Van Oijen A, Hariharan R, Chandran K, Nibert ML, et al. Endocytosis by random initiation and stabilization of clathrin-coated pits. Cell. 2004;118(5):591–605. 15339664 10.1016/j.cell.2004.08.017

[70] Carver LA, Schnitzer JE. Caveolae: mining little caves for new cancer targets. Nat Rev Cancer. 2003;3(8):571–81. 12894245 10.1038/nrc1146

[71] Neikirk K, Vue Z, Katti P, Rodriguez BI, Omer S, Shao J, et al. Systematic transmission electron microscopy-based identification and 3D reconstruction of cellular degradation machinery. Adv Biol (Weinh). 2023;7(6):e2200221. 10.1002/adbi.202200221 PMC13150769 36869426

[72] Cuervo AM, Dice JF. When lysosomes get old. Exp Gerontol. 2000;35(2):119–31. 10767573 10.1016/s0531-5565(00)00075-9

[73] Klionsky DJ, Eskelinen EL. Autophagosomes, phagosomes, autolysosomes, phagolysosomes, autophagolysosomes… wait, I’m confused. Autophagy. 2014;10(4):549–51. 24657946 10.4161/auto.28448 PMC4091142

[74] Buratta S, Tancini B, Sagini K, Delo F, Chiaradia E, Urbanelli L, et al. Lysosomal exocytosis, exosome release and secretory autophagy: the autophagic- and endo-lysosomal systems go extracellular. Int J Mol Sci. 2020;21(7):2576. 10.3390/ijms21072576 PMC7178086 32276321

[75] Blott EJ, Griffiths GM. Secretory lysosomes. Nat Rev Mol Cell Biol. 2002;3(2):122–31. 11836514 10.1038/nrm732

[76] Barral DC, Staiano L, Guimas Almeida C, Cutler DF, Eden ER, Futter CE, Galione A, Marques ARA, Medina DL, Napolitano G, Settembre C, Vieira OV, Aerts JMFG, Atakpa-Adaji P, Bruno G, Capuozzo A, De Leonibus E, Di Malta C, Escrevente C, Esposito A, Grumati P, Hall MJ, Teodoro RO, Lopes SS, Luzio JP, Monfregola J, Montefusco S, Platt FM, Polishchuck R, De Risi M, Sambri I, Soldati C, Seabra MC. Current methods to analyze lysosome morphology, positioning, motility and function. Traffic. 2022;23(5):238–69. 35343629 10.1111/tra.12839 PMC9323414

[77] Jeppesen DK, Zhang Q, Franklin JL, Coffey RJ. Extracellular vesicles and nanoparticles: emerging complexities. Trends Cell Biol. 2023;33(8):667–81. 36737375 10.1016/j.tcb.2023.01.002 PMC10363204

[78] Yu L, Chen Y, Tooze SA. Autophagy pathway: cellular and molecular mechanisms. Autophagy. 2018;14(2):207–15. 28933638 10.1080/15548627.2017.1378838 PMC5902171

[79] Piletic K, Alsaleh G, Simon AK. Autophagy orchestrates the crosstalk between cells and organs. EMBO Rep. 2023;24(9):e57289. 37465980 10.15252/embr.202357289 PMC10481659

[80] Machado ER, Annunziata I, van de Vlekkert D, Grosveld GC, d’Azzo A. Lysosomes and Cancer Progression: A Malignant Liaison. Front Cell Dev Biol. 2021;9:642494. 33718382 10.3389/fcell.2021.642494 PMC7952443

[81] Bustos SO, Leal Santos N, Chammas R, Andrade LNS. Secretory autophagy forges a therapy resistant microenvironment in melanoma. Cancers (Basel). 2022;14(1):234. 10.3390/cancers14010234 PMC8749976 35008395

[82] Machado E, White-Gilbertson S, van de Vlekkert D, Janke L, Moshiach S, Campos Y, Finkelstein D, Gomero E, Mosca R, Qiu X, Morton CL, Annunziata I, d’Azzo A. Regulated lysosomal exocytosis mediates cancer progression. Sci Adv. 2015;1(11):e1500603. 26824057 10.1126/sciadv.1500603 PMC4730843

[83] Li X, Zhao H. Targeting secretory autophagy in solid cancers: mechanisms, immune regulation and clinical insights. Exp Hematol Oncol. 2025;14(1):12. 39893499 10.1186/s40164-025-00603-0 PMC11786567

[84] Welch DR, Hurst DR. Defining the hallmarks of metastasis. Cancer Res. 2019;79(12):3011–27. 31053634 10.1158/0008-5472.CAN-19-0458 PMC6571042

[85] Pelzer KM, Cheng L, Curtiss LA. Effects of functional groups in redox-active organic molecules: a high-throughput screening approach. J Phys Chem C. 2017;121(1):237–45.

[86] Hauser AK, Mitov MI, Daley EF, McGarry RC, Anderson KW, Hilt JZ. Targeted iron oxide nanoparticles for the enhancement of radiation therapy. Biomaterials. 2016;105:127–35. 27521615 10.1016/j.biomaterials.2016.07.032 PMC5321199

[87] Asati A, Santra S, Kaittanis C, Perez JM. Surface-charge-dependent cell localization and cytotoxicity of cerium oxide nanoparticles. ACS Nano. 2010;4(9):5321–31. 20690607 10.1021/nn100816s PMC2947560

[88] Lunov O, Syrovets T, Röcker C, Tron K, Nienhaus GU, Rasche V, Mailänder V, Landfester K, Simmet T. Lysosomal degradation of the carboxydextran shell of coated superparamagnetic iron oxide nanoparticles and the fate of professional phagocytes. Biomaterials. 2010;31(34):9015–22. 20739059 10.1016/j.biomaterials.2010.08.003

[89] Wu H-Y, Chung M-C, Wang C-C, Huang C-H, Liang H-J, Jan T-R. Iron oxide nanoparticles suppress the production of IL-1beta via the secretory lysosomal pathway in murine microglial cells. Part Fibre Toxicol. 2013;10(1):46. 24047432 10.1186/1743-8977-10-46 PMC3851143

[90] Beaune G, Nagarajan U, Brochard-Wyart F, Winnik FM. Polymeric nanoparticles limit the collective migration of cellular aggregates. Langmuir. 2019;35(23):7396–404. 29975543 10.1021/acs.langmuir.8b01736 PMC6562752

[91] Mulens-Arias V, Rojas JM, Pérez-Yagüe S, Morales MP, Barber DF. Polyethylenimine-coated SPION exhibits potential intrinsic anti-metastatic properties inhibiting migration and invasion of pancreatic tumor cells. J Control Release. 2015;216:78–92. 26264831 10.1016/j.jconrel.2015.08.009

[92] Mulens-Arias VRJ, Pérez-Yagüe S, Morales MP, Barber DF. Polyethylenimine-coated SPIONs trigger macrophage activation through TLR-4 signaling and ROS production and modulate podosome dynamics. Biomaterials. 2015;52:494–506. 25818455 10.1016/j.biomaterials.2015.02.068

[93] Mulens-Arias V, Rojas JM, Sanz-Ortega L, Portilla Y, Pérez-Yagüe S, Barber DF. Polyethylenimine-coated superparamagnetic iron oxide nanoparticles impair in vitro and in vivo angiogenesis. Nanomedicine. 2019;21:102063. 31326525 10.1016/j.nano.2019.102063

[94] Rojas JM, Sanz-Ortega L, Mulens-Arias V, Gutiérrez L, Pérez-Yagüe S, Barber DF. Superparamagnetic iron oxide nanoparticle uptake alters M2 macrophage phenotype, iron metabolism, migration and invasion. Nanomedicine. 2016;12(4):1127–38. 26733263 10.1016/j.nano.2015.11.020

[95] Li X, Wei Z, Lv H, Wu L, Cui Y, Yao H, Li J, Zhang H, Yang B, Jiang J. Iron oxide nanoparticles promote the migration of mesenchymal stem cells to injury sites. Int J Nanomed. 2019;14:573–89. 10.2147/IJN.S184920 PMC6336032 30666115

[96] Ali MRK, Wu Y, Ghosh D, Do BH, Chen K, Dawson MR, et al. Nuclear membrane-targeted gold nanoparticles inhibit cancer cell migration and invasion. ACS Nano. 2017;11(4):3716–26. 28333438 10.1021/acsnano.6b08345 PMC5519406

[97] Sheykhzadeh S, Luo M, Peng B, White J, Abdalla Y, Tang T, et al. Transferrin-targeted porous silicon nanoparticles reduce glioblastoma cell migration across tight extracellular space. Sci Rep. 2020;10(1):2320. 32047170 10.1038/s41598-020-59146-5 PMC7012928

[98] Dalle-Donne I, Milzani A, Colombo R. H2O2-treated actin: assembly and polymer interactions with cross-linking proteins. Biophys J. 1995;69(6):2710–9. 8599677 10.1016/S0006-3495(95)80142-6 PMC1236508

[99] Dalle-Donne I, Giustarini D, Rossi R, Colombo R, Milzani A. Reversible S-glutathionylation of Cys 374 regulates actin filament formation by inducing structural changes in the actin molecule. Free Radic Biol Med. 2003;34(1):23–32. 12498976 10.1016/s0891-5849(02)01182-6

[100] Frémont S, Hammich H, Bai J, Wioland H, Klinkert K, Rocancourt M, et al. Oxidation of F-actin controls the terminal steps of cytokinesis. Nat Commun. 2017;8:14528. 28230050 10.1038/ncomms14528 PMC5331220

[101] Heo J, Campbell SL. Mechanism of redox-mediated guanine nucleotide exchange on redox-active Rho GTPases. J Biol Chem. 2005;280(35):31003–10. 15994296 10.1074/jbc.M504768200

[102] Fiaschi T, Cozzi G, Raugei G, Formigli L, Ramponi G, Chiarugi P. Redox regulation of beta-actin during integrin-mediated cell adhesion. J Biol Chem. 2006;281(32):22983–91. 16757472 10.1074/jbc.M603040200

[103] Chugh P, Paluch EK. The actin cortex at a glance. J Cell Sci. 2018;131(14):jcs186254. 10.1242/jcs.186254 PMC6080608 30026344

[104] Sliogeryte K, Thorpe SD, Lee DA, Botto L, Knight MM. Stem cell differentiation increases membrane-actin adhesion regulating cell blebability, migration and mechanics. Sci Rep. 2014;4:7307. 25471686 10.1038/srep07307 PMC4255193

[105] Kalukula Y, Luciano M, Simanov G, Charras G, Brückner DB, Gabriele S. The actin cortex acts as a mechanical memory of morphology in confined migrating cells. Nat Phys. 2025;21(9):1451–61.

[106] Henze A, Raila J, Scholze A, Zidek W, Tepel M, Schweigert FJ. Does N-acetylcysteine modulate post-translational modifications of transthyretin in hemodialysis patients? Antioxid Redox Signal. 2013;19(11):1166–72. 23249342 10.1089/ars.2012.5125

[107] Soenen SJ, Nuytten N, De Meyer SF, De Smedt SC, De Cuyper M. High intracellular iron oxide nanoparticle concentrations affect cellular cytoskeleton and focal adhesion kinase-mediated signaling. Small. 2010;6(7):832–42. 20213651 10.1002/smll.200902084

[108] Chen Y, Hou S. Recent progress in the effect of magnetic iron oxide nanoparticles on cells and extracellular vesicles. Cell Death Discov. 2023;9(1):195. 37380637 10.1038/s41420-023-01490-2 PMC10307851

[109] Chiaradia E, Tancini B, Emiliani C, Delo F, Pellegrino RM, Tognoloni A, et al. Extracellular vesicles under oxidative stress conditions: biological properties and physiological roles. Cells. 2021;10(7):1763. 10.3390/cells10071763 PMC8306565 34359933

[110] Benedikter BJ, Weseler AR, Wouters EFM, Savelkoul PHM, Rohde GGU, Stassen FRM. Redox-dependent thiol modifications: implications for the release of extracellular vesicles. Cell Mol Life Sci. 2018;75(13):2321–37. 29594387 10.1007/s00018-018-2806-z PMC5986851

[111] Vats S, Galli T. Role of SNAREs in unconventional secretion-focus on the VAMP7-dependent secretion. Front Cell Dev Biol. 2022;10:884020. 35784483 10.3389/fcell.2022.884020 PMC9244844

[112] Südhof TC, Rothman JE. Membrane fusion: grappling with SNARE and SM proteins. Science. 2009;323(5913):474–7. 19164740 10.1126/science.1161748 PMC3736821

[113] Hutagalung AH, Novick PJ. Role of Rab GTPases in membrane traffic and cell physiology. Physiol Rev. 2011;91(1):119–49. 21248164 10.1152/physrev.00059.2009 PMC3710122

[114] Chaineau M, Danglot L, Galli T. Multiple roles of the vesicular-SNARE TI-VAMP in post-Golgi and endosomal trafficking. FEBS Lett. 2009;583(23):3817–26. 19837067 10.1016/j.febslet.2009.10.026

[115] Proux-Gillardeaux V, Raposo G, Irinopoulou T, Galli T. Expression of the longin domain of TI-VAMP impairs lysosomal secretion and epithelial cell migration. Biol Cell. 2007;99(5):261–71. 17288539 10.1042/BC20060097

[116] Verderio C, Cagnoli C, Bergami M, Francolini M, Schenk U, Colombo A, Riganti L, Frassoni C, Zuccaro E, Danglot L, Wilhelm C, Galli T, Canossa M, Matteoli M. TI-VAMP/VAMP7 is the SNARE of secretory lysosomes contributing to ATP secretion from astrocytes. Biol Cell. 2012;104(4):213–28. 22188132 10.1111/boc.201100070

[117] Escrevente C, Bento-Lopes L, Ramalho JS, Barral DC. Rab11 is required for lysosome exocytosis through the interaction with Rab3a, Sec15 and GRAB. J Cell Sci. 2021;134(11):jcs246694. 10.1242/jcs.246694 PMC8214760 34100549

[118] Ly T, Pickard B, Pandey A, Yap M, Opara J, Arnold L, et al. TRIM16 mediates secretory autophagy in head and neck cancer-associated fibroblasts. Autophagy. 2025;21(11):2473–96. 40383937 10.1080/15548627.2025.2508064 PMC12362711

[119] van Meteren N, Lagadic-Gossmann D, Chevanne M, Gallais I, Gobart D, Burel A, et al. Polycyclic aromatic hydrocarbons can trigger hepatocyte release of extracellular vesicles by various mechanisms of action depending on their affinity for the aryl hydrocarbon receptor. Toxicol Sci. 2019;171(2):443–62. 31368503 10.1093/toxsci/kfz157

[120] Zhang W, Liu R, Chen Y, Wang M, Du J. Crosstalk between Oxidative Stress and Exosomes. Oxid Med Cell Longev. 2022;2022:3553617. 36082080 10.1155/2022/3553617 PMC9448575

[121] Ohnishi Y, Tsuji D, Itoh K. Oxidative stress impairs autophagy via inhibition of lysosomal transport of VAMP8. Biol Pharm Bull. 2022;45(11):1609–15. 36328496 10.1248/bpb.b22-00131

[122] Shelke GV, Williamson CD, Jarnik M, Bonifacino JS. Inhibition of endolysosome fusion increases exosome secretion. J Cell Biol. 2023 ;222 (6): e202209084. 10.1083/jcb.202209084 PMC10202829 37213076

[123] Marzano M, Bou-Dargham MJ, Cone AS, York S, Helsper S, Grant SC, et al. Biogenesis of extracellular vesicles produced from human-stem-cell-derived cortical spheroids exposed to iron oxides. ACS Biomater Sci Eng. 2021;7(3):1111–22. 10.1021/acsbiomaterials.0c01286 PMC8185622 33525864

[124] Kutchy NA, Ma R, Liu Y, Buch S, Hu G. Extracellular vesicle-mediated delivery of ultrasmall superparamagnetic iron oxide nanoparticles to mice brain. Front Pharmacol. 2022;13:819516. 35462907 10.3389/fphar.2022.819516 PMC9022024

[125] Vingtdeux V, Hamdane M, Loyens A, Gelé P, Drobeck H, Bégard S, Galas MC, Delacourte A, Beauvillain JC, Buée L, Sergeant N. Alkalizing drugs induce accumulation of amyloid precursor protein by-products in luminal vesicles of multivesicular bodies. J Biol Chem. 2007;282(25):18197–205. 17468104 10.1074/jbc.M609475200

[126] Alvarez-Erviti L, Seow Y, Schapira AH, Gardiner C, Sargent IL, Wood MJ, Cooper JM. Lysosomal dysfunction increases exosome-mediated alpha-synuclein release and transmission. Neurobiol Dis. 2011;42(3):360–7. 21303699 10.1016/j.nbd.2011.01.029 PMC3107939

[127] Strauss K, Goebel C, Runz H, Möbius W, Weiss S, Feussner I, Simons M, Schneider A. Exosome secretion ameliorates lysosomal storage of cholesterol in Niemann-Pick type C disease. J Biol Chem. 2010;285(34):26279–88. 20554533 10.1074/jbc.M110.134775 PMC2924046

[128] Eitan E, Suire C, Zhang S, Mattson MP. Impact of lysosome status on extracellular vesicle content and release. Ageing Res Rev. 2016;32:65–74. 27238186 10.1016/j.arr.2016.05.001 PMC5154730

[129] Li P, Bademosi AT, Luo J, Meunier FA. Actin remodeling in regulated exocytosis: toward a mesoscopic view. Trends Cell Biol. 2018;28(9):685–97. 29759816 10.1016/j.tcb.2018.04.004

[130] Tabb JS, Molyneaux BJ, Cohen DL, Kuznetsov SA, Langford GM. Transport of ER vesicles on actin filaments in neurons by myosin V. J Cell Sci. 1998;111(Pt 21):3221–34. 9763516 10.1242/jcs.111.21.3221

[131] Gao L, Zhuang J, Nie L, Zhang J, Zhang Y, Gu N, Wang T, Feng J, Yang D, Perrett S, Yan X. Intrinsic peroxidase-like activity of ferromagnetic nanoparticles. Nat Nanotechnol. 2007;2(9):577–83. 18654371 10.1038/nnano.2007.260

[132] Chen Z, Yin JJ, Zhou YT, Zhang Y, Song L, Song M, Hu S, Gu N. Dual enzyme-like activities of iron oxide nanoparticles and their implication for diminishing cytotoxicity. ACS Nano. 2012;6(5):4001–12. 22533614 10.1021/nn300291r

[133] Zhitomirsky B, Assaraf YG. Lysosomal accumulation of anticancer drugs triggers lysosomal exocytosis. Oncotarget. 2017;8(28):45117–32. 28187461 10.18632/oncotarget.15155 PMC5542171

[134] Uzhytchak M, Smolková B, Lunova M, Jirsa M, Frtús A, Kubinová Š, et al. Iron oxide nanoparticle-induced autophagic flux is regulated by interplay between p53-mTOR axis and Bcl-2 signaling in hepatic cells. Cells. 2020;9(4): 1015. 10.3390/cells9041015 PMC7226334 32325714

[135] Jin R, Liu L, Zhu W, Li D, Yang L, Duan J, et al. Iron oxide nanoparticles promote macrophage autophagy and inflammatory response through activation of toll-like receptor-4 signaling. Biomaterials. 2019;203:23–30. 30851490 10.1016/j.biomaterials.2019.02.026

[136] Nie Q, Chen W, Zhang T, Ye S, Ren Z, Zhang P, et al. Iron oxide nanoparticles induce ferroptosis via the autophagic pathway by synergistic bundling with paclitaxel. Mol Med Rep. 2023;28(4):198. 37681444 10.3892/mmr.2023.13085 PMC10510030

[137] Xie Y, Jiang J, Tang Q, Zou H, Zhao X, Liu H, et al. Iron Oxide Nanoparticles as Autophagy Intervention Agents Suppress Hepatoma Growth by Enhancing Tumoricidal Autophagy. Adv Sci (Weinh). 2020;7(16):1903323. 32832347 10.1002/advs.201903323 PMC7435245

[138] Dupont N, Jiang S, Pilli M, Ornatowski W, Bhattacharya D, Deretic V. Autophagy-based unconventional secretory pathway for extracellular delivery of IL-1β. EMBO J. 2011;30(23):4701–11. 22068051 10.1038/emboj.2011.398 PMC3243609

[139] Miller S, Tavshanjian B, Oleksy A, Perisic O, Houseman BT, Shokat KM, Williams RL. Shaping development of autophagy inhibitors with the structure of the lipid kinase Vps34. Science. 2010;327(5973):1638–42. 20339072 10.1126/science.1184429 PMC2860105

[140] Akin D, Wang SK, Habibzadegah-Tari P, Law B, Ostrov D, Li M, et al. A novel ATG4B antagonist inhibits autophagy and has a negative impact on osteosarcoma tumors. Autophagy. 2014;10(11):2021–35. 10.4161/auto.32229 PMC4502682 25483883

[141] Rahman MA, Engelsen AST, Sarowar S, Bindesbøll C, Birkeland E, Goplen D, et al. Bortezomib abrogates temozolomide-induced autophagic flux through an ATG5 dependent pathway. Front Cell Dev Biol. 2022;10:1022191. 36619857 10.3389/fcell.2022.1022191 PMC9814514

[142] Egan DF, Chun MG, Vamos M, Zou H, Rong J, Miller CJ, Lou HJ, Raveendra-Panickar D, Yang CC, Sheffler DJ, Teriete P, Asara JM, Turk BE, Cosford ND, Shaw RJ. Small Molecule Inhibition of the Autophagy Kinase ULK1 and Identification of ULK1 Substrates. Mol Cell. 2015;59(2):285–97. 26118643 10.1016/j.molcel.2015.05.031 PMC4530630

[143] Eng CH, Wang Z, Tkach D, Toral-Barza L, Ugwonali S, Liu S, et al. Macroautophagy is dispensable for growth of KRAS mutant tumors and chloroquine efficacy. Proc Natl Acad Sci U S A. 2016;113(1):182–7. 26677873 10.1073/pnas.1515617113 PMC4711870

[144] Feng Q, Zhang C, Lum D, Druso JE, Blank B, Wilson KF, et al. A class of extracellular vesicles from breast cancer cells activates VEGF receptors and tumour angiogenesis. Nat Commun. 2017;8:14450. 28205552 10.1038/ncomms14450 PMC5316898

[145] An M, Kim H, Moon JM, Ko HS, Clayton P, Lim YH. Enzyme-treated *Zizania latifolia* ethanol extract protects from UVA irradiation-induced wrinkle formation via inhibition of lysosome exocytosis and reactive oxygen species generation. Antioxidants. 2020;9(10):912. 10.3390/antiox9100912 PMC7600157 32987843

[146] Wen T, Du L, Chen B, Yan D, Yang A, Liu J, et al. Iron oxide nanoparticles induce reversible endothelial-to-mesenchymal transition in vascular endothelial cells at acutely non-cytotoxic concentrations. Part Fibre Toxicol. 2019;16(1):30. 31300057 10.1186/s12989-019-0314-4 PMC6626375

[147] Bartczak D, Muskens OL, Nitti S, Millar TM, Kanaras AG. Nanoparticles for inhibition of in vitro tumour angiogenesis: synergistic actions of ligand function and laser irradiation. Biomater Sci. 2015;3(5):733–41. 26222592 10.1039/c5bm00053j

[148] Zhang Y, Xiong X, Huai Y, Dey A, Hossen MN, Roy RV, et al. Gold nanoparticles disrupt tumor microenvironment - endothelial cell cross talk to inhibit angiogenic phenotypes in vitro. Bioconjug Chem. 2019;30(6):1724–33. 31067032 10.1021/acs.bioconjchem.9b00262 PMC6939887

[149] Niethammer P, Grabher C, Look AT, Mitchison TJ. A tissue-scale gradient of hydrogen peroxide mediates rapid wound detection in zebrafish. Nature. 2009;459(7249):996–9. 19494811 10.1038/nature08119 PMC2803098

[150] Yang E, Wang X, Gong Z, Yu M, Wu H, Zhang D. Exosome-mediated metabolic reprogramming: the emerging role in tumor microenvironment remodeling and its influence on cancer progression. Signal Transduct Target Ther. 2020;5(1):242. 33077737 10.1038/s41392-020-00359-5 PMC7572387

[151] Polónia B, Xavier CPR, Kopecka J, Riganti C, Vasconcelos MH. The role of extracellular vesicles in glycolytic and lipid metabolic reprogramming of cancer cells: consequences for drug resistance. Cytokine Growth Factor Rev. 2023;73:150–62. 37225643 10.1016/j.cytogfr.2023.05.001

[152] Wan L, Xia T, Du Y, Liu J, Xie Y, Zhang Y, et al. Exosomes from activated hepatic stellate cells contain GLUT1 and PKM2: a role for exosomes in metabolic switch of liver nonparenchymal cells. FASEB J. 2019;33(7):8530–42. 30970216 10.1096/fj.201802675R

[153] Suzuki K, Yoshino D. Proliferation-related activity in endothelial cells is enhanced by micropower plasma. Biomed Res Int. 2016;2016:4651265. 28058258 10.1155/2016/4651265 PMC5183802

