# Supplementary material for: Oxidative stress induced by DMSA-IONPs impairs breast cancer cell migration and paracrine cell communication
Source: J Nanobiotechnology. 2026 Apr 9;24:483. doi: 10.1186/s12951-026-04412-3 (PMC13200410; doi:10.1186/s12951-026-04412-3)
Supplement: Supplementary file 1 — Supplementary Material 1 [file 12951_2026_4412_MOESM1_ESM.pdf]

Oxidative stress induced by DMSA-IONPs impairs breast cancer cell migration and paracrine cell communication

Neus Daviu[1,2], Carla Graciano-Casero[1] and Domingo F. Barber[1*]

[1]Department of Immunology, Oncology and Nanobiomedicine Initiative, Centro Nacional de Biotecnología (CNB-CSIC), Darwin 3, 28049 Madrid, Spain.

[2]Faculty of Experimental Sciences, Francisco de Vitoria University (UFV), Ctra. Pozuelo-Majadahonda Km 1,800, 28223 Pozuelo de Alarcón, Spain

***Corresponding and Senior Authors:** *E-mail: dfbarber@cnb.csic.es (D.F.B.)*

Supplemental Materials and Methods

The effect of the antioxidant NAC on DMSA-IONP internalization, ROS production and cell migration

To assess if the antioxidant N-Acetylcysteine (NAC) interferes with DMSA-IONP internalization, ROS production and cell migration, ICP-OES, DHR staining and wound healing assays were performed in the presence of NAC. As such, MDA-MB-231 cells were left untreated, treated with DMSA-IONPs for 24 h or treated for 2 h with NAC (5mM) prior to exposure to DMSA-IONPs for 24h in the continued presence of NAC. Subsequently, the cell's iron content was determined by ICP-OES, the production of ROS was assessed by flow cytometry of DHR stained cells, and cell migration was evaluated in wound healing assays.

Integrin $\alpha v\beta_3$ expression by flow cytometry

The expression of integrin $\alpha v\beta_3$ by MDA-MB-231 cells was assessed by flow cytometry. MDA-MB-231 cells were plated in a 12-well plate and cultured for 24 h, after which they were left untreated or treated with DMSA-IONPs for 24 h. Subsequently, the cells were recovered with a scraper and stained for 1 h at room temperature (RT) with an antibody against $\alpha v\beta_3$ integrin (23C6, sc-7312, Santa Cruz Biotechnology) diluted 1:40 in 100 µl of PBS. Antibody binding was then detected with the secondary anti-mouse IgG1-PE antibody (1070-09, Beckman Coulter) for 30 min at RT. As negative controls the cells were stained for 30 min at RT with an isotypic mouse IgG1-PE antibody (Pharmingen, 556029). After staining, the cells were washed and re-suspended in 300 µl of PBS to determine the presence of integrin $\alpha v\beta_3$ on a Cytomics FC500 cytometer using FL3 (Ex/Em = 488/620 nm). For each sample, 100,000 events per condition were acquired and analyzed using FlowJo analysis software.

Flow cytometry to assess intracellular acidification using Lysotracker staining

The acidification of lysosomes was determined by flow cytometry in cells labelled with Lysotracker Green DND-26. MDA-MB-231 cells were plated in a 12-well plate for 24 h and they were then left untreated or treated with DMSA-IONPs for 24 h. Subsequently the cells were stained for 2 h with Lysotracker Green diluted 1:2,000 in culture medium. After staining, the cells were recovered, re-suspended in 300 μl of PBS and acidification was measured on a Cytomics FC500 cytometer using FL1 (Ex/Em = 488/525 nm). Lysotracker green, evaluating the mean fluorescence intensity with FlowJo software.

Extracellular ROS and the pH of MDA-MB-231 cell conditioned medium (CM)

To assess the modification of ROS and pH in the extracellular medium of untreated or DMSA-IONP treated MDA-MB-231 cells, conditioned medium (CM) was recovered as indicated in the Materials and Methods. ROS production was then determined by staining the medium 1:500 with DHR and measuring the fluorescence in a SpectraMAX Gemini plate reader (Em/Ex = 488/525 nm). The pH of the medium was determined with pH-indicator strips (MERCK).

SVEC4-10 cell viability

To determine if the DMSA-IONPs, or the CM of untreated (CM-Control) or DMSA-IONP treated (CM-DMSA-IONP) MDA-MB-231 cells affected SVEC4-10 cell viability, different assays were performed. The induction of cell death was analyzed with the Annexin V-FITC kit (10010-02, SouthernBiotech) and measured by flow cytometry. Cells were seeded in 12-well plates for 24 h, and they were then incubated with DMSA-IONPs (60 and 125 μgFe/ml), or with CM-Control or CM-DMSA-IONPs for 24 h. Subsequently, the cells were washed with PBS, recovered by trypsin digestion, centrifuged, and the pellet was recovered and resuspended in 100 μl Binding Buffer with

5 μl Annexin V-FITC for 10 min on ice. After this time, 250 μl of Binding Buffer and 3 μl of Propidium Iodide (PI) was added to each sample, and the Annexin V-FITC fluorescence was analysed on a Cytomics FC500 cytometer using FL1 (ex/em = 488/525 nm) while PI was assessed using FL3 (ex/em = 488/620). For each sample, 50,000 events were acquired and the live/dead populations were analysed with FlowJo Software.

PrestoBlue™ (ThermoFisher Scientific) reagent was used to analyze the metabolic activity of CM-treated SVEC4-10 cells. Cells were plated in 96-well plates and left untreated or treated with CM-Control or CM-DMSA-IONP for 24h. Subsequently, the PrestoBlue reagent was added (1:10 medium) and the cells were incubated for 2 h for color switching. The PrestoBlue fluorescence was then analyzed in a SpectraMAX Gemini plate reader (excitation 535, emission 590 nm).

Crystal Violet (V5265-500, Sigma-Aldrich) reagent was used to analyze the confluence of CM-treated SVEC4-10 cell cultures. The cells were plated in a 96-well plate and left untreated, or treated with CM-Control or CM-DMSA-IONP for 24h. Subsequently, the cells were washed twice with ice-cold Hank's Balanced Salt Solution (HBSS: 1402 5092, Gibco), fixed with ice-cold methanol for 15 min at RT and left in the wells to dry. After drying, the wells were stained with Crystal Violet 0.5% (V/V water) for 10 min and then washed with abundant water. Finally, the remaining Crystal Violet was dissolved with 10% Acetic Acid (V/V water) over 10 min and the absorbance was measured at 570 nm in a SpectraMAX Gemini plate reader.

Determination of VEGF, MMP2 and MMP9 extracellular content by ELISA

To study the presence of chemotactic chemokines in CM, the extracellular content of VEGF and MMP2 was analyzed by Human VEGF DuoSet ELISA (R&D systems, #DY293B-05) and Human MMP2 ELISA kit (R&D systems, #DY902), respectively. As

wash buffer a 0,05% Tween ® 20 in PBS solution was prepared; as block buffer and reagent diluent a 1% BSA in PBS solution was prepared and 0.2 μm filtered. Stop Solution 2N Sulfuric Acid (R&D systems, #DY994) was used as Stop solution and Substrate Reagent Pack (R&D systems, #DY999) was used as Substrate solution for assay procedure.

MDA-MB-231 cells were seeded in 12-well plates and after treatment with DMSA-IONPs (125 μg/ml) for 24 h, extracellular medium (CM-Control and CM-DMSA-IONP) was collected and centrifuged at 13,000 rpm (equal to 15,800 g) for 15 min at RT to collect the supernatant. Supernatant was used to assess extracellular content of VEGF, MMP2 and MMP9 by ELISA following manufacturer's instructions. Briefly, capture antibody (either VEGF, MMP2 or MMP9) was diluted to working concentration (1:200 in PBS), added into the 96 well microplate, and the plate was sealed and incubated ON at 4ºC. Following day, plate was washed thoroughly with Wash buffer and blocked for 1 h at RT with block buffer. After blocking, plate was washed thoroughly and either 100 μl of sample or VEGF and MMP2 standards were added into the plate. Plate was incubated 2 h at RT and after incubation washed thoroughly prior to adding 100 μl of Detection Antibody (1:200 in reagent diluent). Plate was incubated 2 h at RT and after incubation, washed thoroughly and 100 μl of Streptavidin-HRP-B solution was added to each well and incubated 20 min at RT. After incubation and washings, 100 μl substrate solution was added to each well, incubated for 20 min and 50 μl of Stop solution was added prior to measuring optical density. Optical density was read at a microplate reader Multiskan GO (Thermo Scientific) set to 450 nm and 540 nm. Optical density reading at 540 nm were subtracted from those at 450 for wavelength correction of optical imperfections.

Supplemental Results

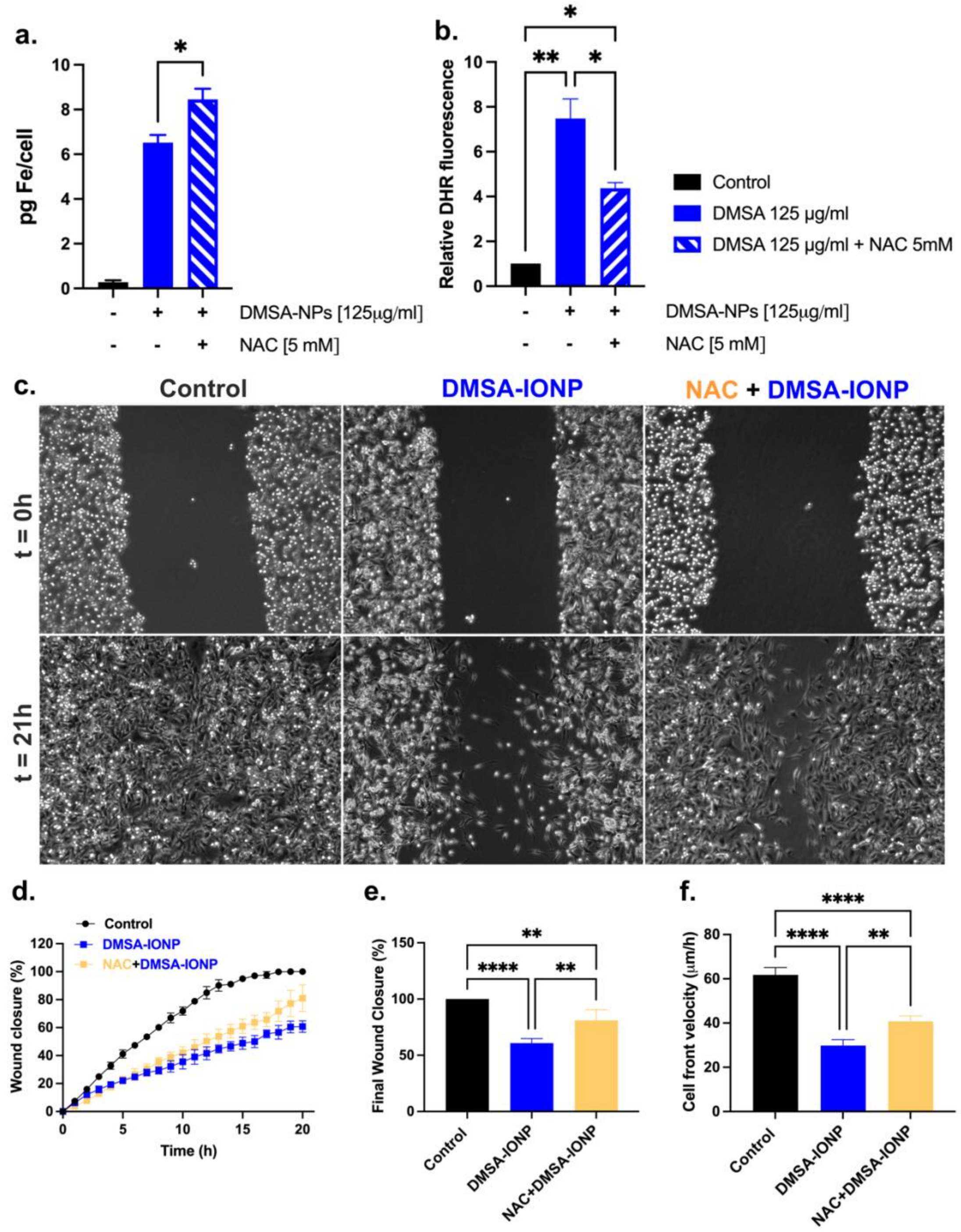

Figure S1. The antioxidant NAC counteracts the induction of ROS production by DMSA-IONPs and their effects on cell migration. a. The effect of NAC on DMSA-IONP internalization as measured by ICP-OES **b.** The effect of NAC on the production of ROS induced by DMSA-IONPs. **c.** The effect of NAC on collective MDA-MB-231 cell migration in a wound healing assay. MDA-MB-231 cells were left untreated (control), or treated for 24 h with DMSA-IONPs alone (125 µg/ml) or in the presence of NAC (5 mM). **d.** Relative wound closure over time. **e.** Relative total wound closure after 21 h. **f.** Cell front velocity over time. The data is the mean ± SD (n = 3), and an unpaired t-test and one-way ANOVA with Tukey's multiple

comparison test were used to assess the differences between untreated cells and those exposed to DMSA-IONPs in the presence or absence of NAC: *p <0.05, **p <0.01, ***p <0.001, **** p <0.0001.

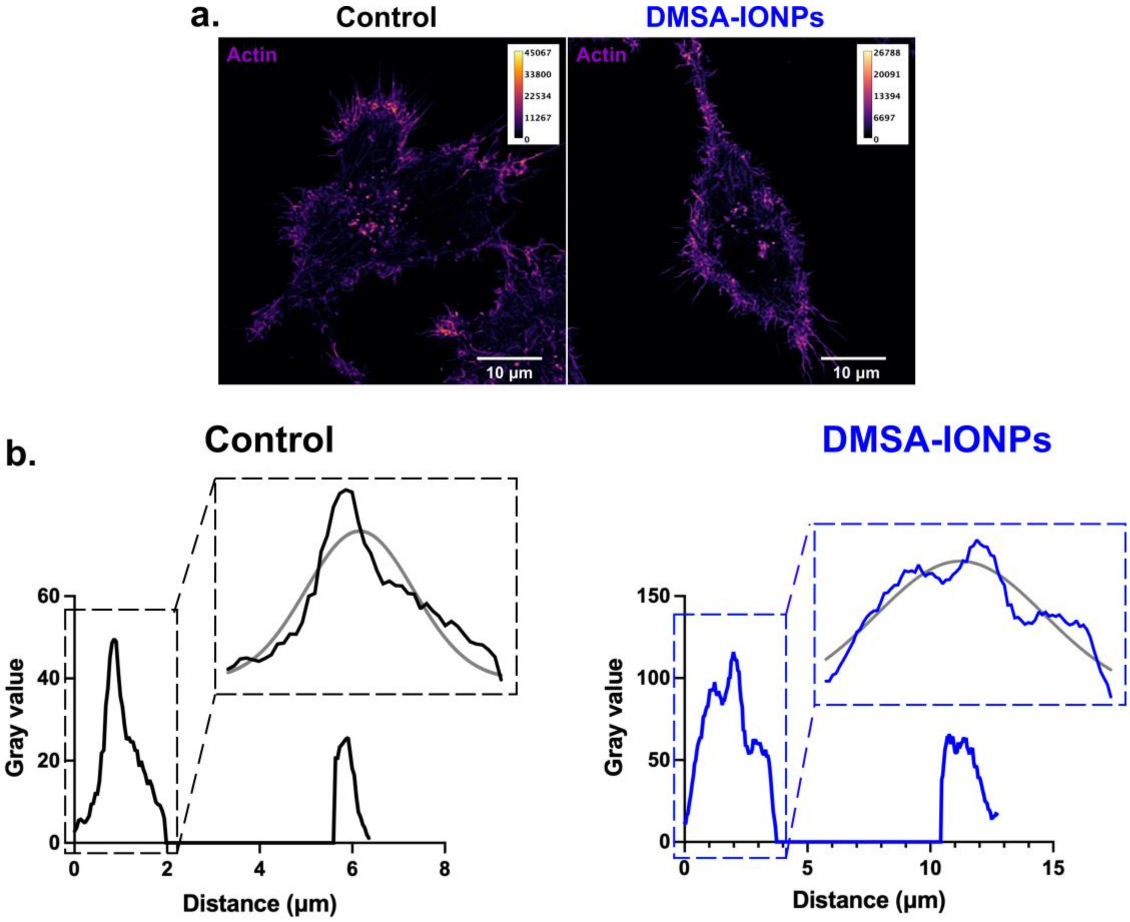

<u>Figure S2</u>. DMSA-IONP treatment affects the actin cytoskeleton in MDA-MB-231 cells. a. Heatmap intensity of actin labelled with phalloidin. **b.** Intensity plots of cortical F-actin thickness by measuring F-actin intensity pixels along a line drawn perpendicular to F-actin in different regions of cell. The black line corresponds to F-actin intensity in untreated cells and blue line corresponds to F-actin intensity in DMSA-IONP-treated cells. Plots were analyzed and adjusted fitting a Gaussian curve (grey line).

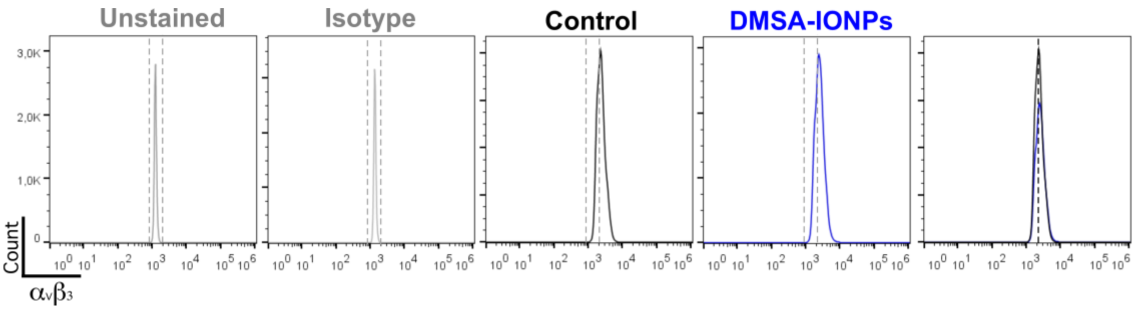

Figure S3. DMSA-IONP treatment does not alter membrane-bound integrin $\alpha_V\beta_3$. Flow cytometry histograms of integrin $\alpha_V\beta_3$ intensity.

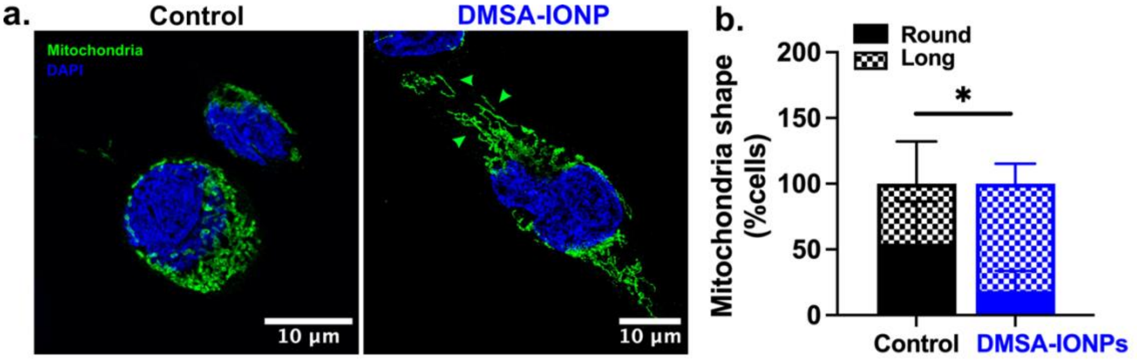

Figure S4. Mitochondria shape and disposition is altered by DMSA-IONPs in MDA-MB-231 cells. a. The disposition of mitochondria in untreated and DMSA-IONP treated cells visualized by immunofluorescence with the mitochondrial specific marker, TOM20: mitochondria stained with the TOM20 antibody, green; DAPI stained nucleus, blue. Scale bar: 10 µm. **b.** Quantification of the proportion of cells with round/spherical mitochondria (filled bars) or with long/elongated mitochondria (squared bars). The data is the mean ± SD (n > 200 cells) and a two-way ANOVA with a Sídák's multiple comparison test was used to assess the differences in mitochondria shape between untreated and treated cells: *p <0.05, **p <0.01, ***p <0.001, **** p <0.0001.

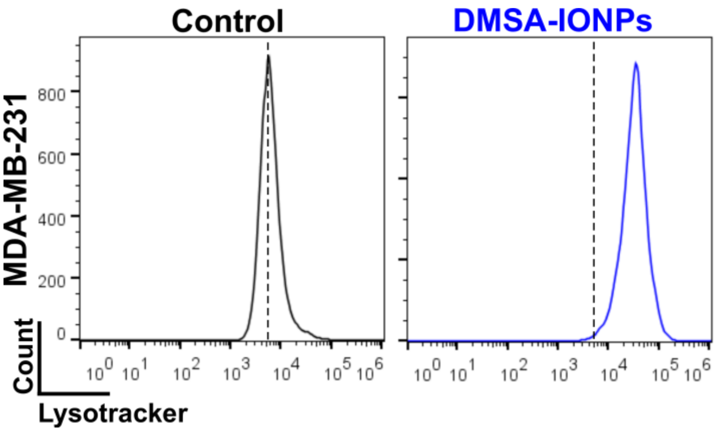

Figure S5. Intracellular acidification triggered by DMSA-IONPs assessed by flow cytometry of Lysotracker DND26 stained cells. Dashed line shows the mean value of Lysotracker staining in untreated cells.

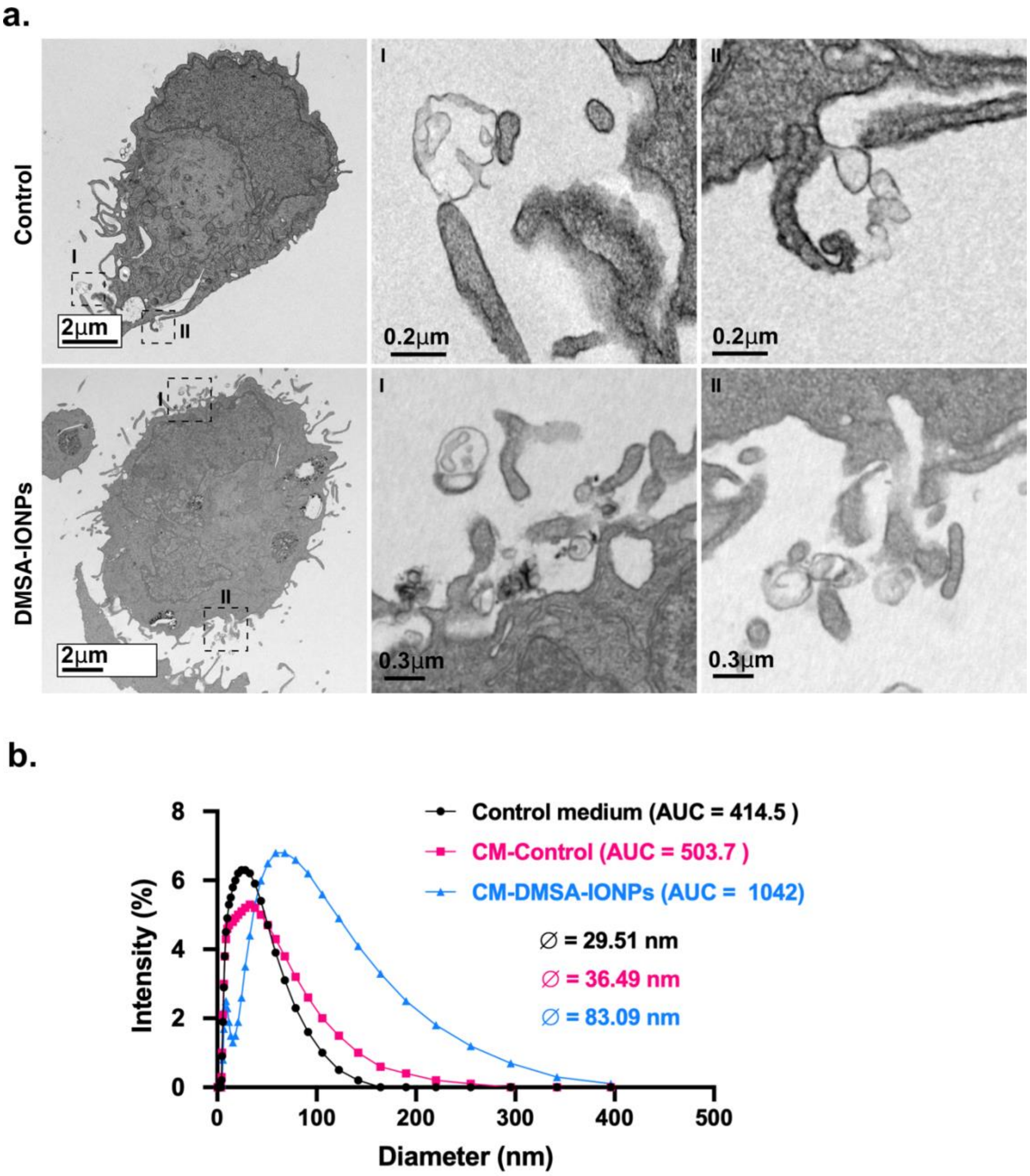

<u>Figure S6</u>. **Representative TEM images of multivesicular body exocytosis in untreated and DMSA-IONP-treated MDA-MB-231 cells. a.** Upper panel shows untreated (Control) cells and the lower panel shows DMSA-IONP treated cells releasing multivesicular bodies. I and II are amplified areas of the dashed boxes. **b.** Analysis of size distribution of particles in the extracellular medium of untreated (CM-Control) and DMSA-IONP-treated (CM-DMSA-IONPs) MDA-MB-231 cells by Dynamic size scattering (DLS). To analyse size distribution, the area under the curve (AUC) was assessed by GraphPad Prism and median values of each peak were extracted as the mean diameter of particles (∅).

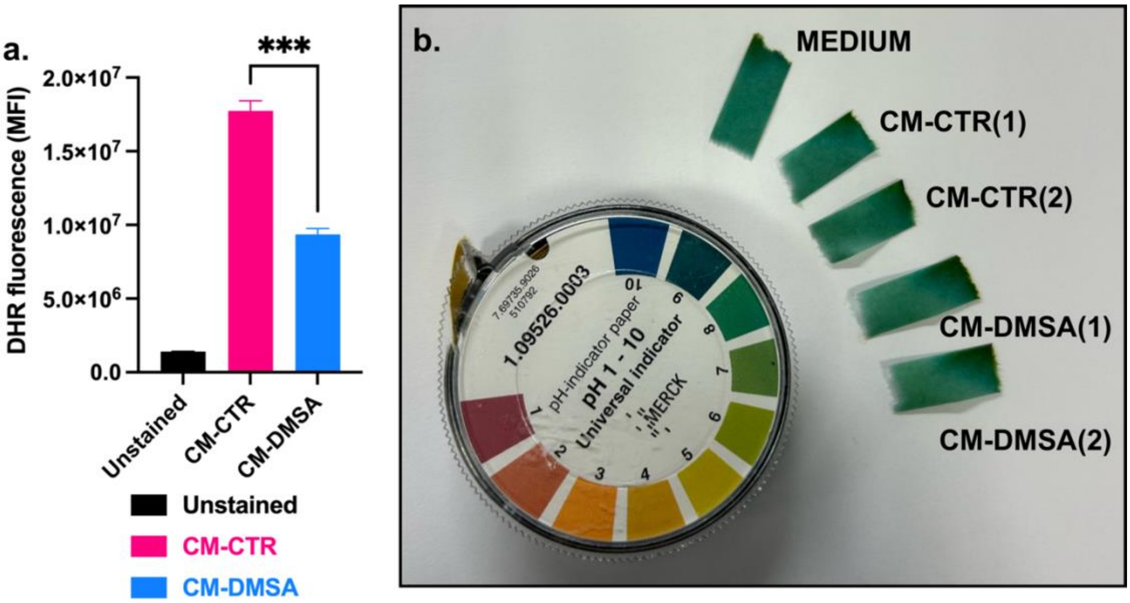

<u>Figure S7</u>. **The amount of ROS and the pH of MDA-MB-231 cell conditioned medium (CM) of untreated cells (CTR) or those treated with DMSA-IONPs (DMSA). a.** Quantification of the amount of ROS in the CM of untreated and DMSA-IONP treated MDA-MB-231 cells using the DHR probe in a SpectraMAX fluorescence reader: DMEM + 10% FBS with no DHR probe, unstained; CM of untreated MDA-MB-231 cells, CM-CTR; CM of DMSA-IONP treated MDA-MB-231 cells, CM-DMSA. **b.** The pH of the different media using pH-indicator strips.

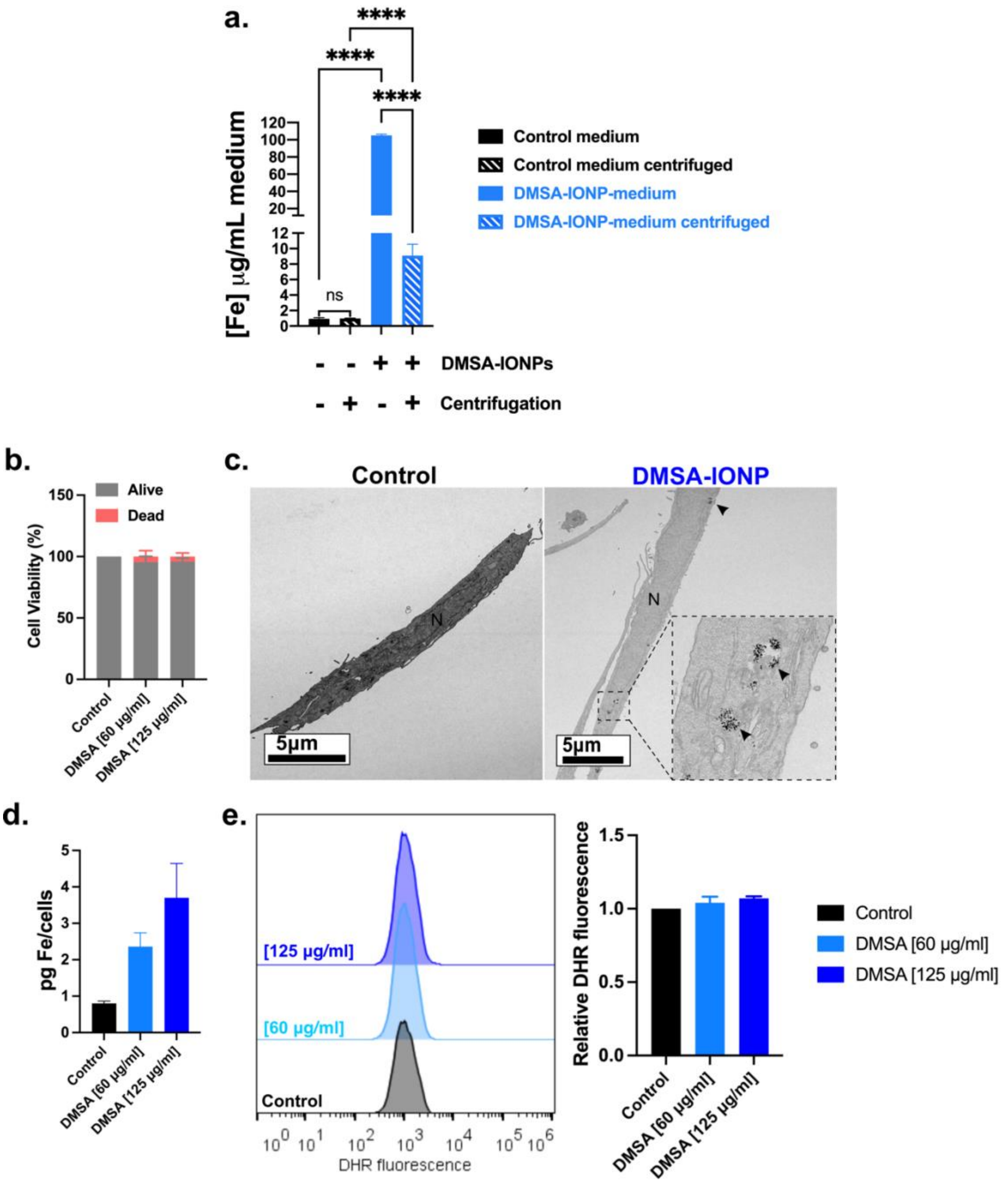

<u>Figure S8</u>. DMSA-IONPs do not affect SVEC4-10 cell viability or induce ROS in SVEC4-10 cells. a. Determination of iron content of the extracellular medium of MDA-MB-231 untreated and DMSA-IONP treated cells by ICP-OES. MDA-MB-231 cells were left untreated of treated with DMSA-IONPs for 24 h. Extracellular medium was collected and left uncentrifuged or centrifuged 15 min at 13000 rpm prior to processing for ICP-OES analysis. **b.** SVEC4-10 cell viability after DMSA-IONP treatment measured by flow cytometry of Annexin/PI stained cells. SVEC4-10 cells were treated with a low [60 µg/ml] or high dose [125 µg/ml] of DMSA-IONPs for 24 h and cell viability was then assessed: live cells, grey; dead cells, pink. **c.** DMSA-IONP internalization and intracellular localization assessed by TEM: nucleus, N; DMSA-IONPs, black arrowheads. Scale bar: 5 µm **d.** DMSA-IONP internalization quantified by ICP-OES to measure the intracellular iron content of the cells. **e.** Flow cytometry assessment of the ROS produced by

untreated SVEC4-10 cells or those treated with DMSA-IONPs stained with the DHR probe: left panel, histogram of DHR fluorescence; right panel, reflects the quantification of DHR fluorescence. The data is the mean ± SD (n = 3). Differences in extracellular iron content were assessed by one-way ANOVA with Tukey's multiple comparison test and differences on cell viability and ROS production with one-way ANOVA followed by Dunnett's multiple comparison test: *p <0.05, **p <0.01, ***p <0.001, ****p <0.0001. When cell viability and ROS production were assessed by one-way ANOVA, no significant differences were evident.

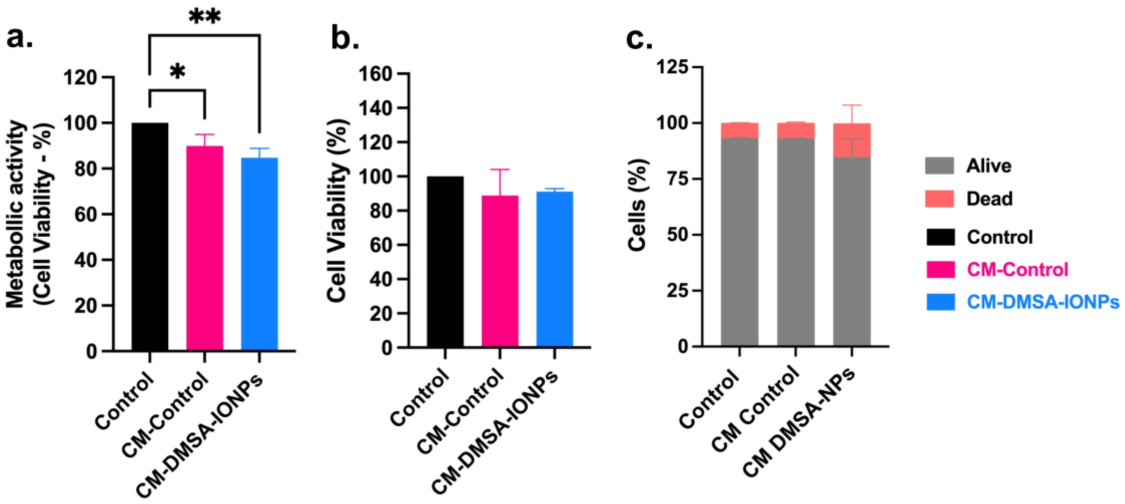

<u>Figure S9</u>. **Indirect effects of CM from untreated or DMSA-IONP treated MDA-MB-231 cells on SVEC4-10 cell viability.** Viability of untreated SVEC4-10 cells (Control), or SVEC4-10 cells treated with CM from untreated MDA-MB-231 cells (CM-Control) or those treated with DMSA-IONPs (CM-DMSA-IONPs), assessed as a read-out of metabolic activity in PrestoBlue assays. **b.** Viability of untreated SVEC4-10 cells (control), or CM-Control and CM-DMSA-IONP treated SVEC4-10 cells assessed by crystal violet staining. **c.** Cell death of untreated SVEC4-10 cells (control), CM-Control and CM-DMSA-IONP treated SVEC4-10 cells assessed by Annexin/PI staining: live cells, grey; dead cells, pink. The data is the mean ± SD (n = 3) and a one-way ANOVA with Dunnett's multiple comparison test was used to analyze the differences in cell viability and the cell death induced between untreated, and CM-Control or CM-DMSA-IONP treated SVEC4-10 cells: *p <0.05, **p <0.01, ***p <0.001, ****p <0.0001.

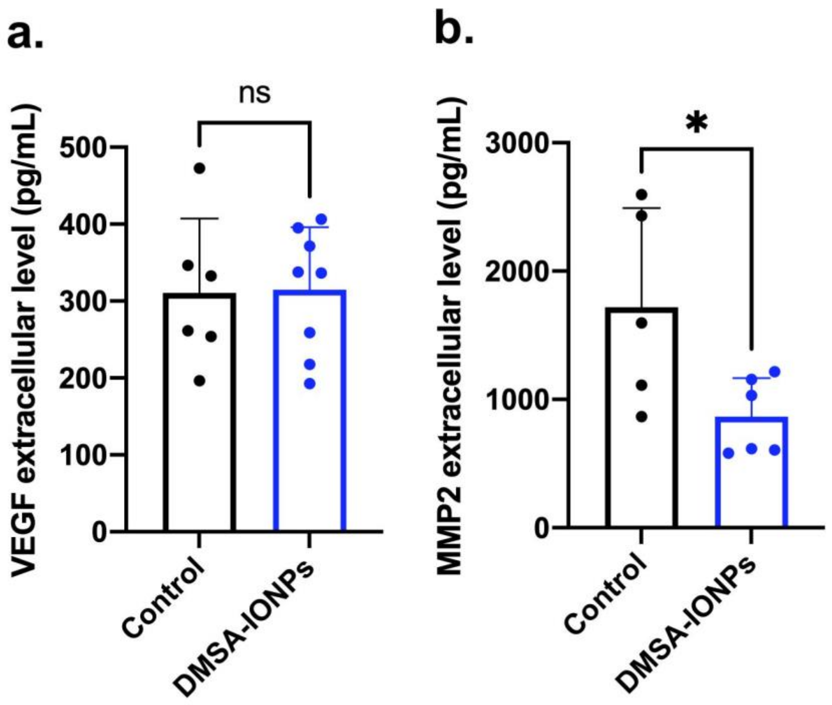

Figure S10. Extracellular content of VEGF and MMP2 of untreated and DMSA-IONP-BC-treated cells. a. VEGF. **b.** MMP2. To assess the differences between extracellular content between untreated and DMSA-IONP-treated cells an unpaired Student's t-test was performed: *p <0.05, **p <0.01, ***p <0.001, ****p <0.0001, ns non-significant.
